# Late‐Stage Amination of Peptides on the Solid Phase

**DOI:** 10.1002/chem.202501229

**Published:** 2025-05-13

**Authors:** Julian Brinkhofer, Marius Werner, Agon Kokollari, Shih‐Yu Pan, Christian Klein, Truc Lam Pham, Franziska Thomas

**Affiliations:** ^1^ Institute of Organic Chemistry Heidelberg University Im Neuenheimer Feld 270 69120 Heidelberg Germany; ^2^ Institute of Pharmacy and Molecular Biotechnology (IPMB) Heidelberg University Im Neuenheimer Feld 364 69120 Heidelberg Germany; ^3^ Department of Chemistry Graduate School of Science The University of Tokyo Bunkyo‐ku Tokyo 113‐0033 Japan

**Keywords:** amines, late‐stage functionalization, peptides, solid‐phase synthesis, tryptophan zippers

## Abstract

Late‐stage peptide modification is a powerful tool for rapidly generating a library of peptide mimetics, for example, for drug discovery or catalyst development. While late‐stage modifications exist for many types of structural features, methods for introducing amines into peptides via a late‐stage approach are rare, despite their enormous potential for the development of peptide therapeutics. Here we present a protocol for introducing amines into peptides by our established on‐resin iodination‐substitution approach. Our method is compatible with a wide variety of amines, including primary and secondary amines, anilines, and other heteroaromatic *N*‐nucleophiles mostly giving good to excellent yields. We introduce amines that are pharmacologically relevant as well as those that can impart catalytic or metal‐binding properties into the peptide of interest. As a proof‐of‐concept study, we introduce the metal ligand tris(2‐aminoethyl)amine (tren) into a tryptophan zipper scaffold using our late‐stage amination approach to explore metal‐induced stapling. Indeed, metal complexation via the tren ligand resulted in a thermal stabilization of more than 30 K in one of our tryptophan zipper designs.

## Graphical abstract

The introduction of amines into peptides via an on‐resin iodination‐substitution reaction sequence provides access to a wide variety of modifications, including amine‐based metal ligands. Primary, secondary, and aromatic amines, as well as heteroaromatic *N*‐nucleophiles, have been successfully incorporated into the reaction sequence. Particularly basic amines or ammonium chlorides can also be used successfully with acid or base additives.

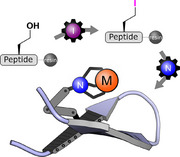

## 1 Introduction

The multifunctional nature of peptides and their easy synthetic accessibility through highly modular solid‐phase peptide synthesis (SPPS) has led to increasing interest in exploring this class of biomolecules as potential therapeutics, building blocks for biomimetic materials and biomimetic catalysts, among other applications.^[^ ^1^, ^2^ ^]^ The possibility of introducing non‐natural peptide modifications by chemical means further enhances functional variety and makes simple and rapid diversification strategies for generating peptide libraries for drug discovery and screening purposes very attractive.^[^ ^3^ ^]^ The classical synthetic approach to peptides with non‐natural modifications involves chemical synthesis of special amino acid building blocks, which are then incorporated into the peptide by SPPS. This is costly, time‐consuming, and limits parallel synthesis approaches. Late‐stage functionalization, on the other hand, offers fast and, if desired, high‐throughput peptide diversification. The most elegant approach, of course, is to modify the deprotected peptide in solution using bioconjugation strategies. However, this limits the scope of chemical modifications that can be introduced into a peptide, as only a relatively small range of chemical reactions proceed with the necessary chemoselectivity. Alternatively, chemical modification can be carried out on the resin‐bound peptide after peptide‐chain assembly, which has the advantage of the functional groups of the peptide being protected, allowing a broader range of solvents and reagents to be used. In addition, an excess of reagents can be used to drive the reaction to completion, and work‐up is easy, as the reaction solutions are simply filtered off.

A straightforward and well‐established late‐stage modification approach is the incorporation of the non‐natural modification by amide bond formation on initially orthogonally protected glutamate, aspartate, or lysine side chains. In recent years, the range of chemical reactions for on‐resin late‐stage peptide modification has been extended to other reaction types, including metal‐catalyzed reactions such as Suzuki–Miyaura cross‐coupling, Cu(I)‐catalyzed azide‐alkyne cycloaddition, C─H activation, hydroboration, and decarboxylative hydroalkylation and arylation, and metal‐catalyst‐free reactions such as Petasis–Borono–Mannich reaction, photocatalyzed deaminative reductive cross‐coupling using Katritzky salts, or the recently published triazine ligation, which expands the range of possible peptide modifications, for example, aliphatic or (hetero)aromatic moieties.^[^ ^4^, ^5^, ^6^, ^7^, ^8^, ^9^, ^10^, ^11^, ^12^ ^]^

**Figure 1:**
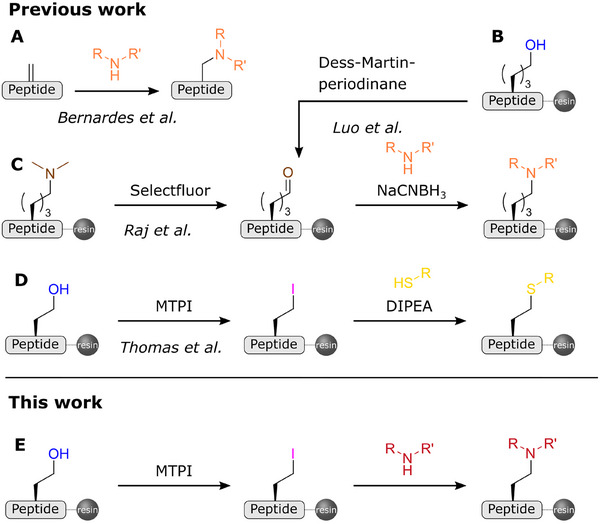
Late‐stage peptide functionalization methods. (A) Aza‐Michael reaction with dehydroalanine‐containing peptides. (B, C) Reductive amination of allysine. (D) Iodination‐substitution approach with thiol nucleophiles. (E) Late‐stage amination by nucleophilic substitution.

Substituted amines are highly relevant modifications for drug development but have also been used for structure‐modulating applications in peptides, such as reversible peptide stapling.^[^ ^13^, ^14^ ^]^ However, the introduction of secondary or tertiary amines into peptides in a late‐stage synthesis has been rarely described. Among the few examples of amination reactions on peptides is the aza‐Michael addition with dehydroalanine‐containing peptides in solution (Figure 1A).^[^ ^15^, ^16^ ^]^ Although this is a powerful method also suitable for protein modification, the reaction produces a mixture of isomers, and only a limited number of amines have been covered in the scope.^[^ ^15^ ^]^ Solid‐phase amination methods include reductive amination on lysine, which can be used to access dipicolylamine‐containing peptides.^[^ ^17^, ^18^, ^19^, ^20^ ^]^ Luo et al. showed that reductive amination can also be used with allysine, which has been prepared by oxidation of 6‐hydroxynorleucine (Figure 1B).^[^ ^21^ ^]^ The method has only been demonstrated on a single peptide using simple amines and is limited by the use of Dess–Martin periodinane as an oxidant, which is poorly tolerated by tryptophan‐containing peptides.^[^ ^22^ ^]^ A recent study by Raj et al. has shown a new approach to prepare allysine‐containing peptides in solution or on water‐swellable Rink amide PEGA resin, followed by on‐resin reductive amination (Figure 1C).^[^ ^23^ ^]^ The same group has very recently published a method for producing allysine‐containing peptides by carbonylation of arginine followed by reductive amination in solution.^[^ ^24^ ^]^

In our laboratory, we have developed an iodination‐substitution approach that allows the introduction of various nucleophiles via an S_N_2 reaction on iodohomoalanine formed on the resin from homoserine in an Appel‐type reaction (Figure 1D).^[^ ^25^ ^]^ While thioether formation works particularly well, the use of amines has been limited by elimination as a side reaction. In this work, we show how various amines, including primary and secondary amines, aniline derivatives, hydrochlorides, and even heteroaromatics, can be incorporated into peptides by nucleophilic substitution in high yields by carefully adjusting the reaction conditions, for example, solvent or addition of base or acid (Figure 1E). As certain amines exhibit excellent metal‐chelating properties, we have also investigated our late‐stage amination approach in the synthesis of metal‐binding peptides with non‐natural metal ligands such as the tris(2‐aminoethyl)amine (tren) ligand. In a proof‐of‐concept study, we investigated metal stapling in a de novo tryptophan‐zipper peptide scaffold.

## 2 Results and Discussion

### 2.1 Optimization of the On‐Resin Late‐Stage Amination

The late‐stage amination presented here is an advance on our previously reported iodination‐substitution approach.^[^ ^25^ ^]^ While this method was particularly powerful for the introduction of aromatic thioethers, the yield in amination reactions was modest at best due to the basic nature of the amine nucleophiles, which promoted the competitive elimination reaction. Given the versatility of amines as a functional group and the numerous possibilities for their further modification, we set out to identify conditions that would enable high‐yield late‐stage amination based on the iodination‐substitution approach.

To this end, we chose the influenza matrix protein M1‐derived peptide epitope **P1─OH** (OH stands for homoserine, peptide sequence see Table 1) as a test peptide because it contains a wide range of amino acid residues and functional groups and has an intermediary chain length of 12 amino acids.^[^ ^26^ ^]^

**Table 1:** Sequences of peptides subjected to late‐stage amination. **X**: position of Dmt‐protected homoserine.

Peptide	Sequence
**P1** ^[a]^	H‐GP **X** KAEIAQRLE‐NH_2_
**P2**	Ac‐K **X** K‐NH_2_
**P3**	Ac‐ **X** PYKCPECGKSFSQSSNLQKHQRTH‐NH_2_
**P4**	Ac‐KLPPGWEKR *Nle* SRSSGRVYYFNHITNASQ **X** ERPSG‐NH_2_
**P5** ^[a]^	H‐KAPRKQ **X** ATKAARMSAPSTGGVKKPHR‐OH
**P6**	Ac‐SW **X** WENGKWHWH‐NH_2_
**P7**	Ac‐SWHWENGKW **X** WH‐NH_2_

^[a]^ **P1** and **P5** were *N*‐terminally protected with a *tert*‐butoxycarbonyl group.

Rink‐amide MBHA polystyrene resin was used in microwave‐assisted SPPS due to its low cost and widespread use, with the exception of **P5**, which was synthesized on Wang resin. The iodination was performed as previously reported by deprotection of dimethoxytrityl (Dmt)‐protected homoserine with 1% trifluoroacetic acid (TFA) in dichloromethane, followed by on‐resin iodination with methyltriphenoxyphosphonium iodide (MTPI) to give the iodohomoalanine‐containing peptide **P1─I**.^[^ ^25^, ^27^ ^]^ Tris(2‐aminoethyl)amine (**N1**) was used as the amine nucleophile for our rounds of optimization because of its basicity, which should significantly promote the elimination side reaction (Table 2). The conditions for the substitution reaction in our initial study were described for peptides linked to ChemMatrix resin, which has been discontinued. As the polystyrene resin has a lower swelling capacity, we were able to reduce the volume of the reaction solutions.

**Table 2:** Optimization of the late‐stage amination of **P1─OH** using tris(2‐aminoethyl)amine.

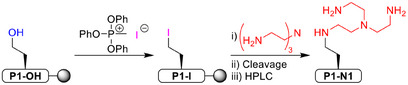					
Entry^[a]^	Solvent	Temperature	Time	Additive	Yield^[b]^
1	DMF	Room temperature	16 hours	–	18%
2	DMSO	Room temperature	16 hours	–	20%
3	EtOH/DMF	Room temperature	16 hours	–	32%
4	Pyridine	Room temperature	16 hours	–	37%
5	MeCN	Room temperature	16 hours	–	44%
6^[c]^	MeCN	Room temperature	16 hours	–	67%
7^[d]^	MeCN	Room temperature	16 hours	–	35%
8^[e]^	MeCN	95 °C	90 seconds	–	43%
9^[f]^	MeCN	Room temperature	16 hours	HCOOH	92%
10^[f]^	DMF	Room temperature	16 hours	HCOOH	71%

^[a]^ 5 µmol of resin‐bound **P1─OH** was allowed to react with a 1 m amine solution at the given reaction conditions;

^[b]^ Yields are the ratio of the purity of the modified peptide to the unmodified peptide. Peptide purities were determined by RP‐HPLC. See the Supporting Information for details;

^[c]^ A 0.25 m solution was used;

^[d]^ A 2.5 m solution was used;

^[e]^ Reaction was performed in a microwave reactor at 300 W;

^[f]^ 7.5 equiv of formic acid related to peptide were added. For HPLC traces, see Figures S1–S3 (Supporting Information).

In our initial study, substitution reactions on resin‐bound iodohomoalanine were carried out in *N,N*‐dimethylformamide (DMF).^[^ ^25^ ^]^ However, when using these conditions with **N1** as the nucleophile, **P1─N1** was obtained in only 18% yield due to the elimination side reaction (Table 2, entry 1). Therefore, our initial efforts focused on identifying a solvent for this reaction that would suppress the elimination reaction (Table 2, entries 2–5). It was found that acetonitrile gave the best results, increasing the yield of on‐resin amination by more than a factor of two compared to DMF. On further variation of the conditions, we found that when using basic amines such as **N1**, which promote the elimination reaction, reducing the amine concentration further increased the yield of the amination product, which we attribute to the reduced basicity of the solution (Table 2, entry 6). We also explored microwave heating at 95 °C (300 W) for 90 seconds and obtained comparable results to the overnight reaction at room temperature (Table 2, entry 8). These conditions have previously been used for microwave‐assisted peptoid synthesis, a substitution reaction on resin‐bound bromoacetic acid with amines.^[^ ^28^ ^]^ Finally, we found that the addition of 150 mm formic acid resulted in an almost quantitative yield, which we attribute to a buffering effect of the formic acid, thereby reducing the elimination reaction (Table 2, entry 9). This result was reproducible when DMF was used as a solvent (Table 2, entry 10), providing a suitable alternative for basic amines that are poorly soluble in acetonitrile. In order to best address both basic amines, such as **N1,** and less reactive amines, such as aniline derivatives, in our following Scope and Limitations study, we decided to use the conditions given in Table 2, entry 5, as our standard conditions. We also decided to avoid microwave conditions for our further studies in order to keep the experimental setup as simple as possible, without the need for special equipment.

### 2.2 Scope and Limitations

**Figure 2:**
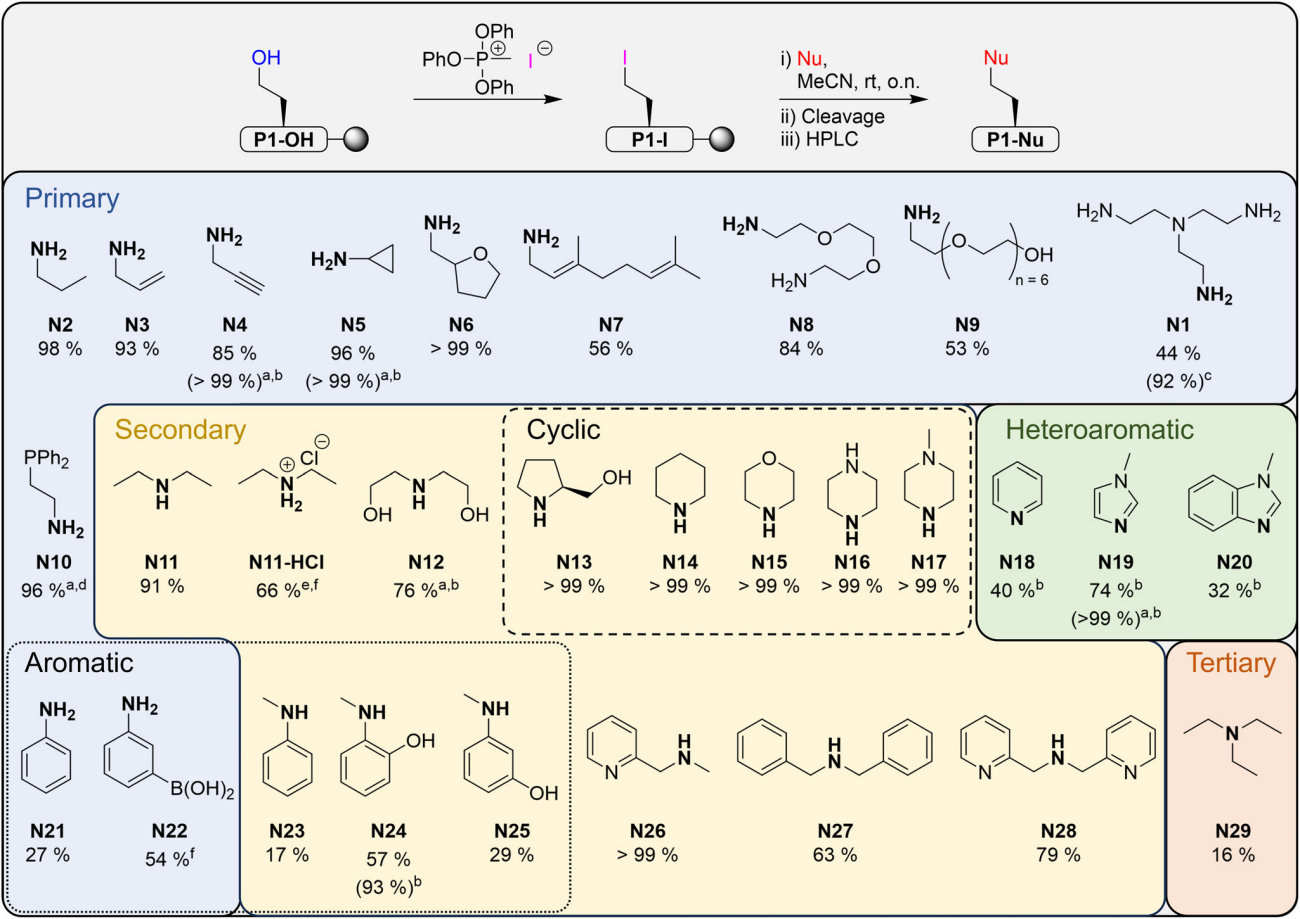
Scope of the late‐stage amination by iodination and substitution of peptide **P1─OH**. Yields are the ratio of the purity of the modified peptide to the unmodified peptide. Peptide purities were determined by RP‐HPLC. See the Supporting Information for details. Under standard conditions, peptide **P1─I** was allowed to react with a 1 m solution of the amine nucleophile at room temperature for approximately 16 hours. (a) 64 hours reaction time. (b) 2.5 m amine solution was used. (c) 150 mM formic acid was added. (d) 140 mm DBU was added. (e) DMF was used as solvent. For HPLC traces, see Figures S1–S34 (Supporting Information).

Having identified suitable standard conditions, we explored the scope and limitations of late‐stage amination (Figure 2). Generally, primary and secondary cyclic amines perform best in late‐stage amination with consistently near‐quantitative yields. Aliphatic, non‐cyclic secondary amines still give good to excellent yields, depending on the steric bulk around the nitrogen center. As expected, the generally less reactive anilines resulted in moderate to low yields under the identified standard conditions. Amination of **P1‐I** with **N21** gave the amination product in 27% yield, and activated aromatic amines **N22** and **N24** resulted in good yields of 54% and 57%, respectively. Further increasing the concentration to 2.5 m gave a yield of 93% for **N24**. Extending the reaction time should give similar results, as significant amounts of unreacted **P1─I** were observed in the HPLC traces of all reactions with unreactive amines (Figure S4). The heteroaromatic *N*‐nucleophiles **N18**–**N20** gave moderate to good yields at an increased concentration of 2.5 M and, in the case of **N19**, excellent yields when the reaction time was further extended to 64 hours. It should be emphasized that imidazolium salts such as **N19** and **N20** are particularly interesting modifications, as they have previously been used to prepare *N*‐heterocyclic carbene complexes with Ir, Os, Rh, and Ru as potential anticancer agents.^[^ ^29^, ^30^ ^]^ In summary, as long as the *N*‐nucleophile is not too basic, for example, **N1** but unreactive, it is advisable to increase the concentration and reaction time to improve the yield. Interestingly, even the basic and bulky triethylamine (**N29**), which is considered to be non‐nucleophilic, reacted in the on‐resin late‐stage amination, albeit with a low yield of 16%, again with the potential for higher yields with prolonged reaction times.

Another observation from our scope and limitations study is that our method is tolerant to other functional groups such as phosphines, boronic acids, and alcohols or phenols. Thus, we were able to selectively introduce 2‐(diphenylphosphaneyl)ethan‐1‐amine (**N10**), which has been used, for example, by the Ward group to construct an artificial Suzukiase with streptavidin^[^ ^31^ ^]^ or di(2‐picolyl)amine (**N28**), which has been used as a ligand in various peptides.^[^ ^17^, ^18^, ^20^, ^32^, ^33^, ^34^, ^35^, ^36^, ^37^, ^38^ ^]^ We also found that secondary amino groups react highly selectively over pyridine moieties. We confirmed this result by one‐ and two‐dimensional NMR experiments on the product of the late‐stage amination of **P2** with **N26**, finding evidence only for the tertiary amine to be formed and not the pyridinium product (Figures S35–S41).

Many structurally interesting amines are commercially available only as the corresponding hydrochlorides. Therefore, we sought to identify conditions under which the hydrochlorides could also be used in the late‐stage amination (Figure S34). Interestingly, when **N11─HCl** was used in DMF, the chlorohomoalanine‐containing peptide **P1─Cl** was formed in almost quantitative yield. The addition of DIPEA (1.0 m) to the reaction solution led to only minor improvement, with the desired amination product **P1─N11** being the minor product along with **P1─Cl** in a yield of 19%. The addition of a stronger base, DBU (1.0 m), or an increase in the amount of DIPEA (10 m) did not significantly improve the yield of **P1─N11**, with DBU mainly inducing the formation of elimination product **P1═**. In an attempt to suppress the elimination while ensuring sufficient activation of the nucleophile, we found that the addition of small amounts of DBU (140 mm) significantly improved the yield of **P1─N11** up to 66%.

**Figure 3:**
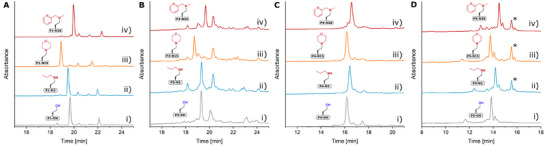
HPLC traces of crude aminated peptides obtained by on‐resin late‐stage iodination‐substitution. (A) **P1**, (B) **P3**, (C) **P4**, and (D) **P5**. (i) Unmodified peptide **P─OH**. (ii) Amination with **N2**. (iii) Amination with **N15**. (iv) Amination with **N26**. *Unreacted iodohomoalanine‐containing peptide species.

Having explored the scope of *N*‐nucleophiles for on‐resin late‐stage amination, we turned to varying the peptides to see if this method was generally applicable even to more complex peptides. For example, peptides **P3**, a zinc‐finger peptide,^[^ ^39^ ^]^ **P4**, derived from the hPin1 WW domain,^[^ ^40^ ^]^ and the histone mutant H3.3_K27M derived peptide epitope **P5** ^[^ ^41^ ^]^ cover most of the canonical amino acids. To this end, the late‐stage amination was exemplarily carried out with **N2**, **N15**, and **N26** using the standard conditions (Table 2, entry 5). The late‐stage amination at **P3** gave good amination yields in the range of 75% to 90% for all reactions, amination reactions at **P4** were almost quantitative and amination of **P5** was successful with yields in the range of 70%–80%. The reaction was incomplete in case of **P5**, as the iodohomoalanine‐containing peptide was not fully converted. The yield may be improved by extending the reaction time (Figure 3). It should be noted that the determination of yields is subject to inaccuracies due to the overlap with other peaks in the HPLC traces.

Overall, the developed method allows the rapid, simple, and selective modification of peptides with a broad range of primary and secondary amines. Late‐stage aminations with anilines and heteroaromatic *N*‐nucleophiles are also feasible, albeit at higher concentrations and longer reaction times. The elimination reaction that occurs when using basic amines can be suppressed in favor of the amination product by adding small amounts of formic acid to the reaction mixture.

### 2.3 Metal‐Stapling with Modified β‐Hairpin Peptides

**Figure 4:**
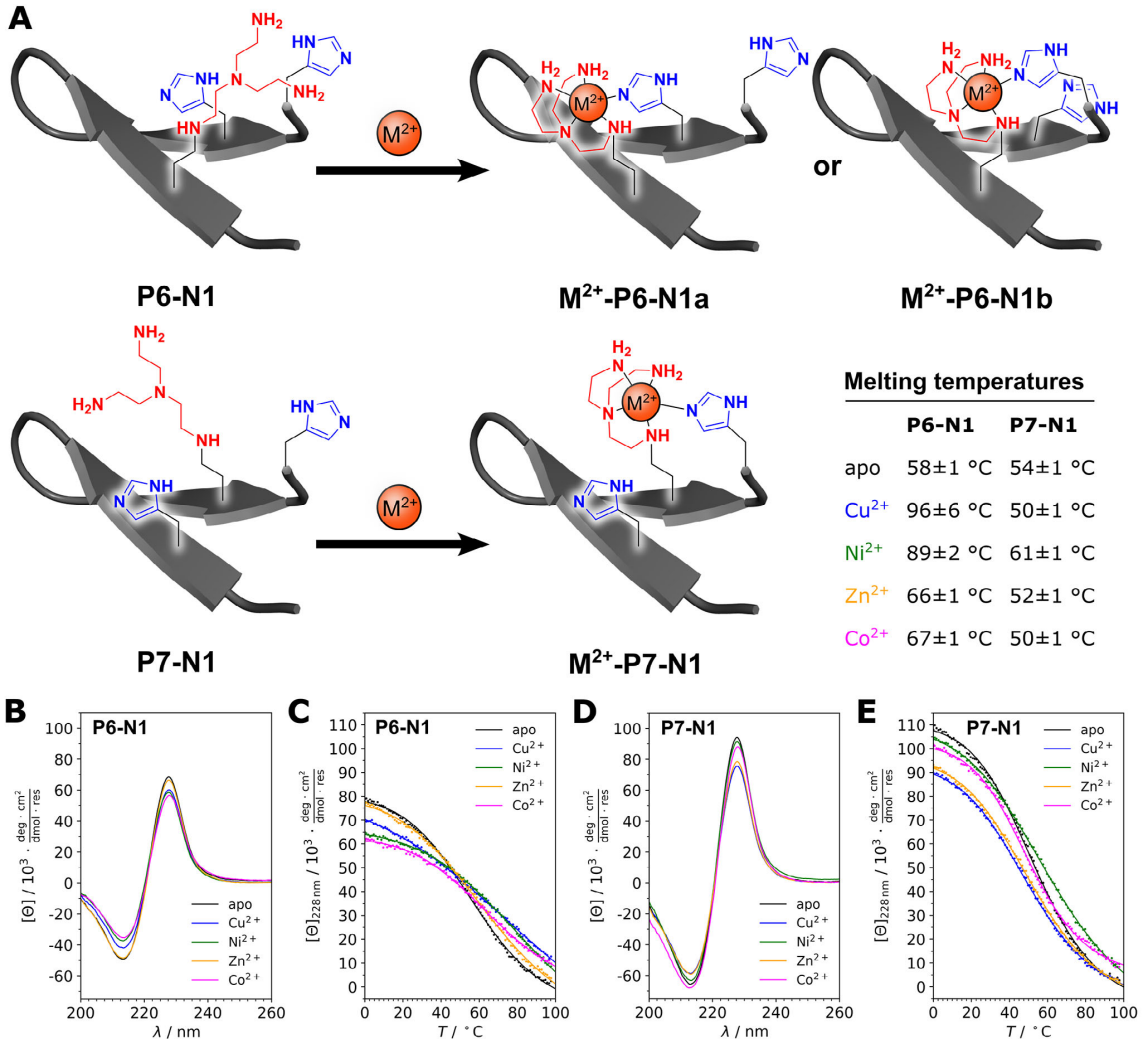
Metal stapling of tryptophan‐zipper peptides **P6─N1** and **P7─N1**. (A) Proposed binding behavior of **P6─N1** and **P7─N1** with Cu^2+^, Zn^2+^, and Co^2+^. (B) CD spectra of **P6─N1** at 20 °C in the absence and presence of metal ions Co^2+^, Ni^2+^, Cu^2+^, and Zn^2+^. (C) Thermal denaturation profiles of **P6─N1** measured at 228 nm in the absence and presence of metal ions Co^2+^, Ni^2+^, Cu^2+^, and Zn^2+^. (D) CD spectra of **P7─N1** at 20 °C in the absence and presence of metal ions Co^2+^, Ni^2+^, Cu^2+^, and Zn^2+^. (E) Thermal denaturation profiles of **P7─N1** measured at 228 nm in the absence and presence of metal ions Co^2+^, Ni^2+^, Cu^2+^, and Zn^2+^. All measurements were performed in aqueous buffer at pH 7.4 (50 µM peptide, 50 µM metal ions, 10 mm MOPS, 150 mm NaCl) in a 1 mm quartz cuvette.

The established on‐resin late‐stage amination allows for a large variety of amine residues and other *N*‐nucleophiles to be introduced into peptides. Among these are pharmacologically relevant amines or those that confer catalytic activity or metal‐binding ability to the peptide of interest.^[^ ^13^, ^20^, ^29^, ^30^, ^31^, ^42^, ^43^, ^44^ ^]^ Our group is generally interested in metal‐binding peptides as they have potential applications in sensing but also in catalysis.^[^ ^25^, ^45^ ^]^ Therefore, we aimed to investigate the introduction of a metal ligand such as tren (**N1**) into a miniprotein scaffold, for example, to explore its potential in metal stapling (Figure 4). Previously, we had designed the metal binding tryptophan zipper Tz2H_3_, which displays metal binding consisting of three histidine (His) residues.^[^ ^45^ ^]^ We decided to create two variants thereof in which His3 (Table 1, **P6**) or His10 (Table 1, **P7**) is replaced by homoserine as an anchor site for **N1** to be introduced through late‐stage amination. **N1** is a tetradentate *N*‐donor that has been studied in various metal‐binding applications, mostly with copper.^[^ ^46^, ^47^, ^48^, ^49^, ^50^, ^51^, ^52^ ^]^ It has been found that copper complexes with derivatives of **N1** can successfully catalyze the cycloaddition of azides with terminal alkynes under aerobic conditions.^[^ ^53^ ^]^ Another complex with a derivative of **N1** can reversibly form end‐on superoxo‐Cu(II) complexes and may therefore be of interest in the design of minienzymes with oxidase and oxygenase activity.^[^ ^54^, ^55^, ^56^ ^]^ However, to our knowledge, **N1** has not yet been incorporated into peptides as a ligand.

The late‐stage amination of **P6** and **P7** with **N1** proceeded in good yields using the conditions described in Table 1, entry 9, which are suitable for basic amines. The metal‐stapling properties of **P6─N1** and **P7─N1** were examined by CD spectroscopy (Figure 4).

The CD spectra of **P6─N1** (Figure 4B) and **P7─N1** (Figure 4D) are characteristic of a tryptophan zipper with a pronounced maximum at 228 nm resulting from the exciton coupling of the tryptophan residues. The exciton signals of **P7‐N1** are generally more intense than those of **P6─N1**, indicating subtle differences in the secondary structure of the two apopeptides. Upon addition of Co^2+^, Ni^2+^, Cu^2+^, or Zn^2+^, the CD spectra in all cases showed changes in intensity indicative of metal binding. We also recorded CD thermal denaturation profiles to determine the thermal stabilities of **P6─N1** and **P7─N1** in their apo and holo states. **P6─N1** is stably folded with a melting temperature (*T*_m_) of 58 °C (Figure 4C). Upon addition of metal ions to **P6─N1**, a strong increase in *T*_m_ was observed, especially for Co^2+^, Ni^2+^, and Cu^2+^. Only a minor increase in *T*_m_ was observed for Zn^2+^. In contrast, **P7─N1**, which displays a *T*_m_ of 54 °C in the apo state, was only marginally stabilized by the addition of metal ions (Figure 4E). Only the addition of Ni^2+^ induced a small but significant increase of the *T*_m_ (7 K).

We suggest that the distinct properties of **P6─N1** and **P7─N1** are due to the different positions of **N1** in relation to the histidine residues. While in **P6─N1** both histidine residues that could participate in metal binding are on the same strand of the β‐sheet, in **P7─N1** they are on opposite strands. **N1** is known to preferentially form trigonal‐bipyramidal Cu(II) complexes with a monodentate ligand, which we believe is fundamental to the observed results.^[^ ^57^, ^58^ ^]^ Complexes of tren with Ni^2+^, on the other hand, are usually observed in octahedral geometry, with trigonal‐bipyramidal geometries being the exception.^[^ ^59^, ^60^, ^61^ ^]^ We therefore hypothesize that in the case of **P7─N1** the metal complex **M(II)─P7─N1** is formed by metal binding between **N1** and His12, whereas in the case of **P6─N1**, the metal complex could be formed with either His10 (**M(II)─P6─N1a**) or His12 (**M(II)─P6─N1b**) (Figure 4A). Only the formation of a metal complex across the strands should significantly stabilize the structure by metal stapling.

## 3 Conclusion

A reliable method for the on‐resin late‐stage amination of peptides by an iodination‐substitution reaction sequence has been developed. Previously, elimination was a significant limitation when using amines as nucleophiles in this reaction sequence, but we are now able to suppress this undesired side reaction by using acetonitrile as a solvent, adjusting the concentration of the nucleophile, and adding formic acid if particularly basic amines are used in the reaction. A wide scope of amines has been successfully applied in the late‐stage amination approach, including primary and secondary amines, anilines, and heteroaromatic *N*‐nucleophiles. While primary and secondary amines react with very good to excellent yields, anilines and heteroaromatic *N*‐nucleophiles give low to moderate yields under standard conditions. However, these yields can be improved by increasing the amine concentration and/or reaction time. Particularly basic amines, which promote the undesired elimination side reaction, could also be used successfully when formic acid was added in small amounts. Our method is limited with respect to the use of ammonium chlorides as reactants, since the halogen exchange product, chlorohomoalanine, is observed as the main product under standard conditions. This can be largely suppressed by adding DBU to the reaction solution, but the yield is lower compared to using the free amine. The applicability of the method to other peptide sequences of varying complexity was demonstrated through representative late‐stage aminations of a 34‐amino acid WW domain, a cysteine‐containing zinc finger peptide, and the methionine‐containing H3.3_K27M peptide epitope, covering most of the canonical amino acids.

The introduction of amines into peptides is highly attractive, as many amine moieties have high pharmacological relevance or can confer metal‐binding properties to peptides.^[^ ^13^, ^42^, ^43^, ^44^ ^]^ We have demonstrated the latter in a metal‐stapling approach on variants of the tryptophan zipper peptide Tz2H_3_ incorporating the tren ligand (**N1**). It was found that metal stapling occurs with Co^2+^, Ni^2+^, and Cu^2+^ when His3 is substituted, whereas substitution of His10 does not lead to structural stabilization upon metal binding.

Late‐stage diversification of peptides on the resin has great potential for high‐throughput generation of peptide libraries with non‐natural modifications.^[^ ^3^ ^]^ Although many reactions have been established to modify peptides on the resin, including CH activation, Suzuki–Miyaura coupling, or hydroboration, methods to introduce amines into peptides are less developed.^[^ ^4^, ^5^, ^6^ ^]^ To our knowledge, reductive amination has been successfully applied in this context, but the scope of amines and peptides presented in these studies was usually very limited.^[^ ^21^, ^23^ ^]^ Our iodination‐substitution approach, now adapted for amines and other *N*‐nucleophiles, fills this gap and opens up the possibility of introducing a large proportion of amine diversity into peptides using this simple method.

## Supporting Information

The authors have cited additional references within the Supporting Information.^[^ ^62^, ^63^, ^64^, ^65^, ^66^ ^]^

## Conflict of Interests

The authors declare no conflict of interest

## Supporting information



Supporting Information

## Data Availability

The data that support the findings of this study are available in the Supporting Information of this article.
