# Supplementary material for: Late‐Stage Amination of Peptides on the Solid Phase
Source: Chemistry. 2025 May 13;31(34):e202501229. doi: 10.1002/chem.202501229 (PMC12172594; doi:10.1002/chem.202501229)
Supplement: Supplementary file 1 — Supporting Information [file CHEM-31-e202501229-s001.pdf]

Table of Contents

1 Supplementary figures 1

2 Experimental procedures 56

2.1 Reagents and solvents 56

2.2 Software for data analysis and visualization 56

2.3 Peptide synthesis, functionalization and purification 56

2.4 CD spectroscopy 63

2.5 NMR spectroscopy 63

2.6 Thin layer and column chromatography 63

3 Organic Synthesis 64

3.1 Fmoc-Hse-OH 64

3.2 Fmoc-Hse(Dmt)-OH 65

4 References 67

1 Supplementary figures

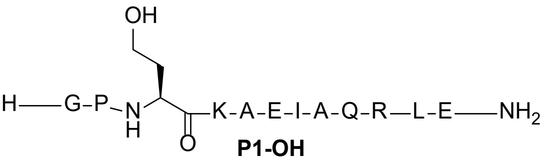

P1-OH

Chemical Formula: $C_{56}H_{98}N_{18}O_{18}$
Exact Mass: 1310.73
Molecular Weight: 1311.51

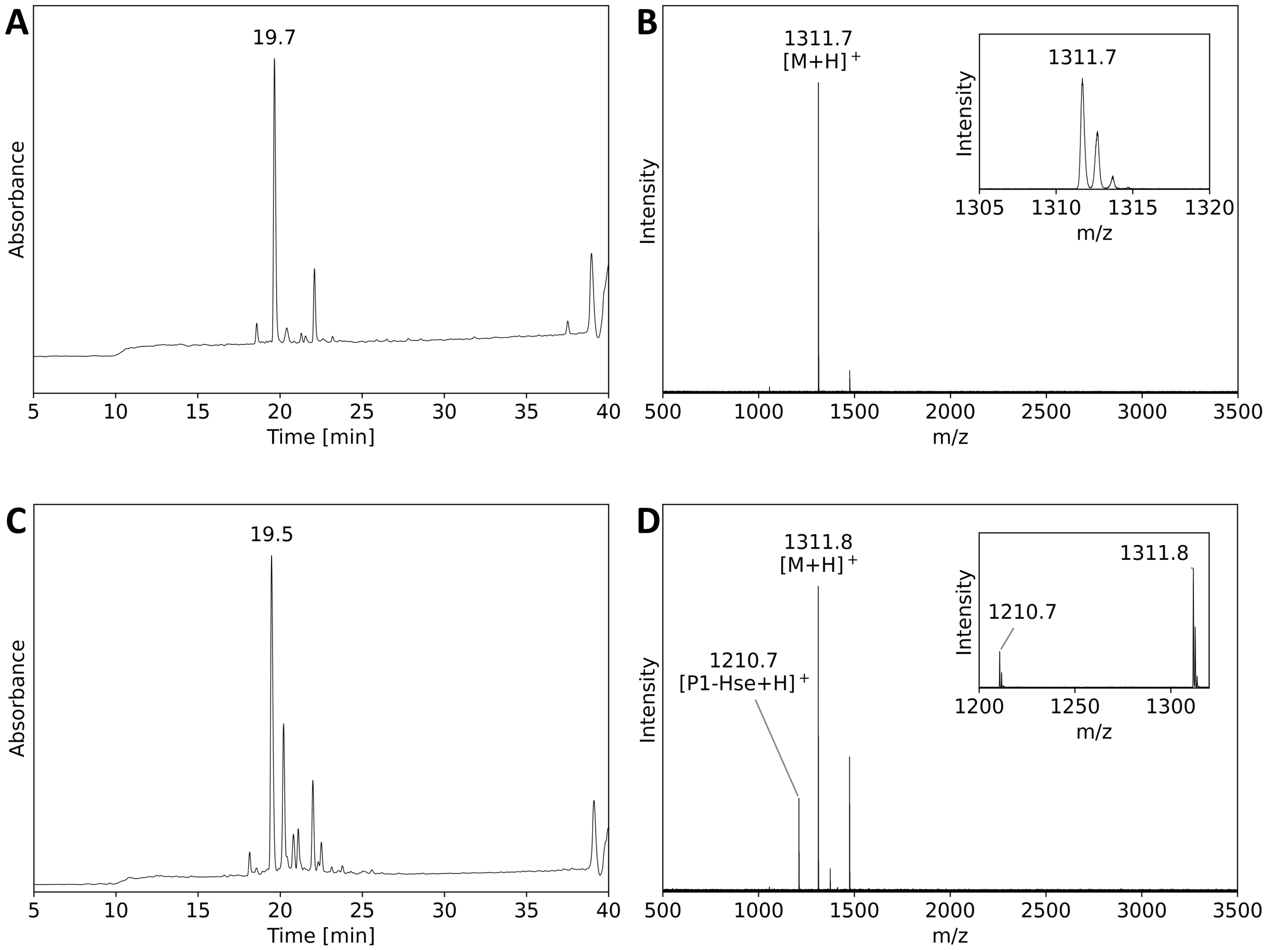

Figure S1: **P1-OH** A) Analytical HPLC (220 nm) of crude peptide (Batch 1). B) MALDI-TOF MS of crude peptide (Batch 1). C) Analytical HPLC (220 nm) of crude peptide (Batch 2). B) MALDI-TOF MS of crude peptide (Batch 2). Calculated mass: $[M+H]^+$ 1311.7, $[P1\text{-}Hse+H]^+$ 1210.7.

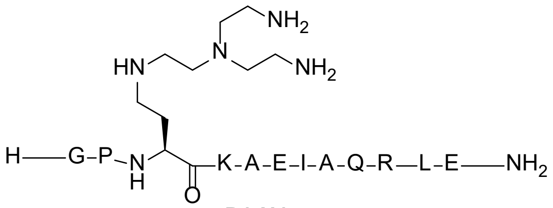

P1-N1
Chemical Formula: $C_{62}H_{114}N_{22}O_{17}$
Exact Mass: 1438.87
Molecular Weight: 1439.73

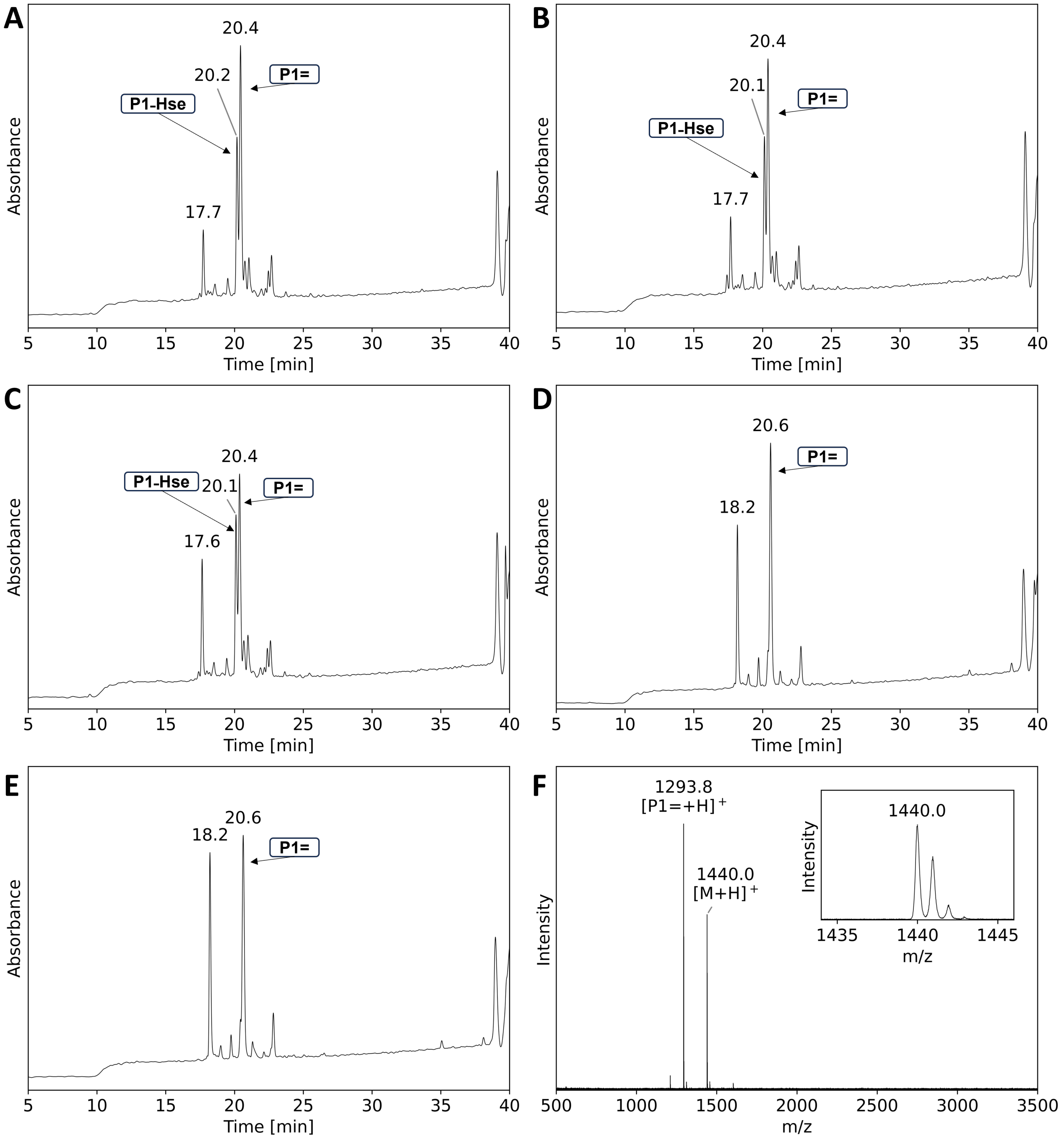

Figure S2: **P1-N1** A) Analytical HPLC (220 nm) of crude peptide (from **P1-OH** Batch 2; Table 2 Entry 1). B) Analytical HPLC (220 nm) of crude peptide (from **P1-OH** Batch 2; Table 2 Entry 2). C) Analytical HPLC (220 nm) of crude peptide (from **P1-OH** Batch 2; Table 2 Entry 3. D) Analytical HPLC (220 nm) of crude peptide (from **P1-OH** Batch 1; Table 2 Entry 4). E) Analytical HPLC (220 nm) of crude peptide (from **P1-OH** Batch 1; Table 2 Entry 5). F) MALDI-TOF MS of crude peptide (from **P1-OH** Batch 1; Table 2 Entry 5). Calculated mass: $[P1{=}{+}H]^+$ 1293.7, $[M+H]^+$ 1439.9.

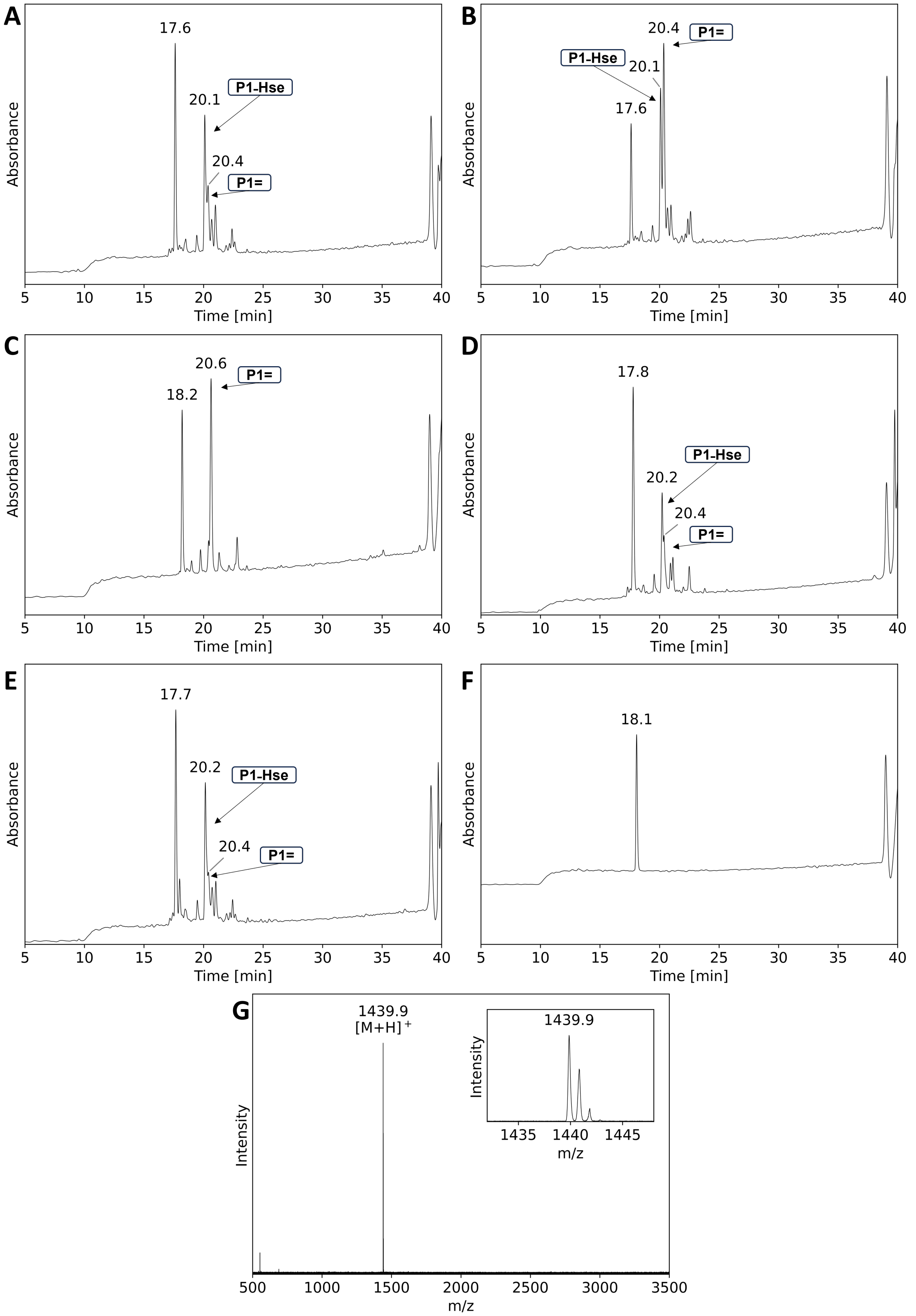

Figure S3: **P1-N1** A) Analytical HPLC (220 nm) of crude peptide (from **P1-OH** Batch 2; Table 2 Entry 6). B) Analytical HPLC (220 nm) of crude peptide (from **P1-OH** Batch 2; Table 2 Entry 7). C) Analytical HPLC (220 nm) of crude peptide (from **P1-OH** Batch 1; Table 2 Entry 8). D) Analytical HPLC (220 nm) of crude peptide (from **P1-OH** Batch 2; Table 2 Entry 9). E) Analytical HPLC (220 nm) of crude peptide (from **P1-OH** Batch 2; Table 2 Entry 10). F) Analytical HPLC (220 nm) of purified peptide. G) MALDI-TOF MS of purified peptide. Calculated mass: $[M+H]^+$ 1439.9.

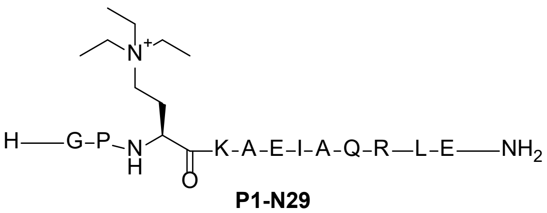

P1-N29

Chemical Formula: $C_{62}H_{112}N_{19}O_{17}^{+}$

Exact Mass: 1394.85

Molecular Weight: 1395.69

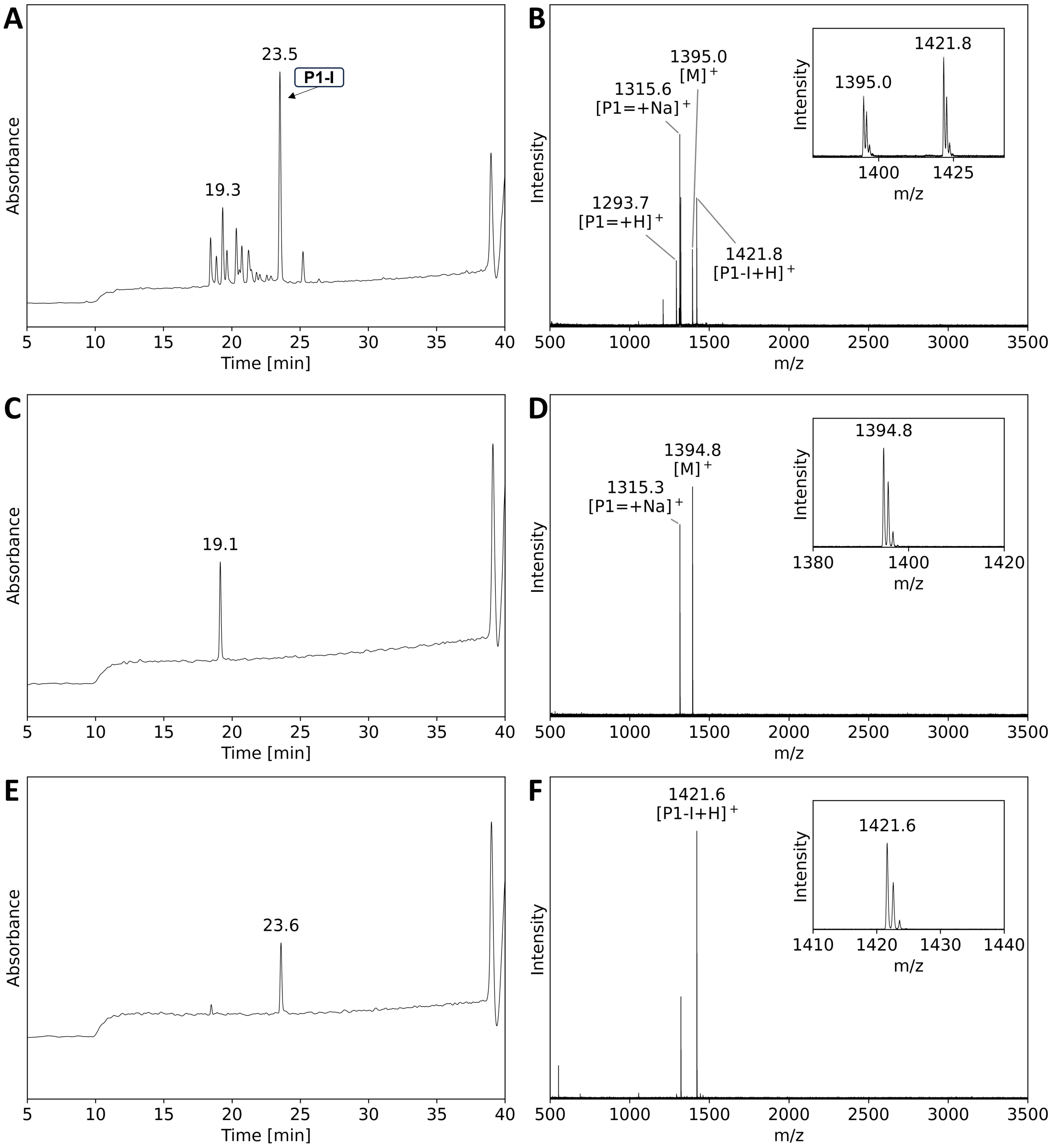

Figure S4: **P1-N29** A) Analytical HPLC (220 nm) of crude peptide (from **P1-OH** Batch 1). B) MALDI-TOF MS of crude peptide (from **P1-OH** Batch 1). C) Analytical HPLC (220 nm) of purified peptide. D) MALDI-TOF MS of purified peptide E) Analytical HPLC (220 nm) of purified **P1-I**. F) MALDI-TOF MS of purified **P1-I**. Calculated mass: $[P1=+H]^{+}$ 1294.7, $[P1=+Na]^{+}$ 1315.7, $[M]^{+}$ 1394.8, $[P1\text{-}I+H]^{+}$ 1421.6.

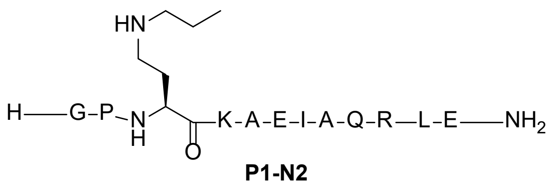

P1-N2

Chemical Formula: $C_{59}H_{105}N_{19}O_{17}$

Exact Mass: 1351.79

Molecular Weight: 1352.61

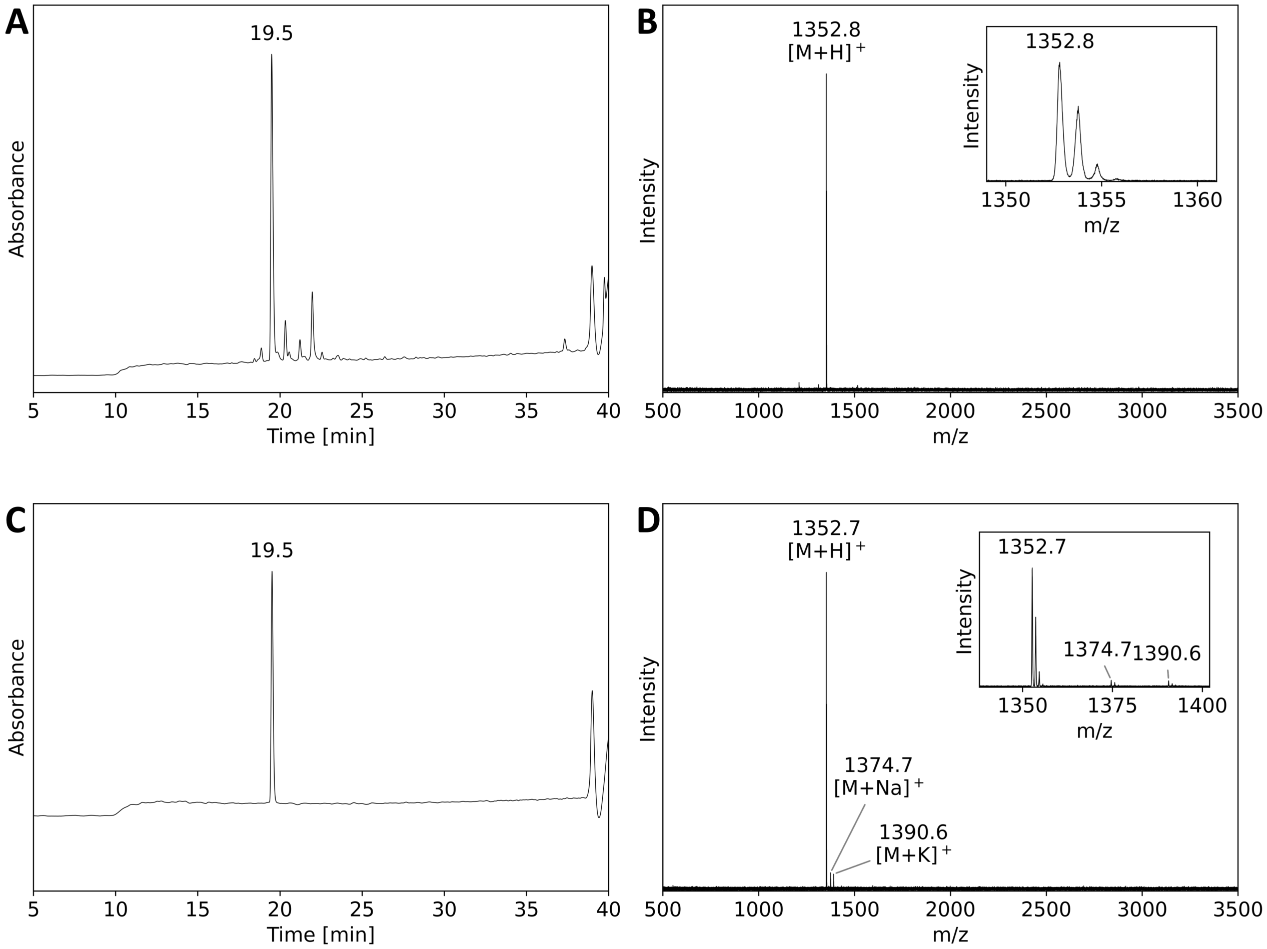

Figure S5: **P1-N2** A) Analytical HPLC (220 nm) of crude peptide (from **P1-OH** Batch 1). B) MALDI-TOF MS of crude peptide (from **P1-OH** Batch 1). C) Analytical HPLC (220 nm) of purified peptide. D) MALDI-TOF MS of purified peptide. Calculated mass: $[M+H]^+$ 1352.8, $[M+Na]^+$ 1374.8, $[M+K]^+$ 1390.8.

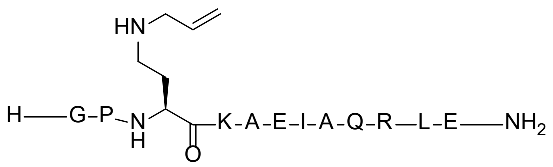

P1-N3

Chemical Formula: $C_{59}H_{103}N_{19}O_{17}$
Exact Mass: 1349.78
Molecular Weight: 1350.59

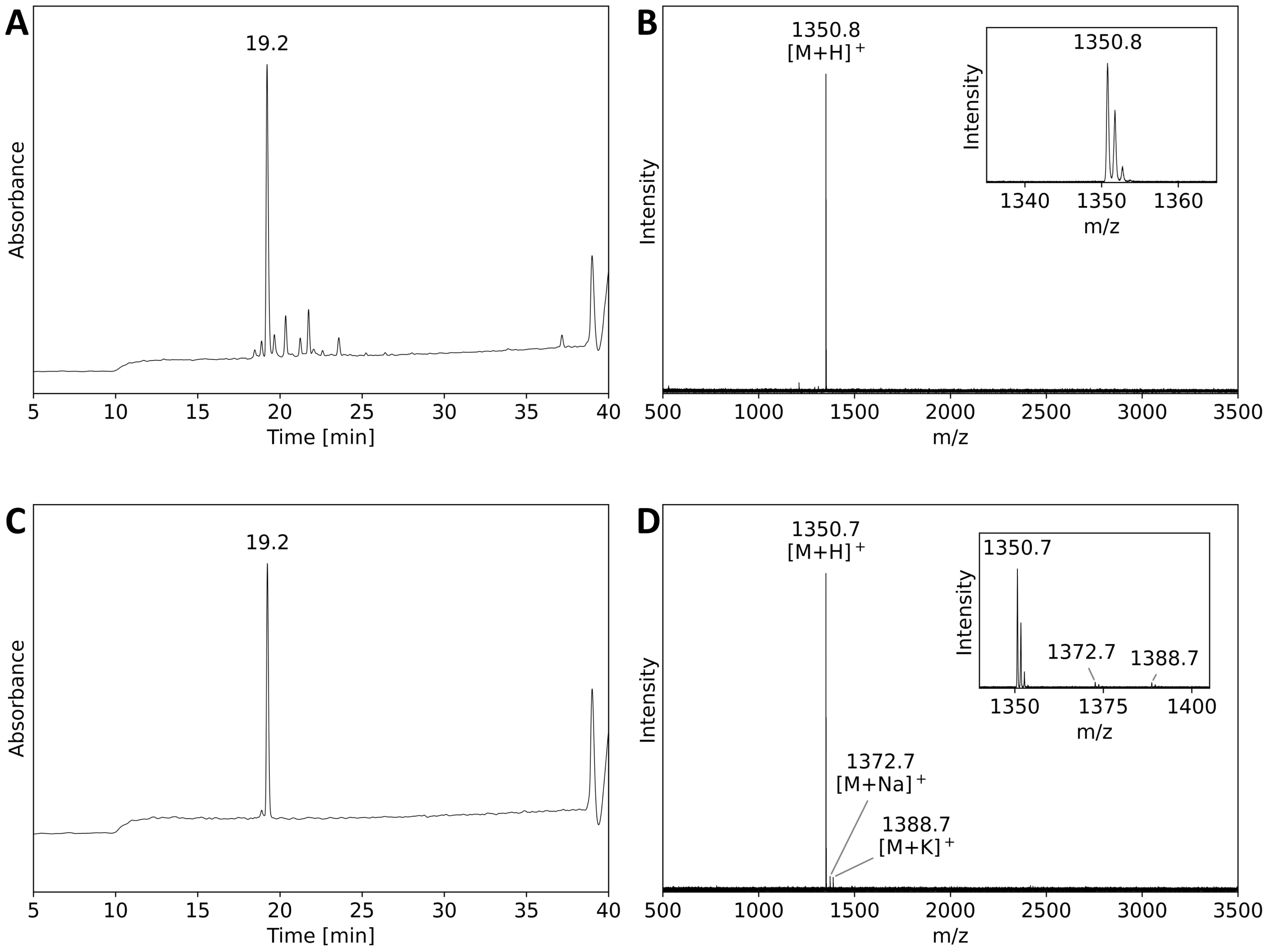

Figure S6: **P1-N3** A) Analytical HPLC (220 nm) of crude peptide (from **P1-OH** Batch 1). B) MALDI-TOF MS of crude peptide (from **P1-OH** Batch 1). C) Analytical HPLC (220 nm) of purified peptide. D) MALDI-TOF MS of purified peptide. Calculated mass: $[M+H]^+$ 1350.8, $[M+Na]^+$ 1372.8, $[M+K]^+$ 1388.8.

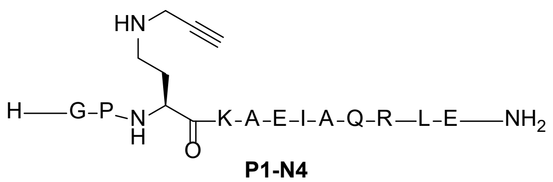

P1-N4
Chemical Formula: $C_{59}H_{101}N_{19}O_{17}$
Exact Mass: 1347.76
Molecular Weight: 1348.57

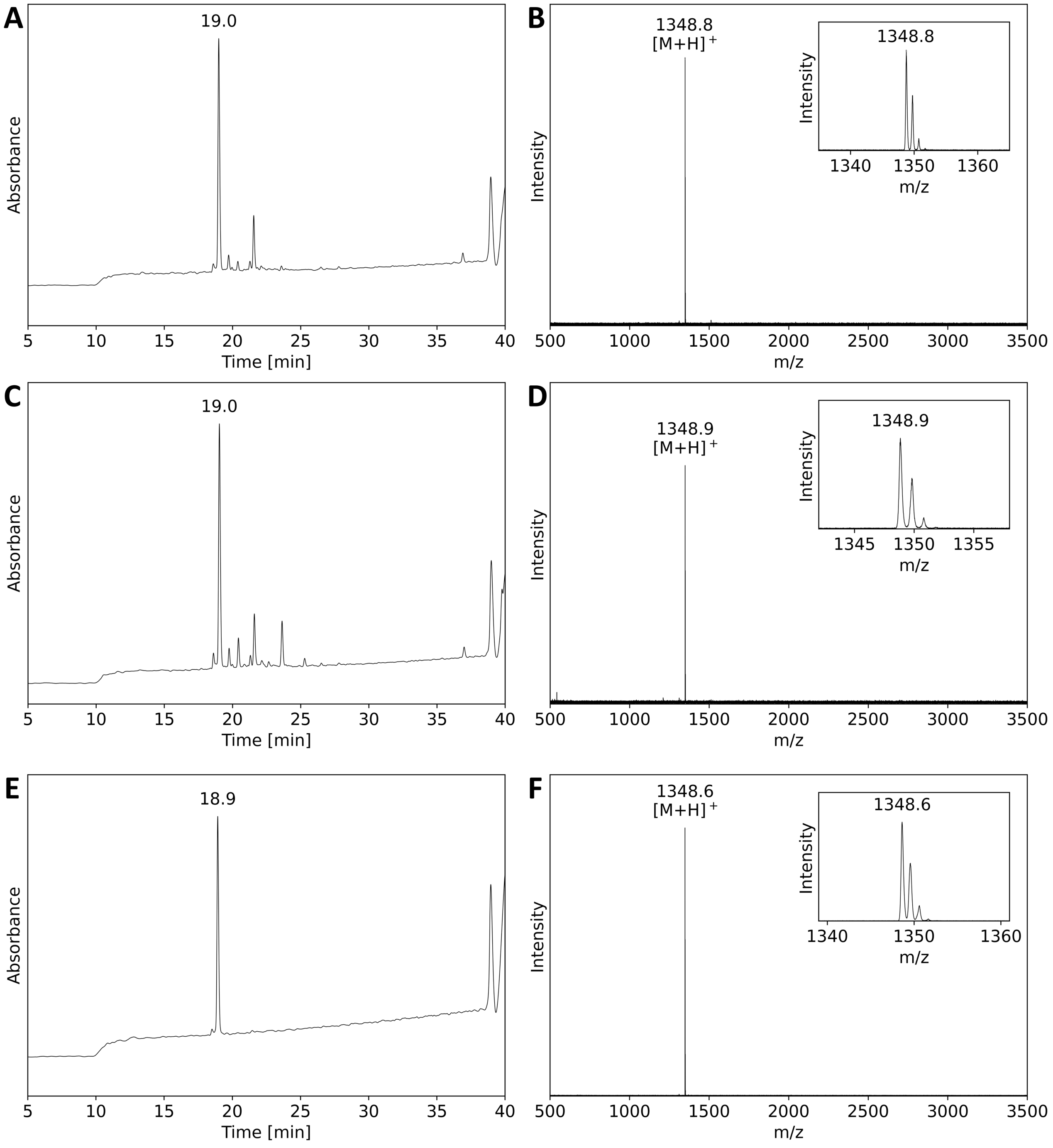

Figure 7: **P1-N4** A) Analytical HPLC (220 nm) of crude peptide (from **P1-OH** Batch 1; 2.5 M solution of **N4**). B) MALDI-TOF MS of crude peptide (from **P1-OH** Batch 1; 2.5 M solution of **N4**). C) Analytical HPLC (220 nm) of crude peptide (from **P1-OH** Batch 1; 1.0 M solution of **N4**). D) MALDI-TOF MS of crude peptide (from **P1-OH** Batch 1; 1.0 M solution of **N4**). E) Analytical HPLC (220 nm) of purified peptide. F) MALDI-TOF MS of purified peptide. Calculated mass: $[M+H]^+$ 1348.8.

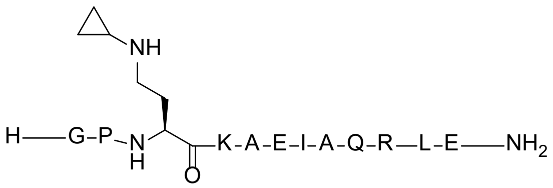

P1-N5

Chemical Formula: $C_{59}H_{103}N_{19}O_{17}$

Exact Mass: 1349.78

Molecular Weight: 1350.59

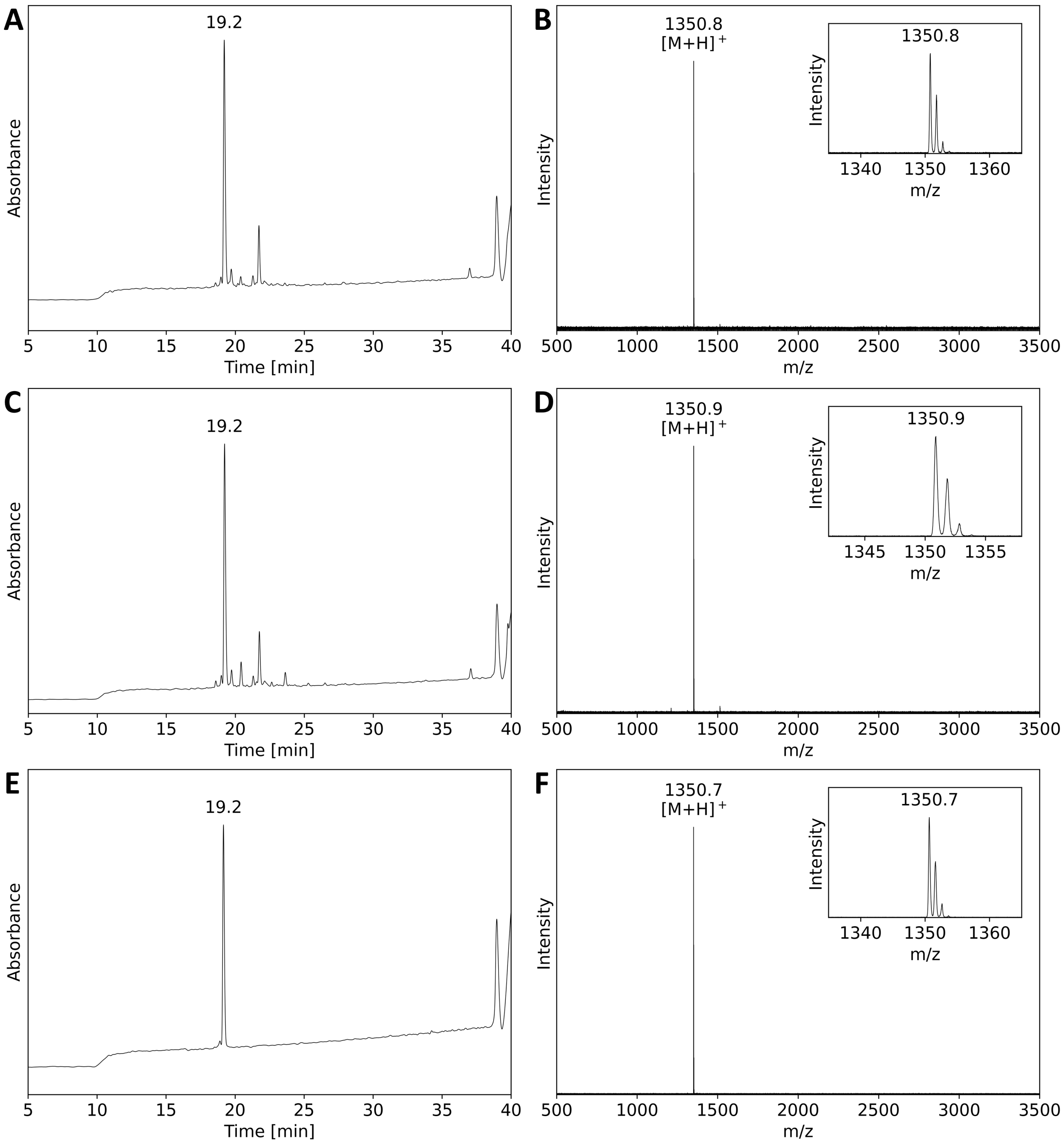

Figure S8: **P1-N5** A) Analytical HPLC (220 nm) of crude peptide (from **P1-OH** Batch 1; 2.5 M solution of **N5**). B) MALDI-TOF MS of crude peptide (from **P1-OH** Batch 1; 2.5 M solution of **N5**). C) Analytical HPLC (220 nm) of crude peptide (from **P1-OH** Batch 1; 1.0 M solution of **N5**). D) MALDI-TOF MS of crude peptide (from **P1-OH** Batch 1; 1.0 M solution of **N5**). E) Analytical HPLC (220 nm) of purified peptide. F) MALDI-TOF MS of purified peptide. Calculated mass: $[M+H]^+$ 1350.8.

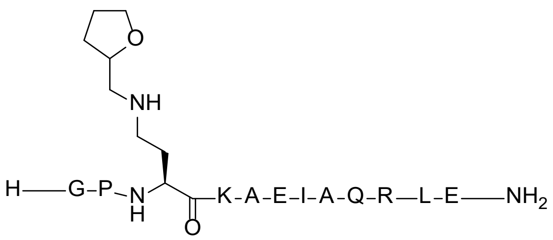

P1-N6

Chemical Formula: $C_{61}H_{107}N_{19}O_{18}$
Exact Mass: 1393.80
Molecular Weight: 1394.64

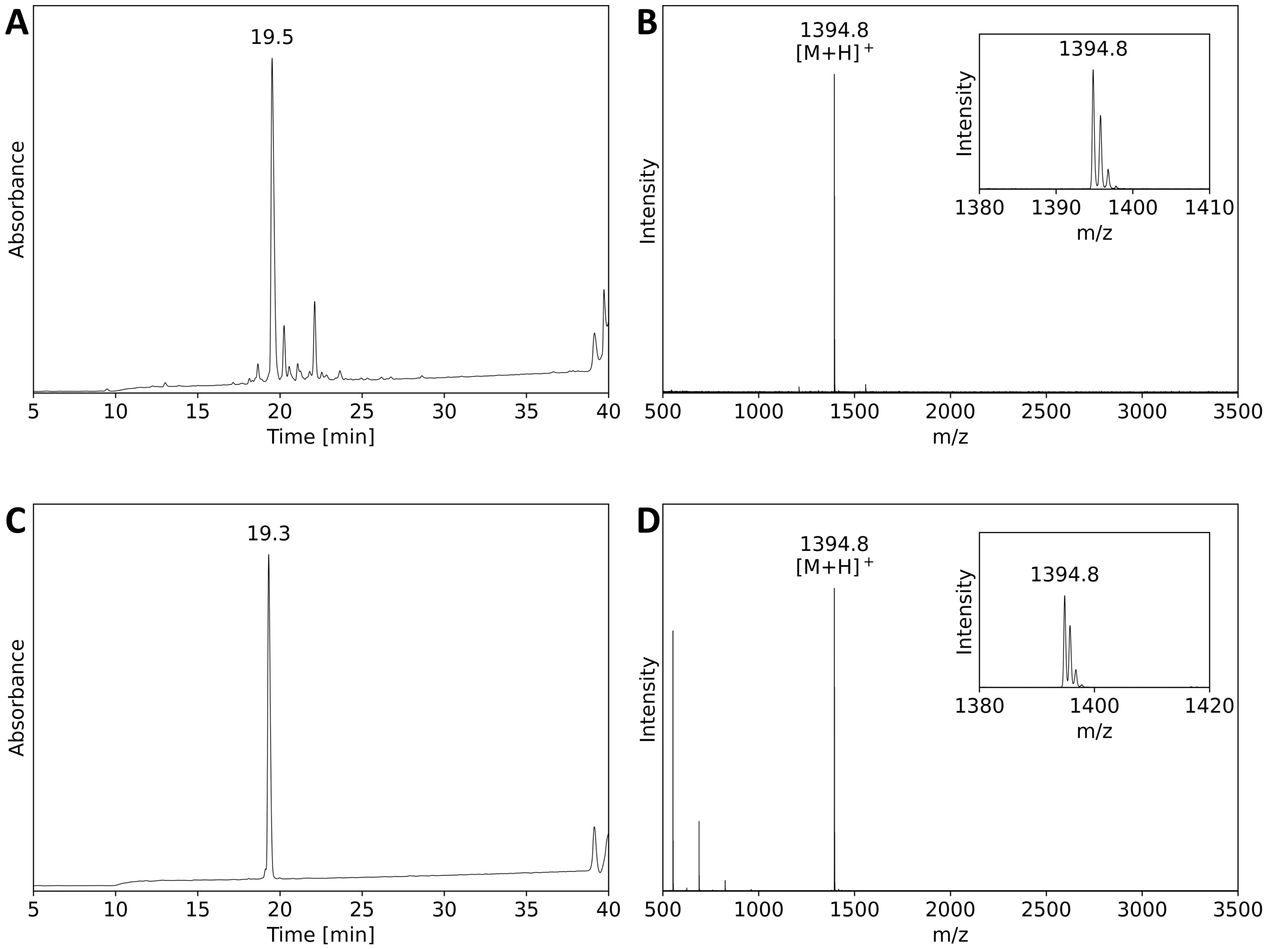

Figure S9: **P1-N6** A) Analytical HPLC (220 nm) of crude peptide (from **P1-OH** Batch 1). B) MALDI-TOF MS of crude peptide (from **P1-OH** Batch 1). C) Analytical HPLC (220 nm) of purified peptide. D) MALDI-TOF MS of purified peptide. Calculated mass: $[M+H]^+$ 1394.8.

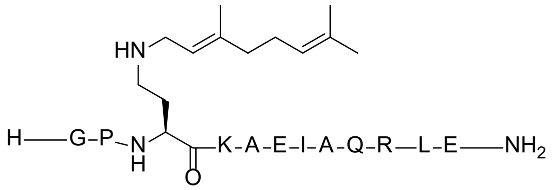

P1-N7

Chemical Formula: $C_{66}H_{115}N_{19}O_{17}$

Exact Mass: 1445.87

Molecular Weight: 1446.76

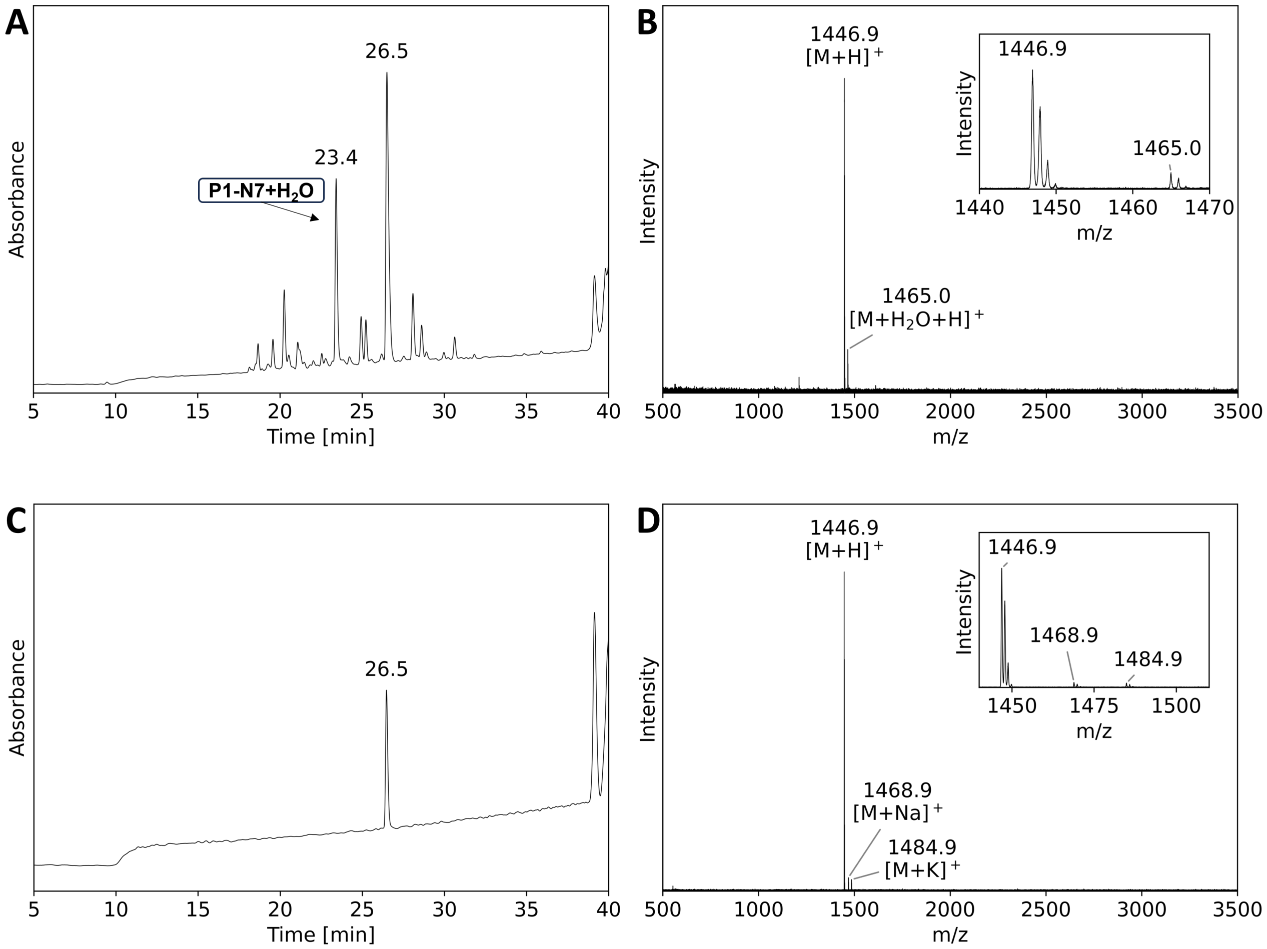

Figure S10: **P1-N7** A) Analytical HPLC (220 nm) of crude peptide (from **P1-OH** Batch 1). B) MALDI-TOF MS of crude peptide (from **P1-OH** Batch 1). C) Analytical HPLC (220 nm) of purified peptide. D) MALDI-TOF MS of purified peptide. Calculated mass: $[M+H]^+$ 1446.9, $[M+Na]^+$ 1468.9, $[M+K]^+$ 1484.8, $[M+H_2O+H]^+$ 1464.9.

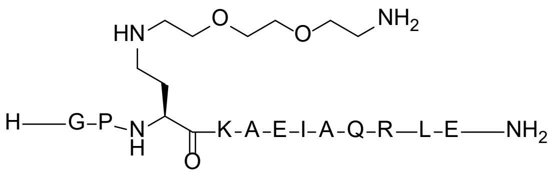

P1-N8

Chemical Formula: $C_{62}H_{112}N_{20}O_{19}$

Exact Mass: 1440.84

Molecular Weight: 1441.70

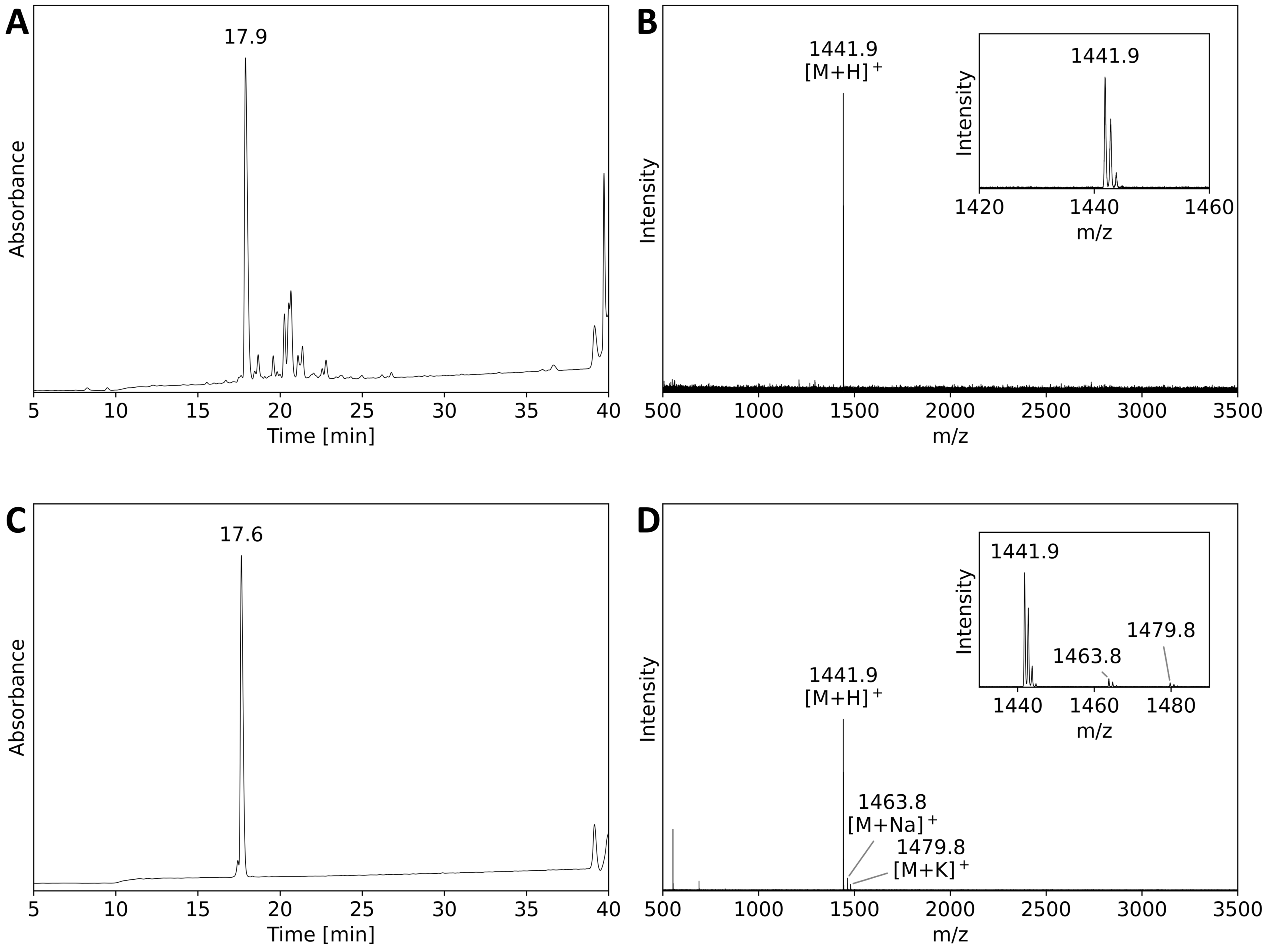

Figure S11: **P1-N8** A) Analytical HPLC (220 nm) of crude peptide (from **P1-OH** Batch 1). B) MALDI-TOF MS of crude peptide (from **P1-OH** Batch 1). C) Analytical HPLC (220 nm) of purified peptide. D) MALDI-TOF MS of purified peptide. Calculated mass: $[M+H]^+$ 1441.9, $[M+Na]^+$ 1463.8, $[M+K]^+$ 1479.8.

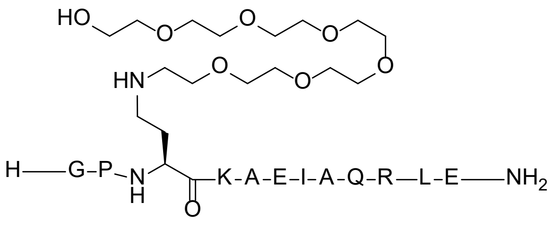

P1-N9

Chemical Formula: $C_{70}H_{127}N_{19}O_{24}$
Exact Mass: 1617.93
Molecular Weight: 1618.90

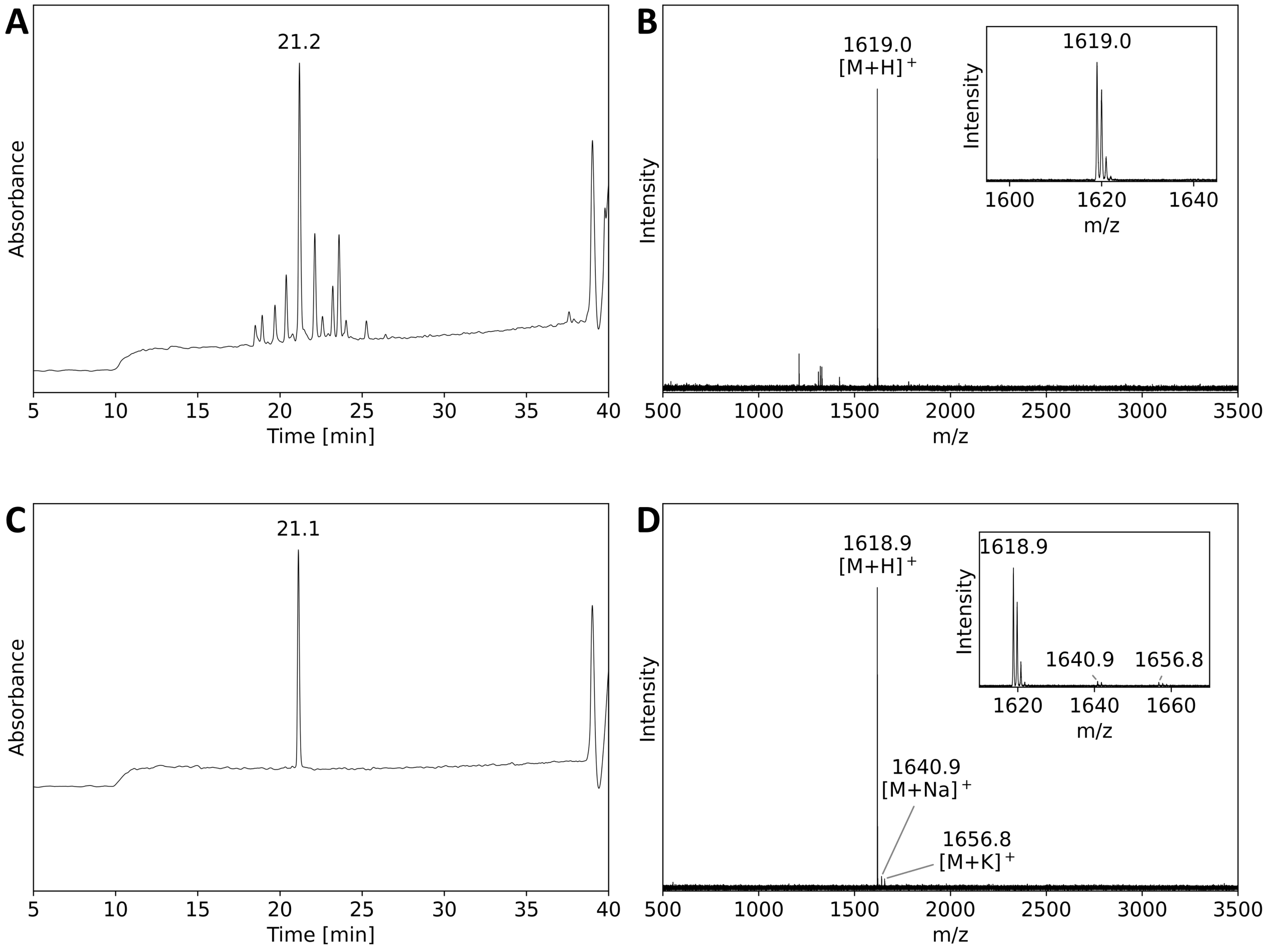

Figure S12: **P1-N9** A) Analytical HPLC (220 nm) of crude peptide (from **P1-OH** Batch 1). B) MALDI-TOF MS of crude peptide (from **P1-OH** Batch 1). C) Analytical HPLC (220 nm) of purified peptide. D) MALDI-TOF MS of purified peptide. Calculated mass: $[M+H]^+$ 1618.9, $[M+Na]^+$ 1640.9, $[M+K]^+$ 1656.9.

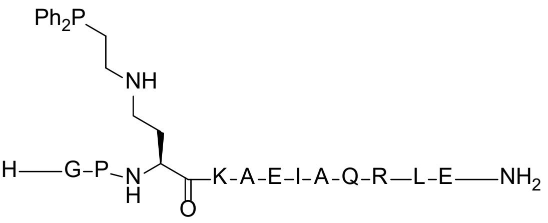

P1-N10

Chemical Formula: $C_{70}H_{112}N_{19}O_{17}P$
Exact Mass: 1521.82
Molecular Weight: 1522.76

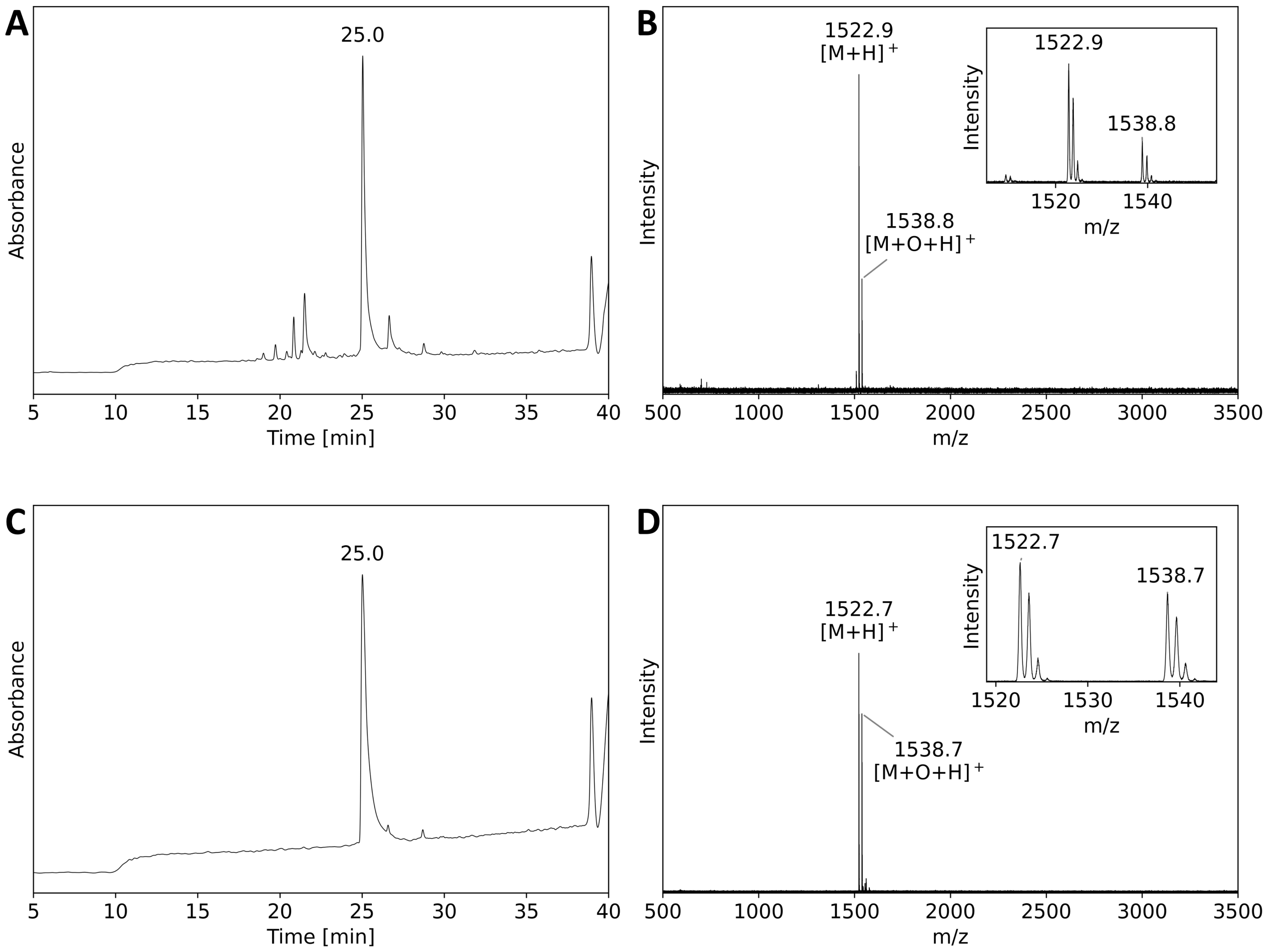

Figure S13: **P1-N10** A) Analytical HPLC (220 nm) of crude peptide (from **P1-OH** Batch 1). B) MALDI-TOF MS of crude peptide (from **P1-OH** Batch 1). C) Analytical HPLC (220 nm) of purified peptide. D) MALDI-TOF MS of purified peptide. Calculated mass: $[M+H]^+$ 1522.8, $[M+O+H]^+$ 1538.8.

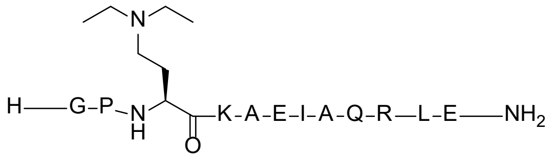

P1-N11

Chemical Formula: $C_{60}H_{107}N_{19}O_{17}$
Exact Mass: 1365.81
Molecular Weight: 1366.63

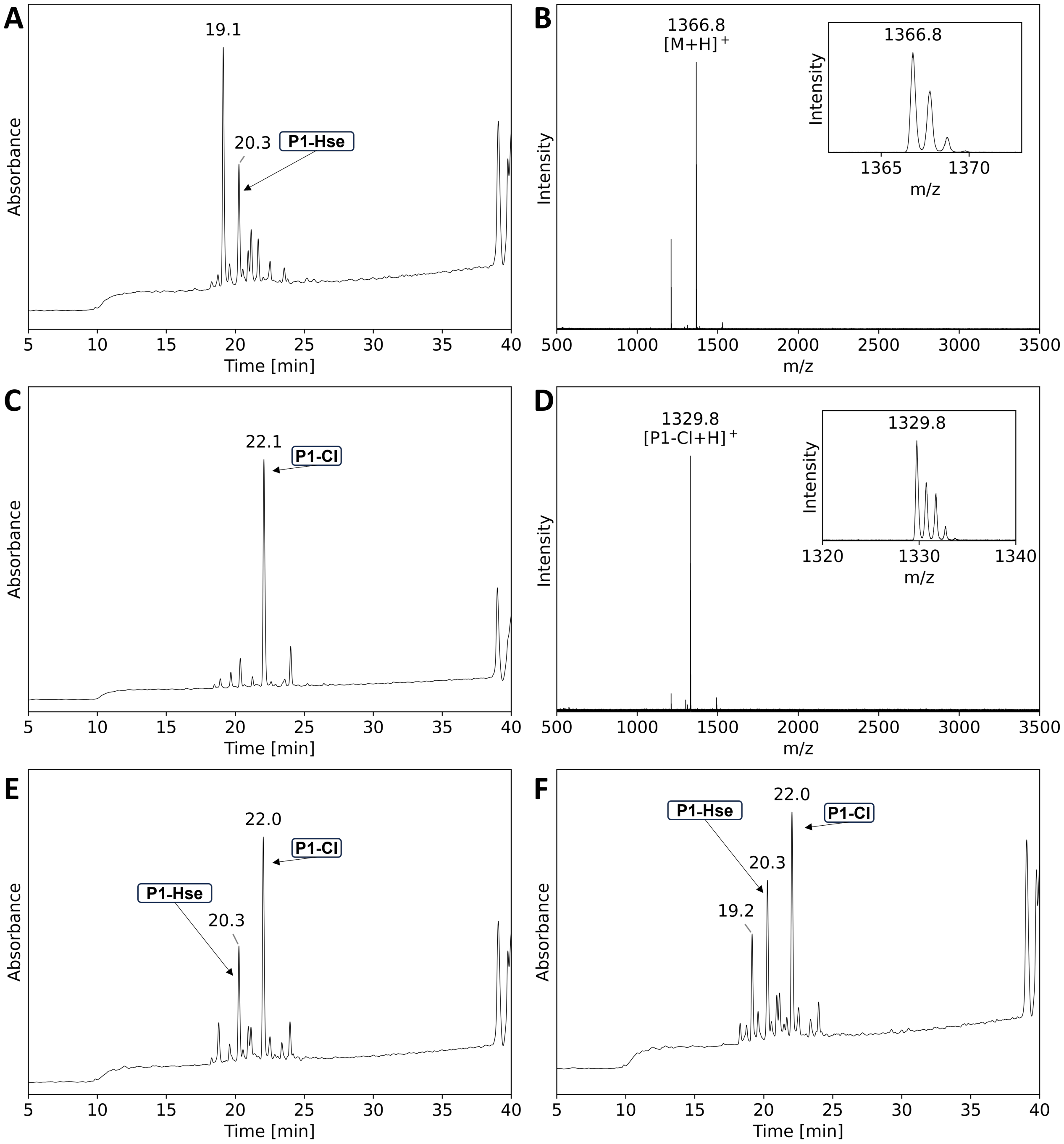

Figure S14: **P1-N11** A) Analytical HPLC (220 nm) ofcrude peptide (from **P1-OH** Batch 2 with **N11** according to standard conditions). B) MALDI-TOF MS of crude peptide (from **P1-OH** Batch 2 with **N11**). C) Analytical HPLC (220 nm) of crude peptide (from **P1-OH** Batch 1 with **N11-HCl** in DMF). D) MALDI-TOF MS of crude peptide (from **P1-OH** Batch 1 with **N11-HCl** in DMF). E) Analytical HPLC (220 nm) of crude peptide (from **P1-OH** Batch 2 with **N11-HCl** in DMF, additive: 1.0 M pyridine). F) Analytical HPLC (220 nm) of crude peptide (from **P1-OH** Batch 2 with **N11-HCl** in DMF; additive: 1.0 M DIPEA). Calculated mass: $[M+H]^+$ 1366.8, $[P1\text{-}Cl+H]^+$ 1329.7.

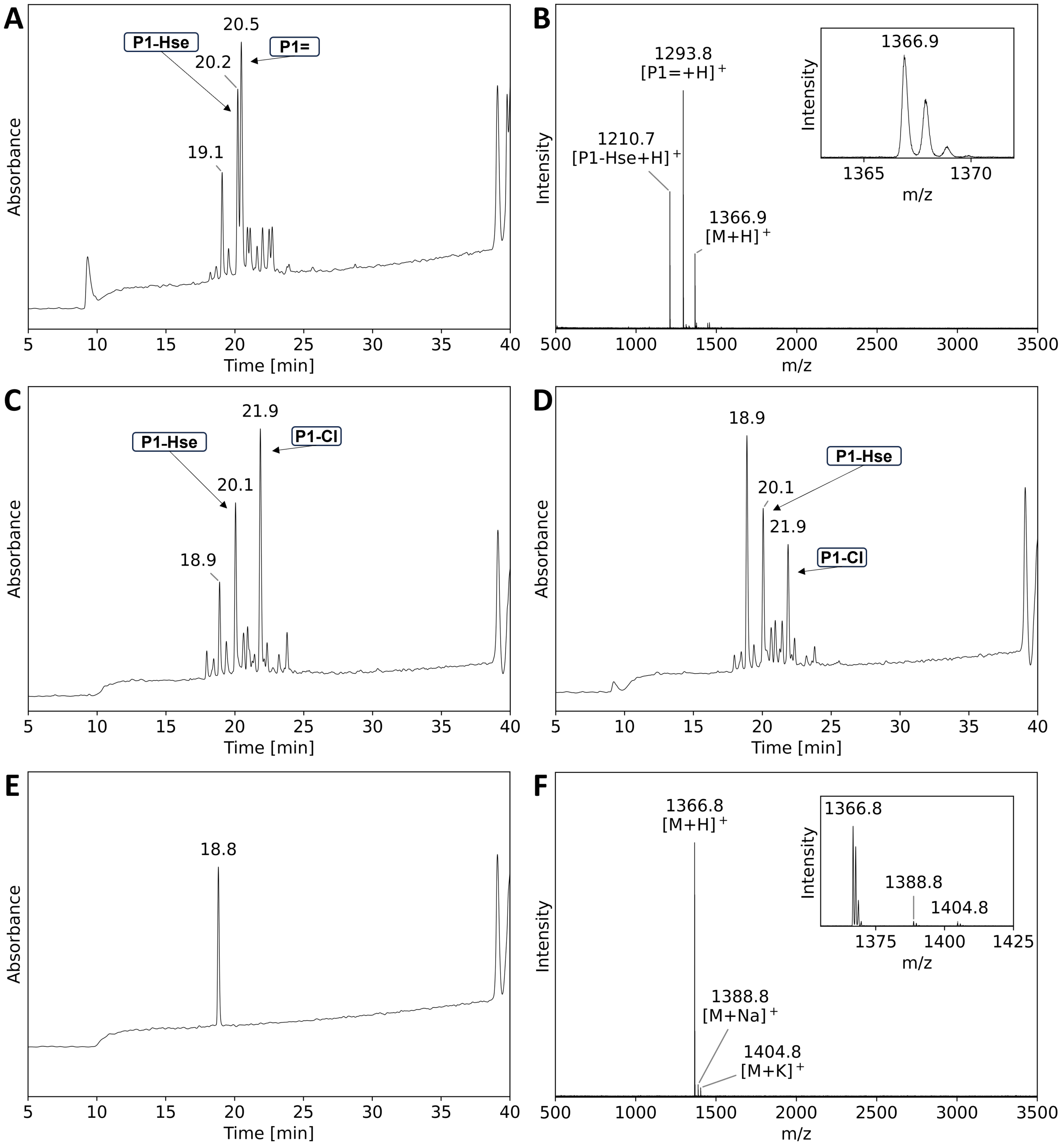

Figure S15: **P1-N11** A) Analytical HPLC (220 nm) of crude peptide (from **P1-OH** Batch 2 with **N11-HCl** in DMF; additive: 1.0 M DBU). B) MALDI-TOF MS of crude peptide (from **P1-OH** Batch 2 with **N11-HCl** in DMF; additive: 1.0 M DBU). C) Analytical HPLC (220 nm) of crude peptide (from **P1-OH** Batch 2 with **N11-HCl** in DMF; additive: 10 M. DIPEA). D) Analytical HPLC (220 nm) of crude peptide (from **P1-OH** Batch 2 with **N11-HCl** in DMF; additive: 140 mM DBU). E) Analytical HPLC (220 nm) of purified peptide. F) MALDI-TOF MS of purified peptide. Calculated mass: $[P1\text{-}Hse+H]^+$ 1210.7, $[P1{=}+H]^+$ 1293.7, $[M+H]^+$ 1366.8, $[M+Na]^+$ 1388.8, $[M+K]^+$ 1404.8.

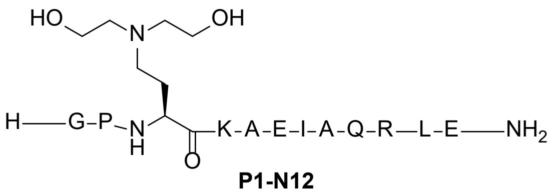

P1-N12

Chemical Formula: $C_{60}H_{107}N_{19}O_{19}$

Exact Mass: 1397.80

Molecular Weight: 1398.63

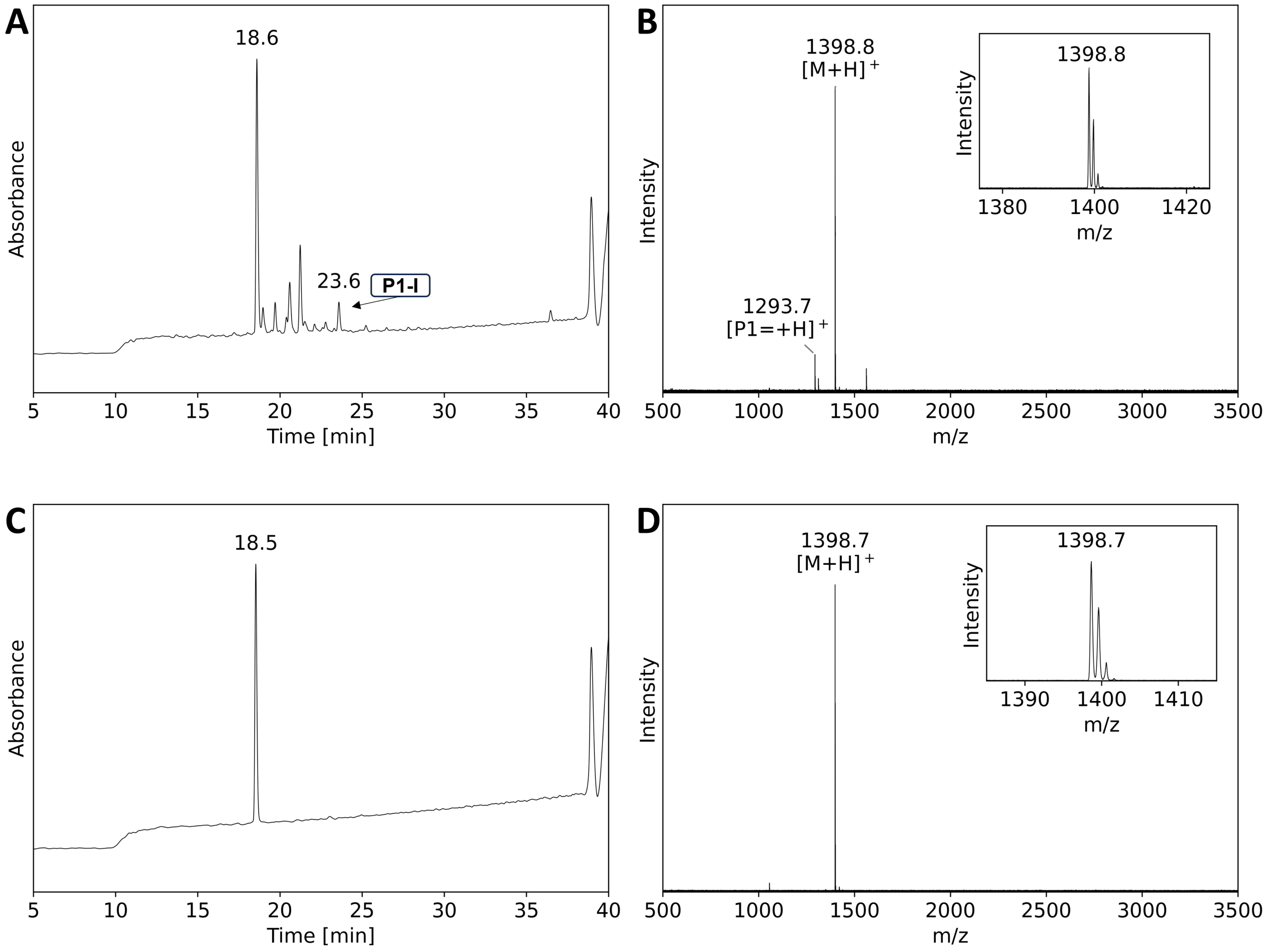

Figure S16: **P1-N12** A) Analytical HPLC (220 nm) of crude peptide (from **P1-OH** Batch 1). B) MALDI-TOF MS of crude peptide (from **P1-OH** Batch 1). C) Analytical HPLC (220 nm) of purified peptide. D) MALDI-TOF MS of purified peptide. Calculated mass: $[P1{=}{+}H]^+$ 1293.7, $[M+H]^+$ 1398.8.

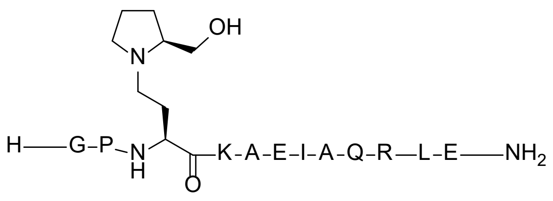

P1-N13

Chemical Formula: $C_{61}H_{107}N_{19}O_{18}$
Exact Mass: 1393.80
Molecular Weight: 1394.64

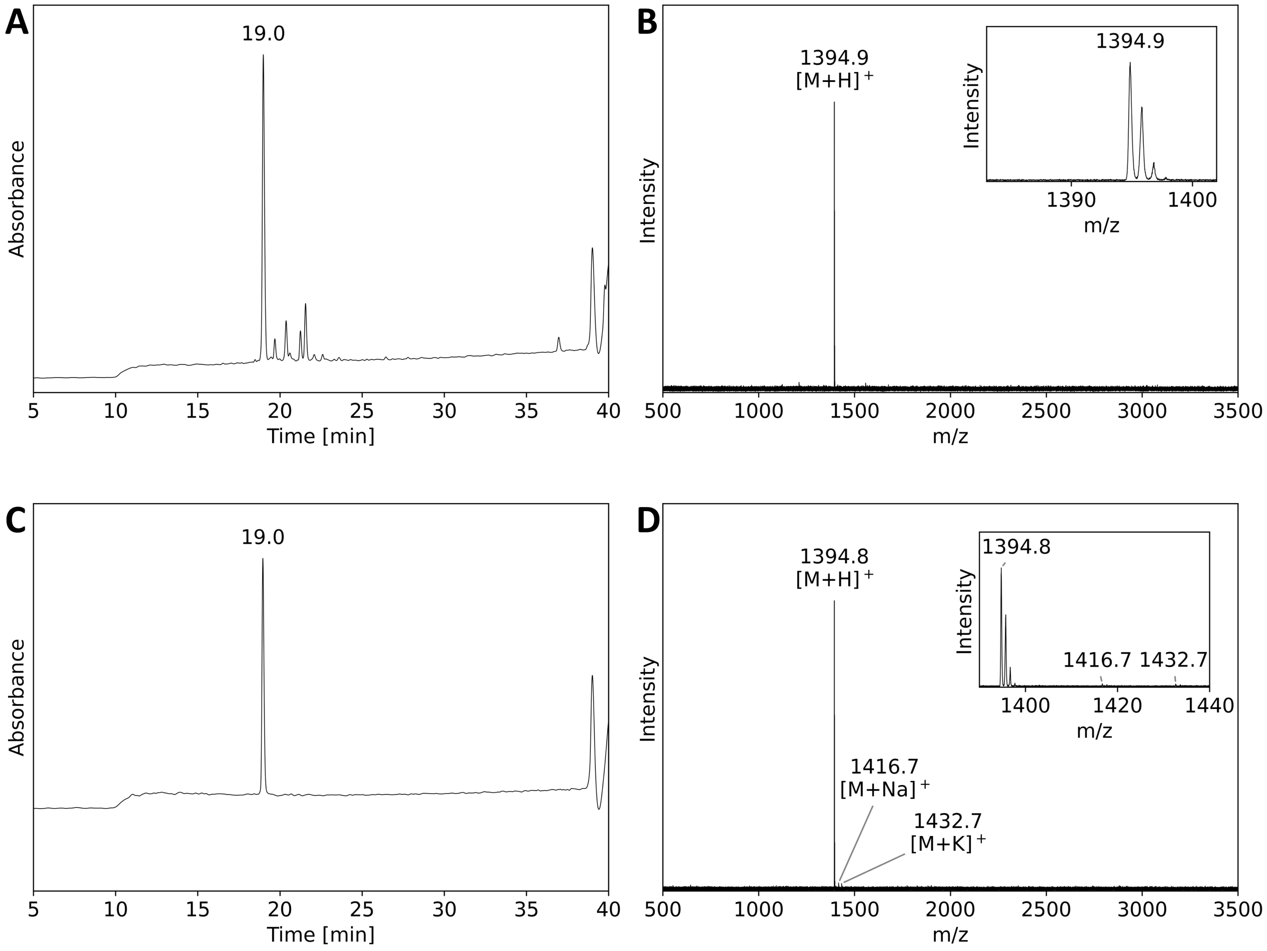

Figure S17: **P1-N13** A) Analytical HPLC (220 nm) of crude peptide (from **P1-OH** Batch 1). B) MALDI-TOF MS of crude peptide (from **P1-OH** Batch 1). C) Analytical HPLC (220 nm) of purified peptide. D) MALDI-TOF MS of purified peptide. Calculated mass: $[M+H]^+$ 1394.8, $[M+Na]^+$ 1416.8, $[M+K]^+$ 1432.8.

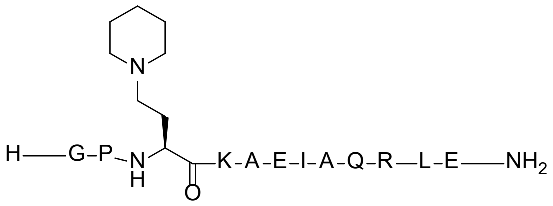

P1-N14
Chemical Formula: $C_{61}H_{107}N_{19}O_{17}$
Exact Mass: 1377.81
Molecular Weight: 1378.64

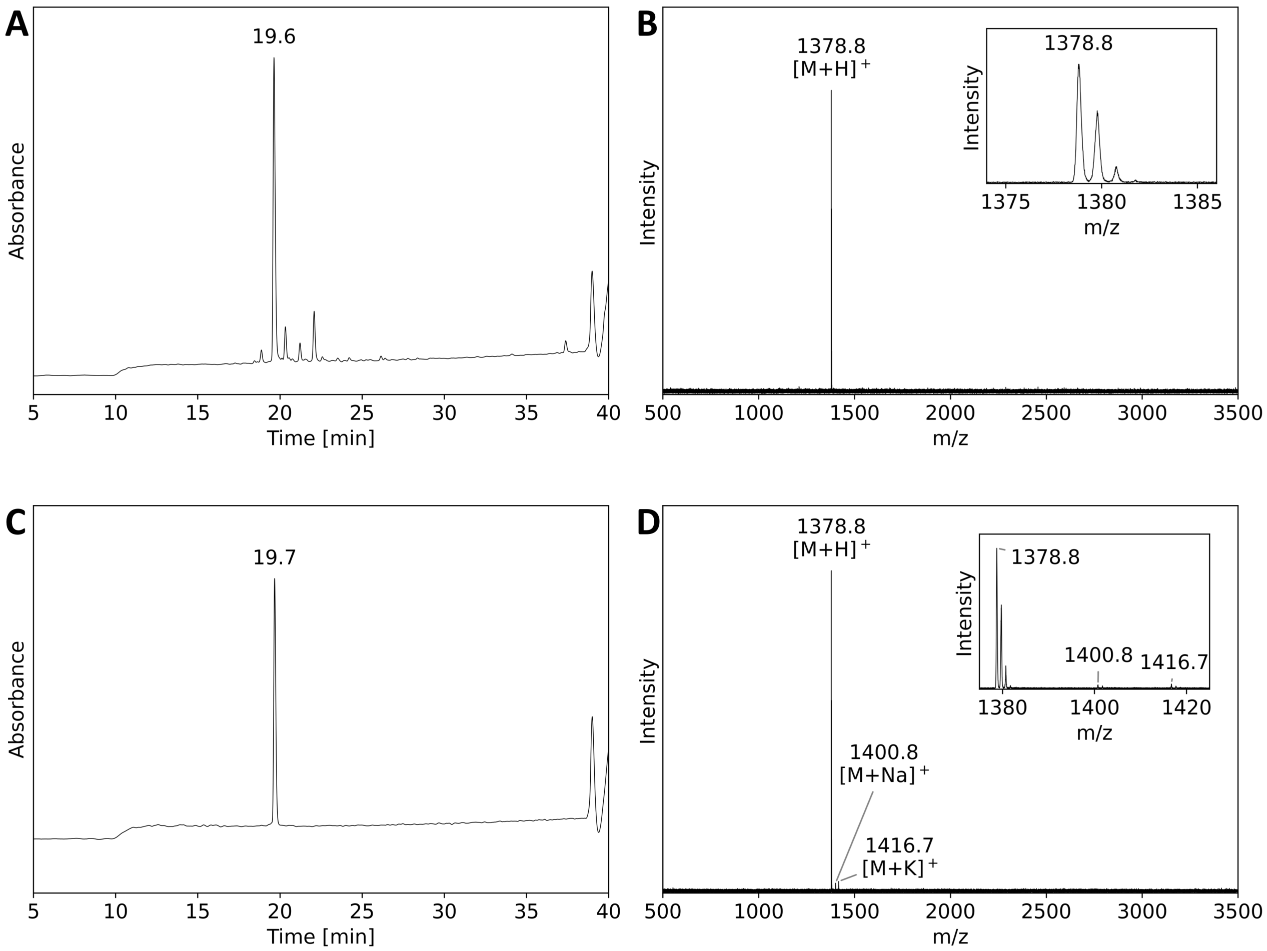

Figure S18: **P1-N14** A) Analytical HPLC (220 nm) of crude peptide (from **P1-OH** Batch 1). B) MALDI-TOF MS of crude peptide (from **P1-OH** Batch 1). C) Analytical HPLC (220 nm) of purified peptide. D) MALDI-TOF MS of purified peptide. Calculated mass: $[M+H]^+$ 1378.8, $[M+Na]^+$ 1400.8, $[M+K]^+$ 1416.8.

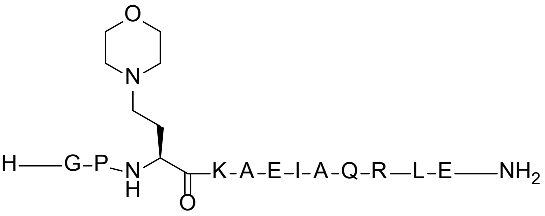

P1-N15

Chemical Formula: $C_{60}H_{105}N_{19}O_{18}$

Exact Mass: 1379.79

Molecular Weight: 1380.62

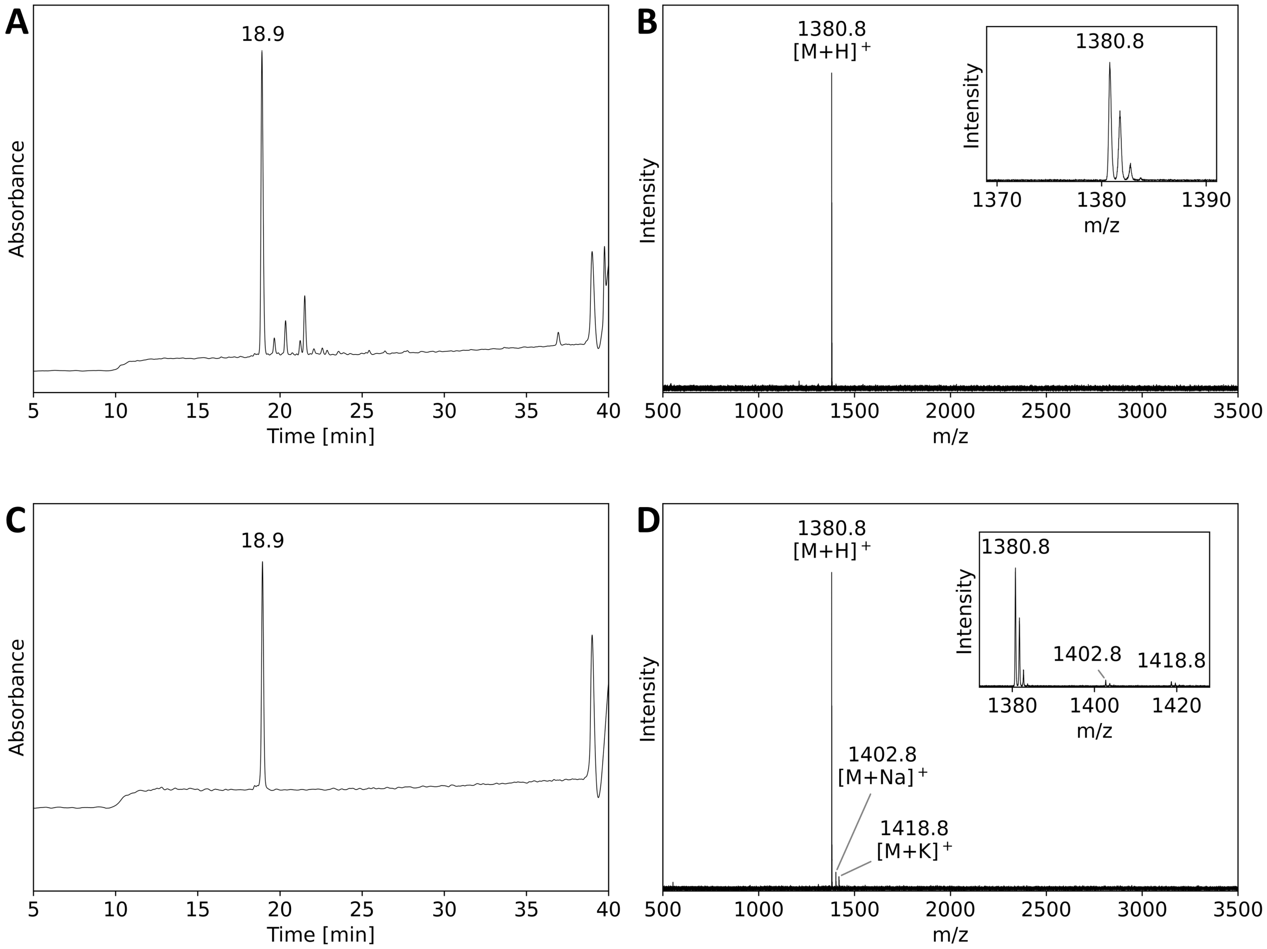

Figure S19: **P1-N15** A) Analytical HPLC (220 nm) of crude peptide (from **P1-OH** Batch 1). B) MALDI-TOF MS of crude peptide (from **P1-OH** Batch 1). C) Analytical HPLC (220 nm) of purified peptide. D) MALDI-TOF MS of purified peptide. Calculated mass: $[M+H]^+$ 1380.8, $[M+Na]^+$ 1402.8, $[M+K]^+$ 1418.8.

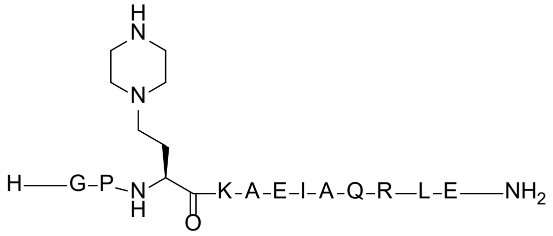

P1-N16
Chemical Formula: $C_{60}H_{106}N_{20}O_{17}$
Exact Mass: 1378.80
Molecular Weight: 1379.63

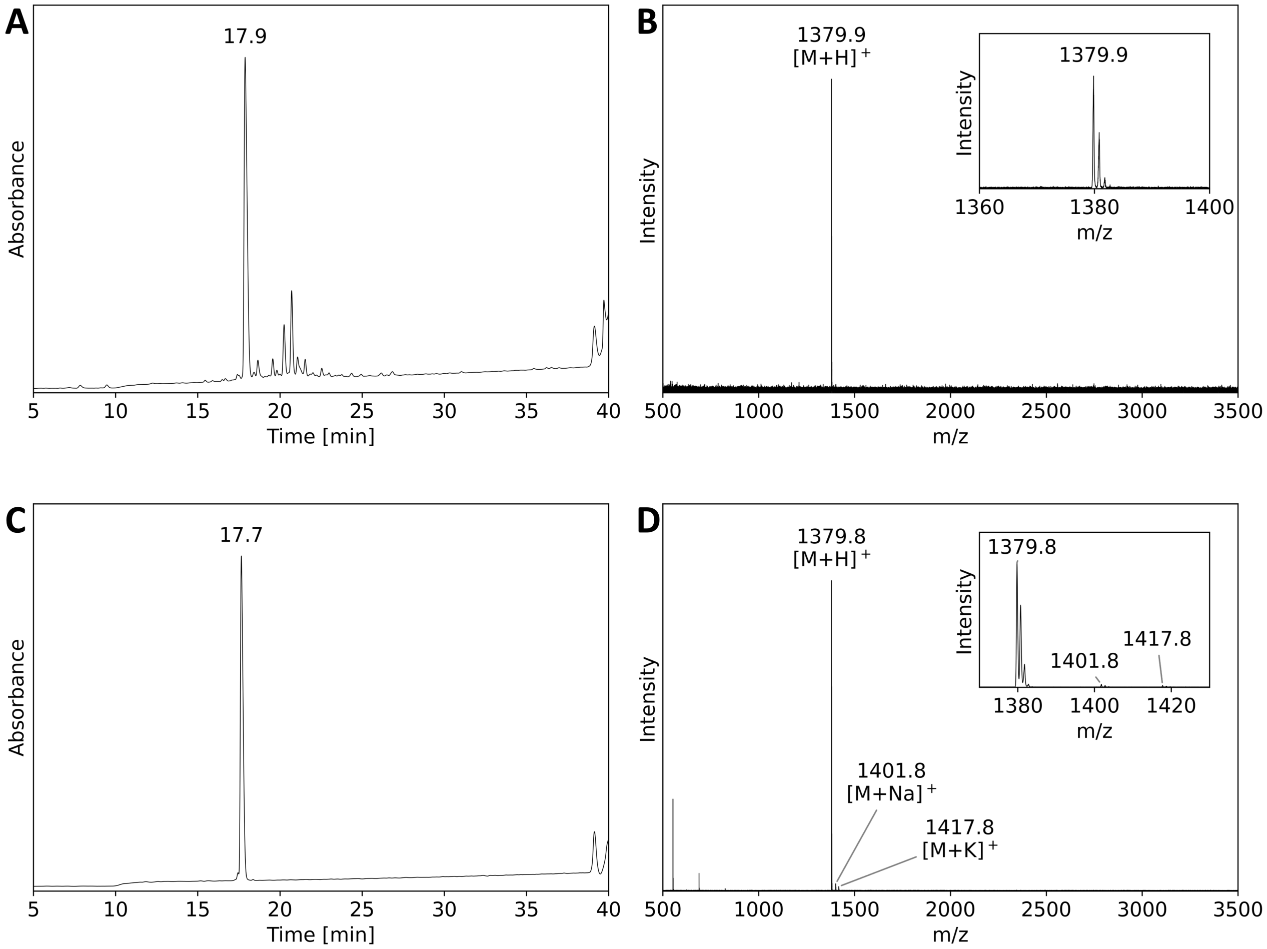

Figure S20: **P1-N16** A) Analytical HPLC (220 nm) of crude peptide (from **P1-OH** Batch 1). B) MALDI-TOF MS of crude peptide (from **P1-OH** Batch 1). C) Analytical HPLC (220 nm) of purified peptide. D) MALDI-TOF MS of purified peptide. Calculated mass: $[M+H]^+$ 1379.8, $[M+Na]^+$ 1401.8, $[M+K]^+$ 1417.8.

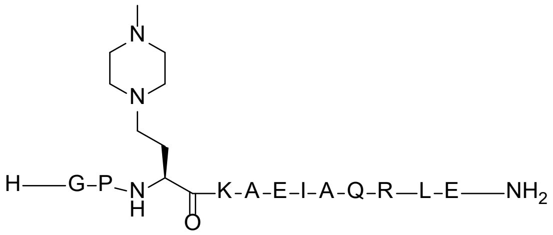

P1-N17
Chemical Formula: $C_{61}H_{108}N_{20}O_{17}$
Exact Mass: 1392.82
Molecular Weight: 1393.66

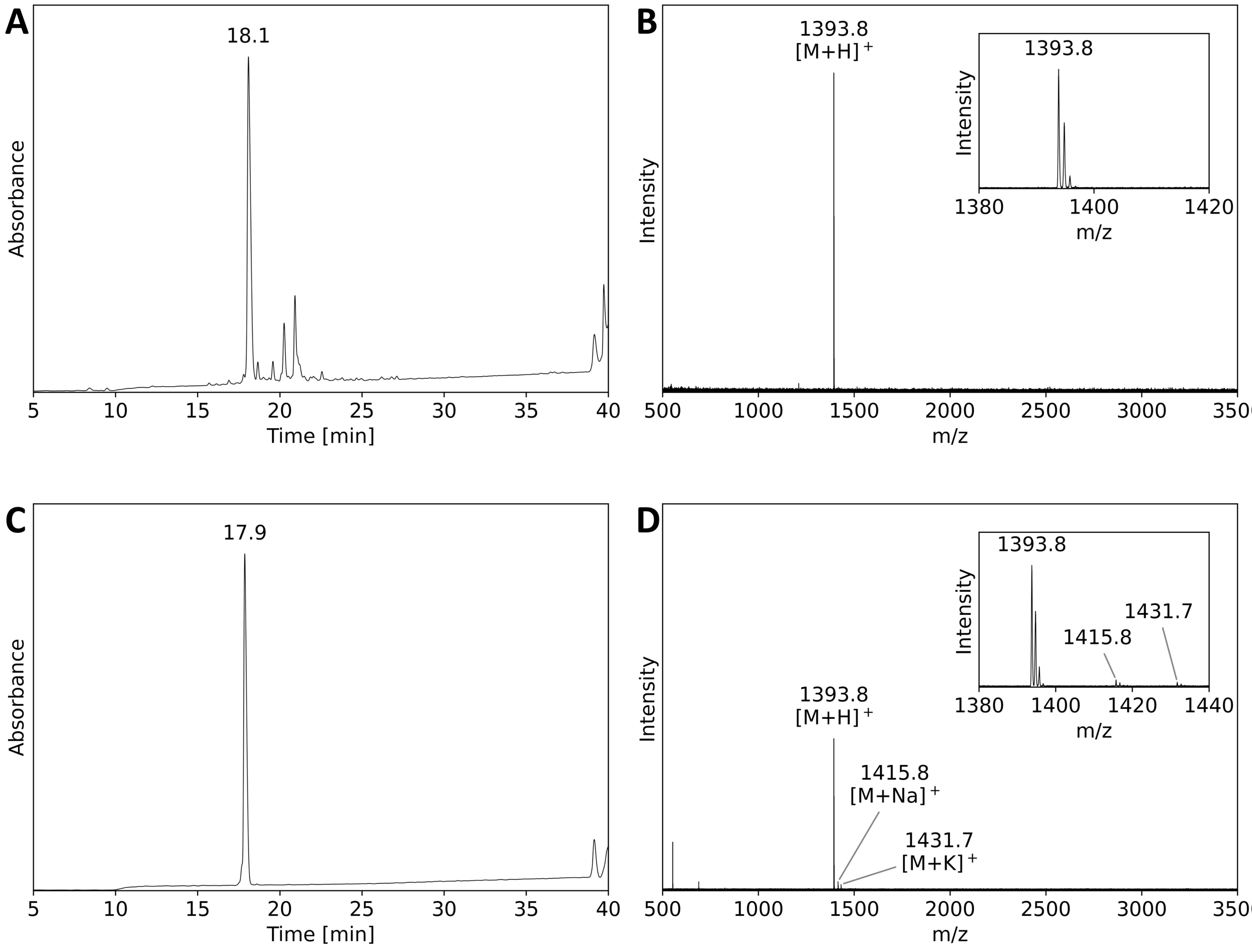

Figure S21: **P1-N17** A) Analytical HPLC (220 nm) of crude peptide (from **P1-OH** Batch 1). B) MALDI-TOF MS of crude peptide (from **P1-OH** Batch 1). C) Analytical HPLC (220 nm) of purified peptide. D) MALDI-TOF MS of purified peptide. Calculated mass: $[M+H]^+$ 1393.8, $[M+Na]^+$ 1415.8, $[M+K]^+$ 1431.8.

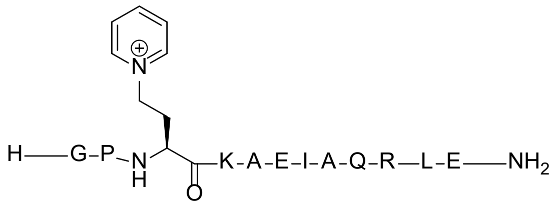

P1-N18

Chemical Formula: $C_{61}H_{102}N_{19}O_{17}^{+}$
Exact Mass: 1372.77
Molecular Weight: 1373.60

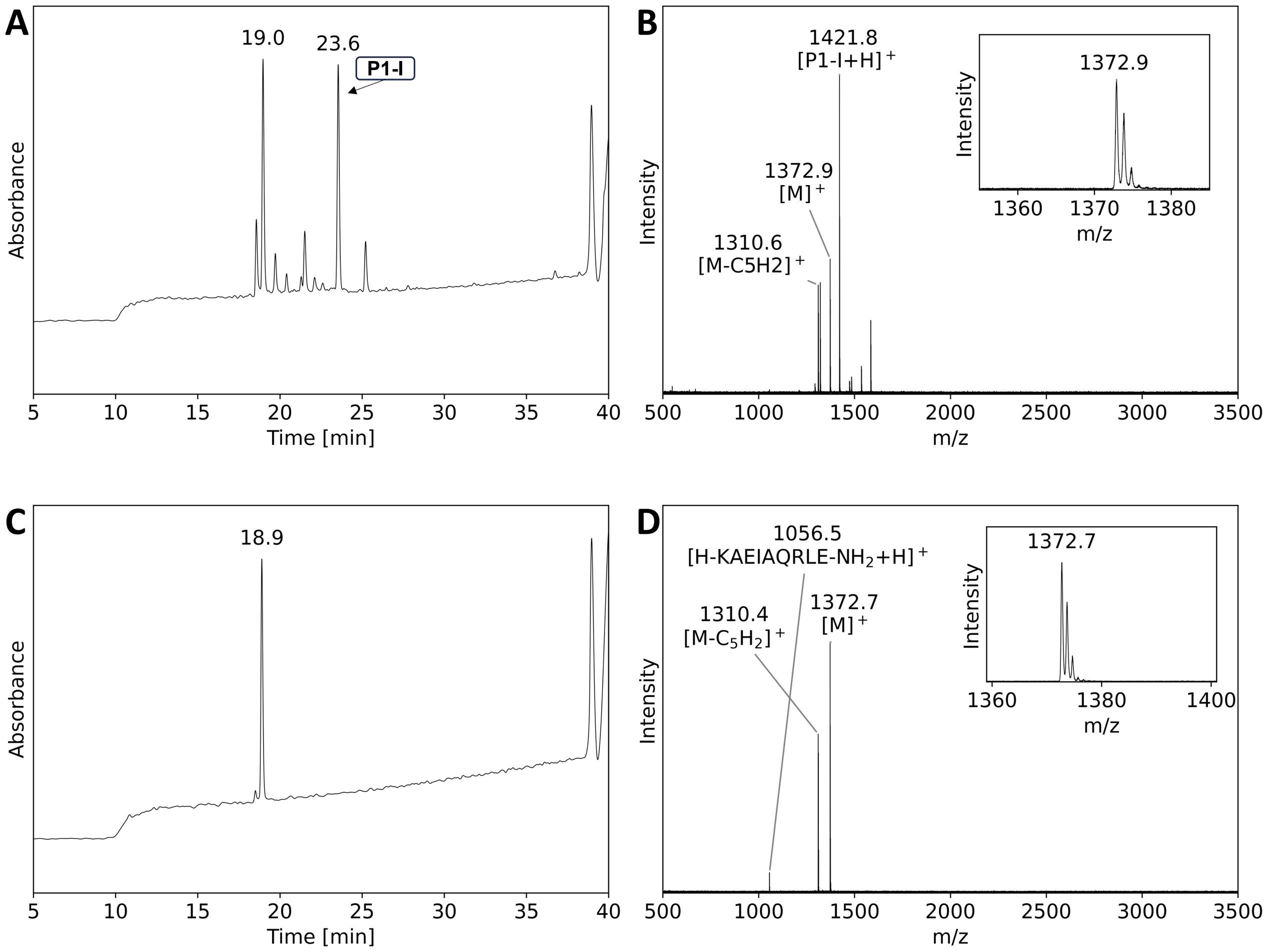

Figure S22: **P1-N18** A) Analytical HPLC (220 nm) of crude peptide (from **P1-OH** Batch 1; 2.5 M solution of **N18**). B) MALDI-TOF MS of crude peptide (from **P1-OH** Batch 1; 2.5 M solution of **N18**). C) Analytical HPLC (220 nm) of purified peptide. D) MALDI-TOF MS of purified peptide. Fragmentation of the pyridinium was observed in MALDI-TOF MS. Calculated mass: $[H\text{-}KAEIAQRLE\text{-}NH_2+H]^+$ 1056.6, $[M\text{-}C_5H_2]^+$ 1310.8, $[M]^+$ 1372.8, $[P1\text{-}I+H]^+$ 1421.6.

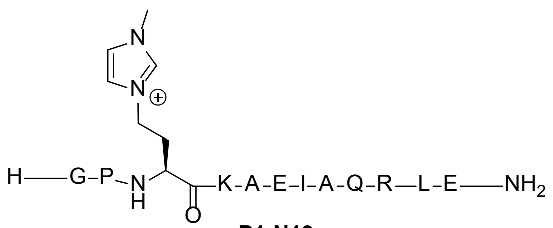

P1-N19

Chemical Formula: $C_{60}H_{103}N_{20}O_{17}^{+}$

Exact Mass: 1375.78

Molecular Weight: 1376.61

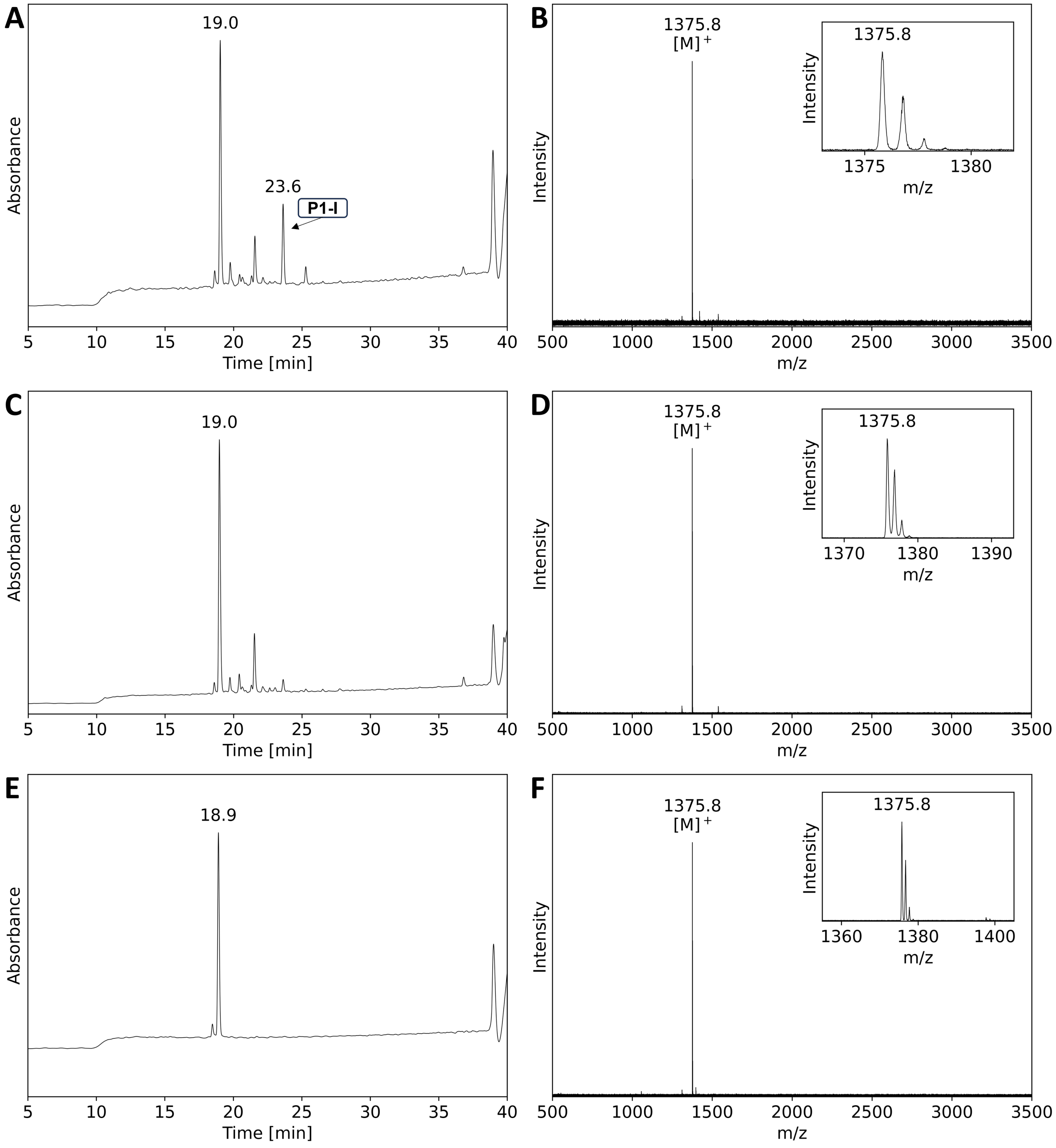

Figure S23: **P1-N19** A) Analytical HPLC (220 nm) of crude peptide (from **P1-OH** Batch 1; 2.5 M solution of **N19**). B) MALDI-TOF MS of crude peptide (from **P1-OH** Batch 1; 2.5 M solution of **N19**). C) Analytical HPLC (220 nm) of crude peptide (from **P1-OH** Batch 1; 2.5 M solution of **N19**, 64 h reaction time). D) MALDI-TOF MS of crude peptide (from **P1-OH** Batch 1; 2.5 M solution of **N19**, 64 h reaction time). E) Analytical HPLC (220 nm) of purified peptide. F) MALDI-TOF MS of purified peptide. Calculated mass: $[M]^{+}$ 1375.8.

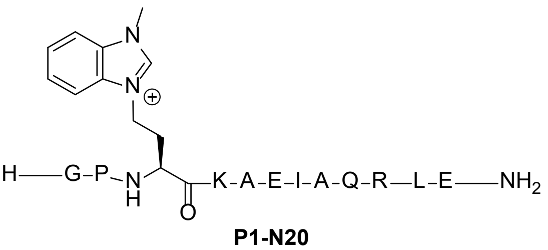

P1-N20

Chemical Formula: $C_{64}H_{105}N_{20}O_{17}^{+}$

Exact Mass: 1425.80

Molecular Weight: 1426.67

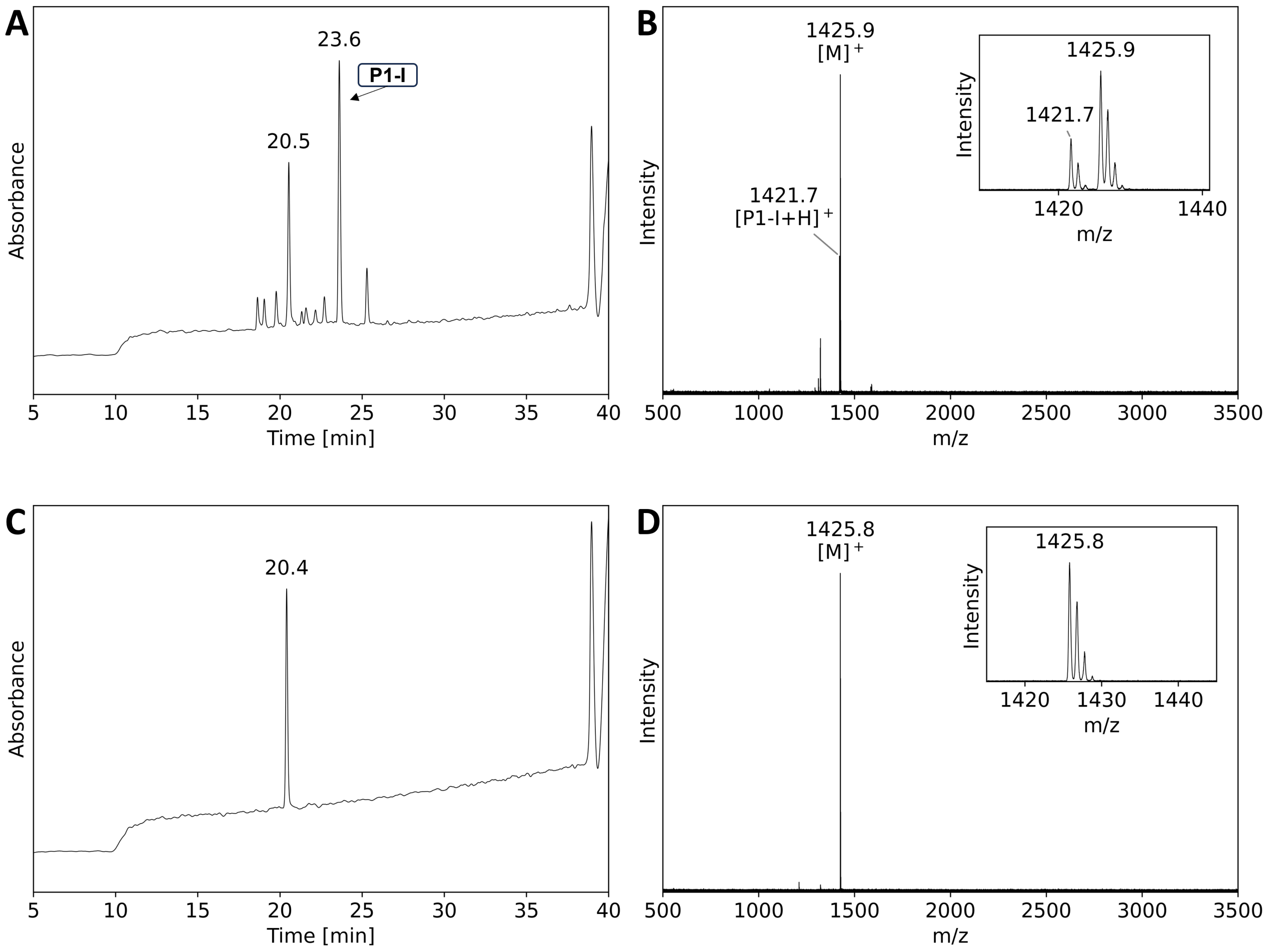

Figure S24: **P1-N20** A) Analytical HPLC (220 nm) of crude peptide (from **P1-OH** Batch 1; a 2.5 M solution of **N20**). B) MALDI-TOF MS of crude peptide (from **P1-OH** Batch 1; 2.5 M solution of **N20**). C) Analytical HPLC (220 nm) of purified peptide. D) MALDI-TOF MS of purified peptide. Calculated mass: $[M]^{+}$ 1425.8, $[P1\text{-}I+H]^{+}$ 1421.6.

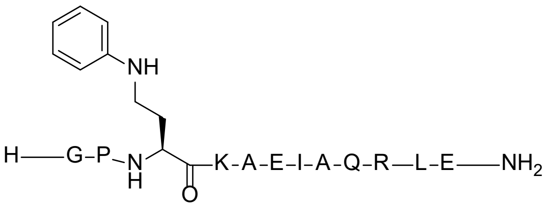

P1-N21
Chemical Formula: $C_{62}H_{103}N_{19}O_{17}$
Exact Mass: 1385.78
Molecular Weight: 1386.62

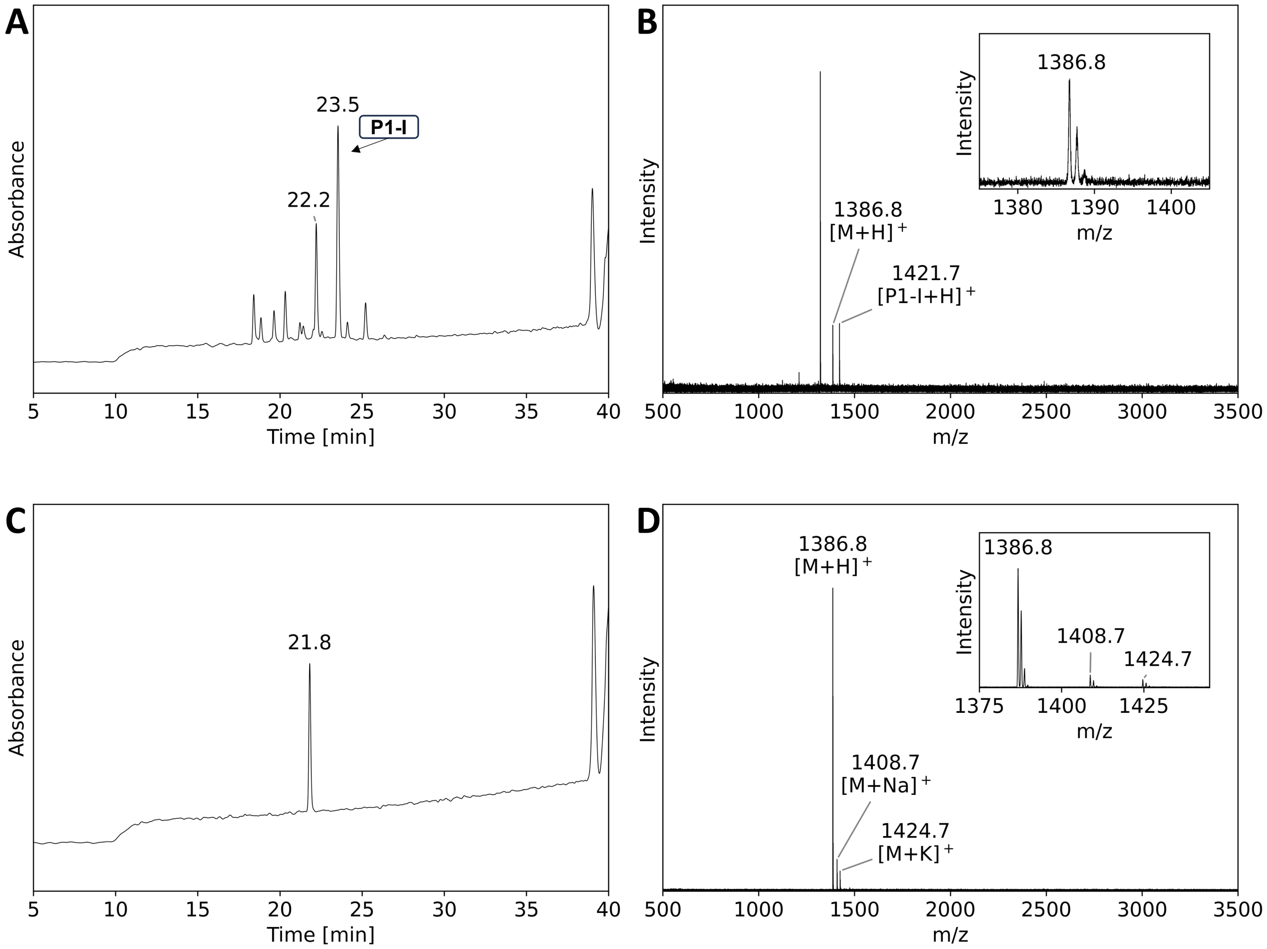

Figure S25: **P1-N21** A) Analytical HPLC (220 nm) of crude peptide (from **P1-OH** Batch 2). B) MALDI-TOF MS of crude peptide (from **P1-OH** Batch 2). C) Analytical HPLC (220 nm) of purified peptide. D) MALDI-TOF MS of purified peptide. Calculated mass: $[M+H]^+$ 1386.8, $[M+Na]^+$ 1408.8, $[M+K]^+$ 1424.7, $[P1\text{-}I+H]^+$ 1421.6.

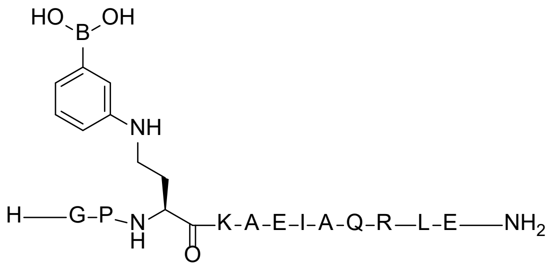

P1-N22

Chemical Formula: $C_{62}H_{104}BN_{19}O_{19}$
Exact Mass: 1429.78
Molecular Weight: 1430.44

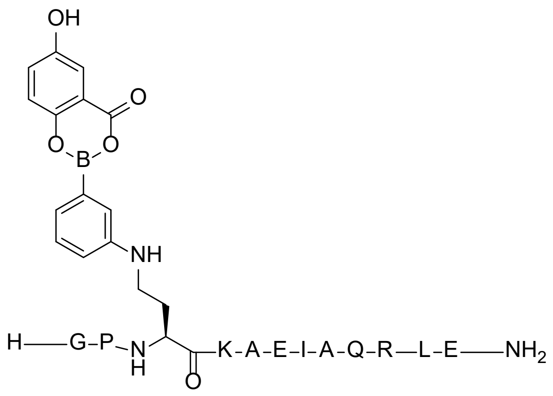

P1-N22 x DHB

Chemical Formula: $C_{69}H_{106}BN_{19}O_{21}$
Exact Mass: 1547.79
Molecular Weight: 1548.53

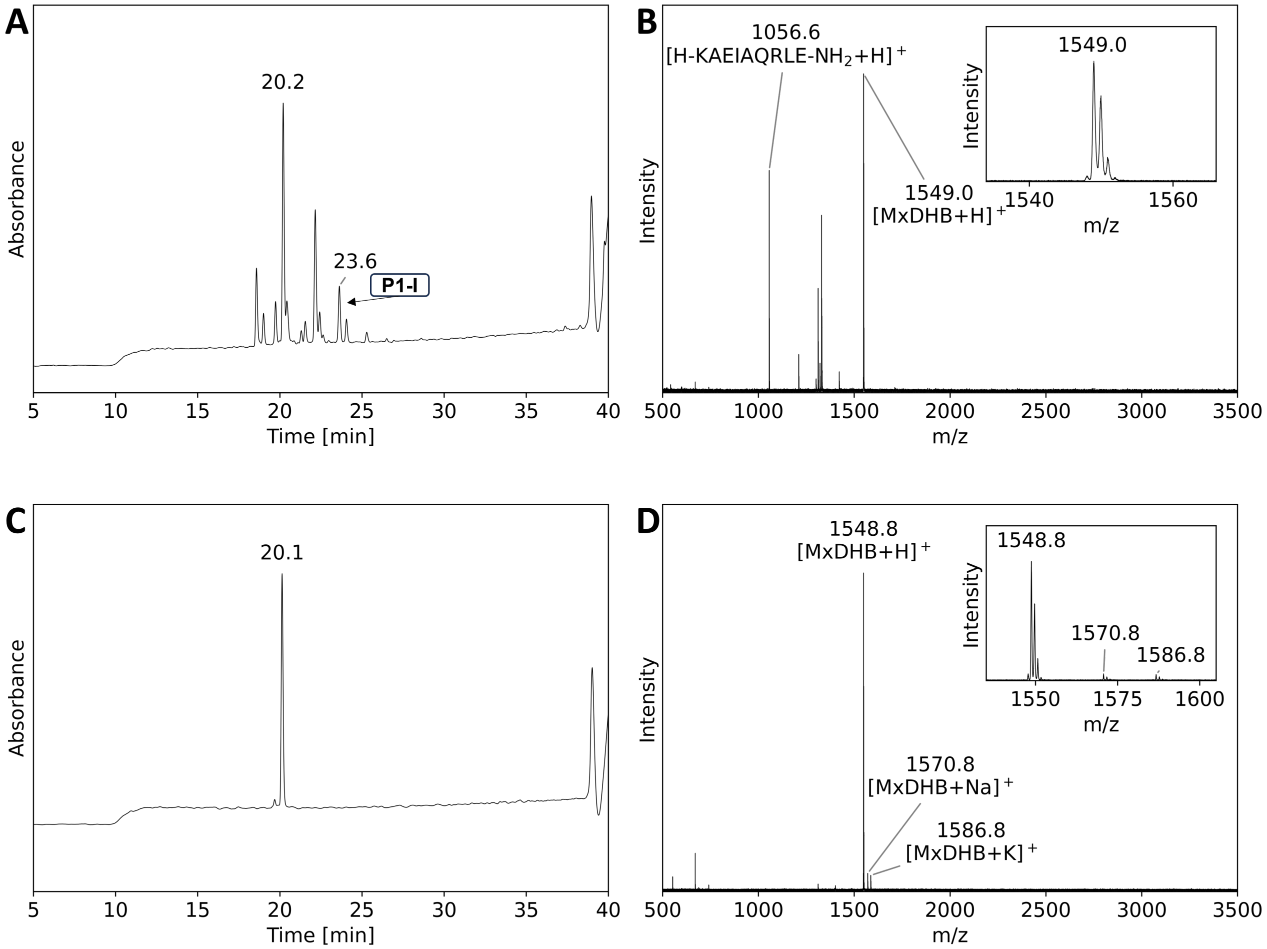

Figure S26: **P1-N22** A) Analytical HPLC (220 nm) of crude peptide (from **P1-OH** Batch 1 with **N22** in DMF). B) MALDI-TOF MS of crude peptide (from **P1-OH** Batch 1 with **N22** in DMF). C) Analytical HPLC (220 nm) of purified peptide. D) MALDI-TOF MS of purified peptide. It is known from previous studies that the iodinated peptide can react in a self-cleavage reaction forming the *N*-terminal peptide fragment.[1] The formation of boronate esters with 2,5-dihydroxybenzoic acid (DHB) was observed, which is described in the literature.[2] Calculated mass: $[H\text{-}KAEIAQRLE\text{-}NH_2+H]^+$ 1056.6, $[MxDHB+H]^+$ 1548.8, $[MxDHB+Na]^+$ 1570.8, $[MxDHB+K]^+$ 1586.8.

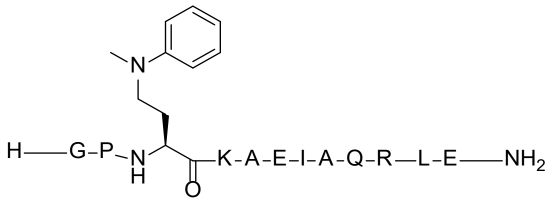

P1-N23

Chemical Formula: $C_{63}H_{105}N_{19}O_{17}$
Exact Mass: 1399.79
Molecular Weight: 1400.65

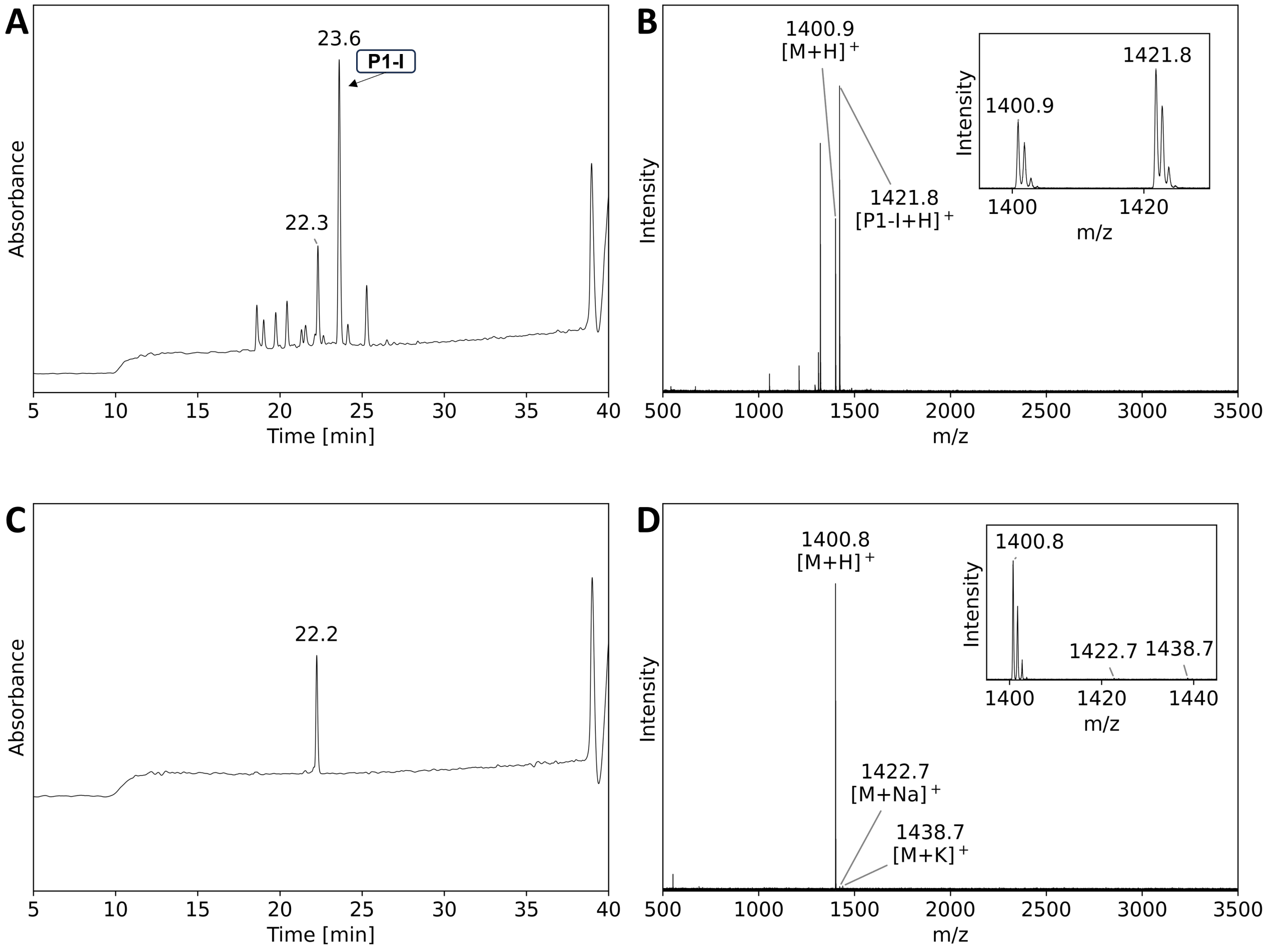

Figure S27: **P1-N23** A) Analytical HPLC (220 nm) of crude peptide (from **P1-OH** Batch 1). B) MALDI-TOF MS of crude peptide (from **P1-OH** Batch 1). C) Analytical HPLC (220 nm) of purified peptide. D) MALDI-TOF MS of purified peptide. Calculated mass: $[M+H]^+$ 1400.8, $[M+Na]^+$ 1422.8, $[M+K]^+$ 1438.8, $[P1\text{-}I+H]^+$ 1421.6.

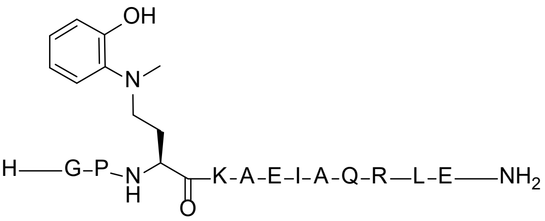

P1-N24
Chemical Formula: $C_{63}H_{105}N_{19}O_{18}$
Exact Mass: 1415.79
Molecular Weight: 1416.65

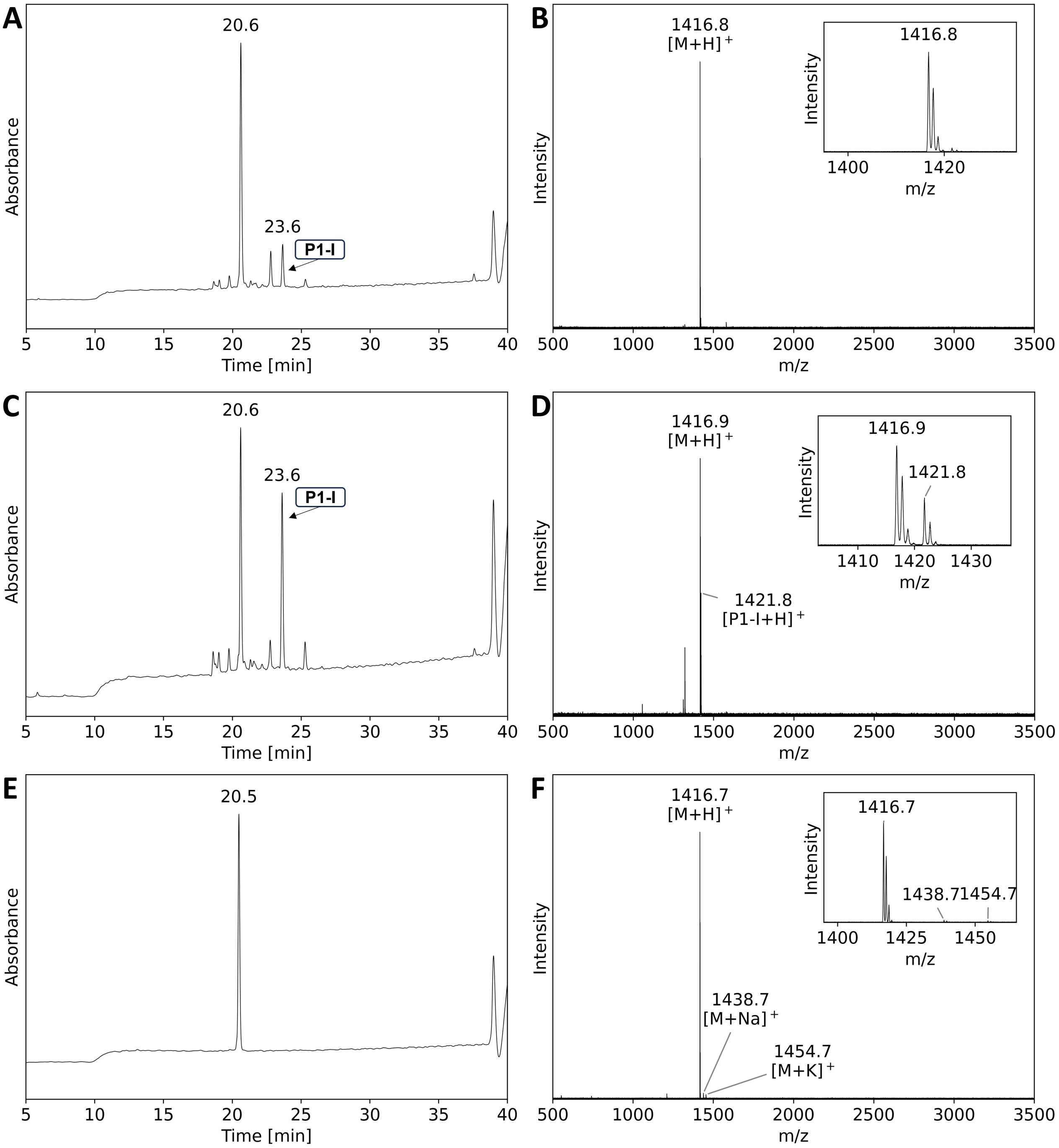

Figure S28: **P1-N24** A) Analytical HPLC (220 nm) of crude peptide (from **P1-OH** Batch 1; 2.5 M solution of **N24**). B) MALDI-TOF MS of crude peptide (from **P1-OH** Batch 1; 2.5 M solution of **N24**). C) Analytical HPLC (220 nm) of crude peptide (from **P1-OH** Batch 1). D) MALDI-TOF MS of crude peptide (from **P1-OH** Batch 1). E) Analytical HPLC (220 nm) of purified peptide. F) MALDI-TOF MS of purified peptide. Calculated mass: $[M+H]^+$ 1416.8, $[M+Na]^+$ 1438.8, $[M+K]^+$ 1454.8, $[P1\text{-}I+H]^+$ 1421.6.

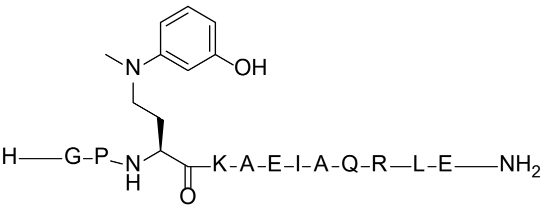

P1-N25

Chemical Formula: $C_{63}H_{105}N_{19}O_{18}$

Exact Mass: 1415.79

Molecular Weight: 1416.65

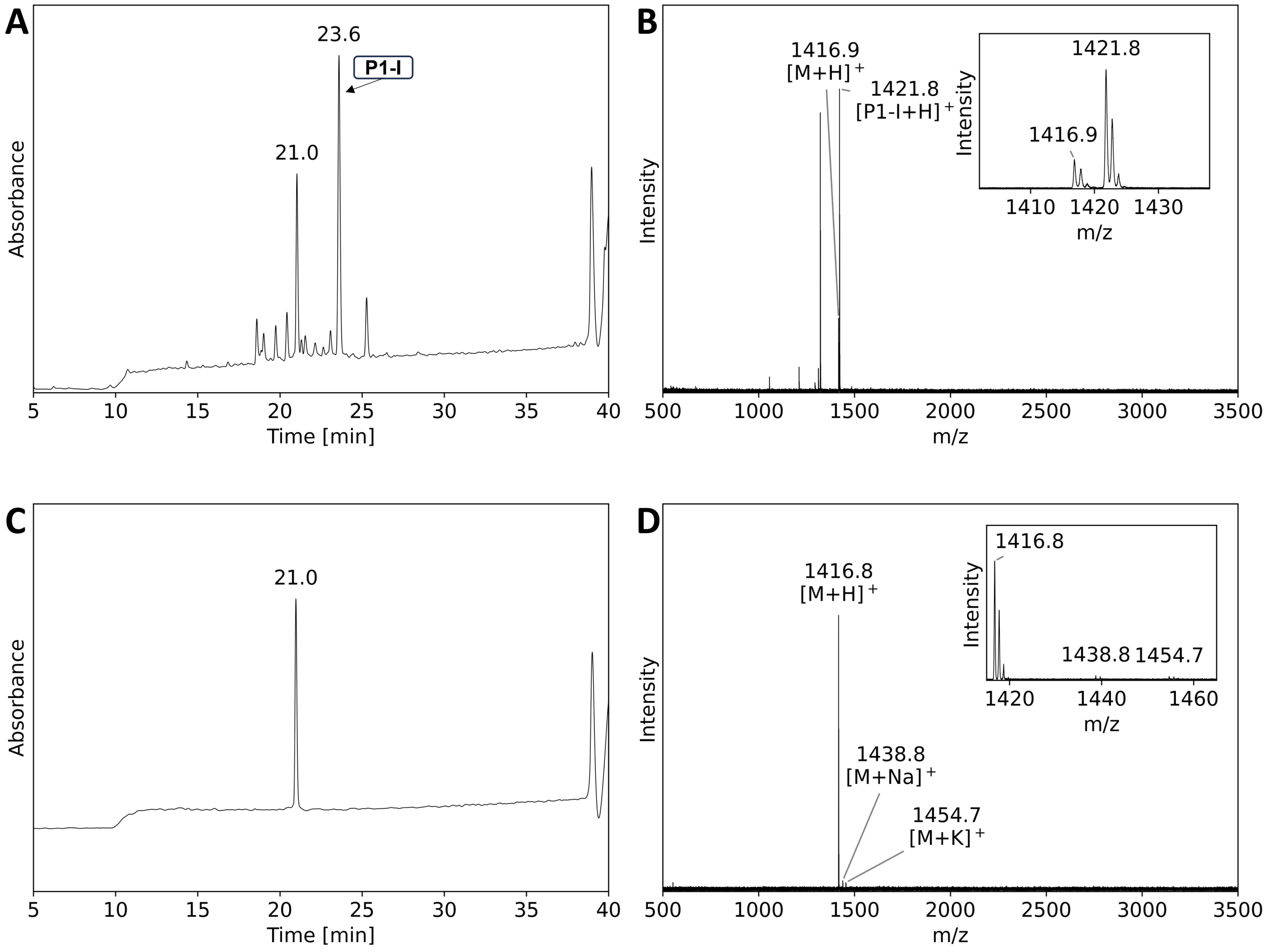

Figure S29: **P1-N25** A) Analytical HPLC (220 nm) of crude peptide (from **P1-OH** Batch 1). B) MALDI-TOF MS of crude peptide (from **P1-OH** Batch 1). C) Analytical HPLC (220 nm) of purified peptide. D) MALDI-TOF MS of purified peptide. Calculated mass: $[M+H]^+$ 1416.8, $[M+Na]^+$ 1438.8, $[M+K]^+$ 1454.8, $[P1\text{-}I+H]^+$ 1421.6.

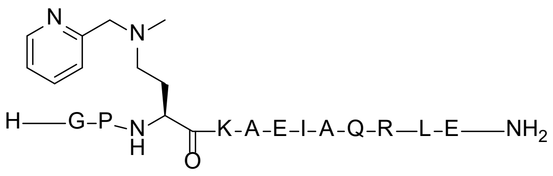

P1-N26

Chemical Formula: $C_{63}H_{106}N_{20}O_{17}$
Exact Mass: 1414.80
Molecular Weight: 1415.66

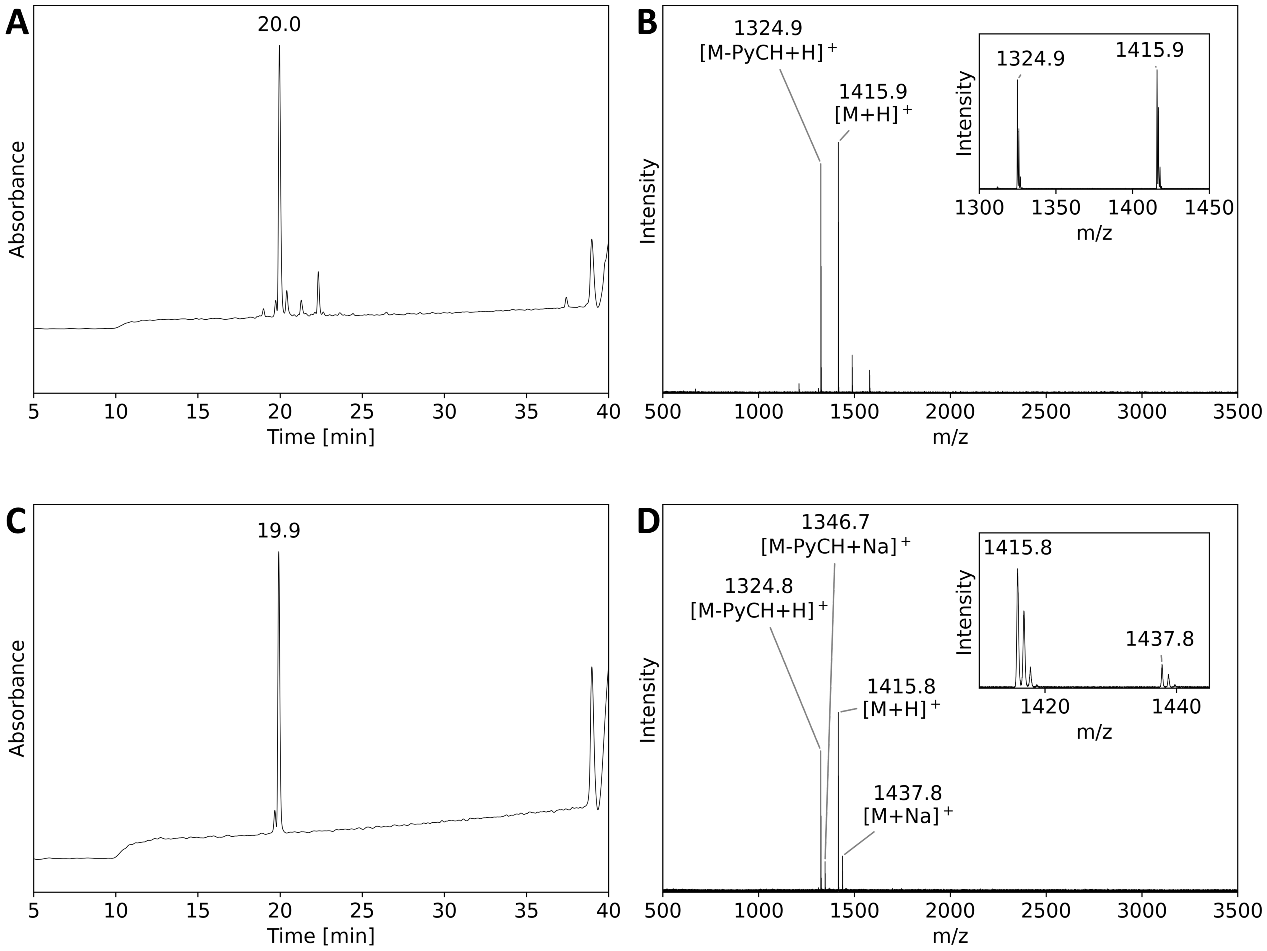

Figure S30: **P1-N26** A) Analytical HPLC (220 nm) of crude peptide (from **P1-OH** Batch 1). B) MALDI-TOF MS of crude peptide (from **P1-OH** Batch 1). C) Analytical HPLC (220 nm) of purified peptide. D) MALDI-TOF MS of purified peptide. Fragmentation of the picolyl substituent was observed in MALDI-TOF MS. Calculated mass: $[M\text{-}PyCH+H]^+$ 1324.8, $[M\text{-}PyCH+Na]^+$ 1346.8, $[M+H]^+$ 1415.8, $[M+Na]^+$ 1437.8.

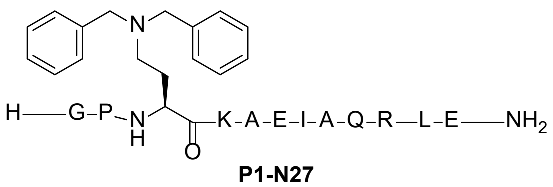

P1-N27
Chemical Formula: $C_{70}H_{111}N_{19}O_{17}$
Exact Mass: 1489.84
Molecular Weight: 1490.77

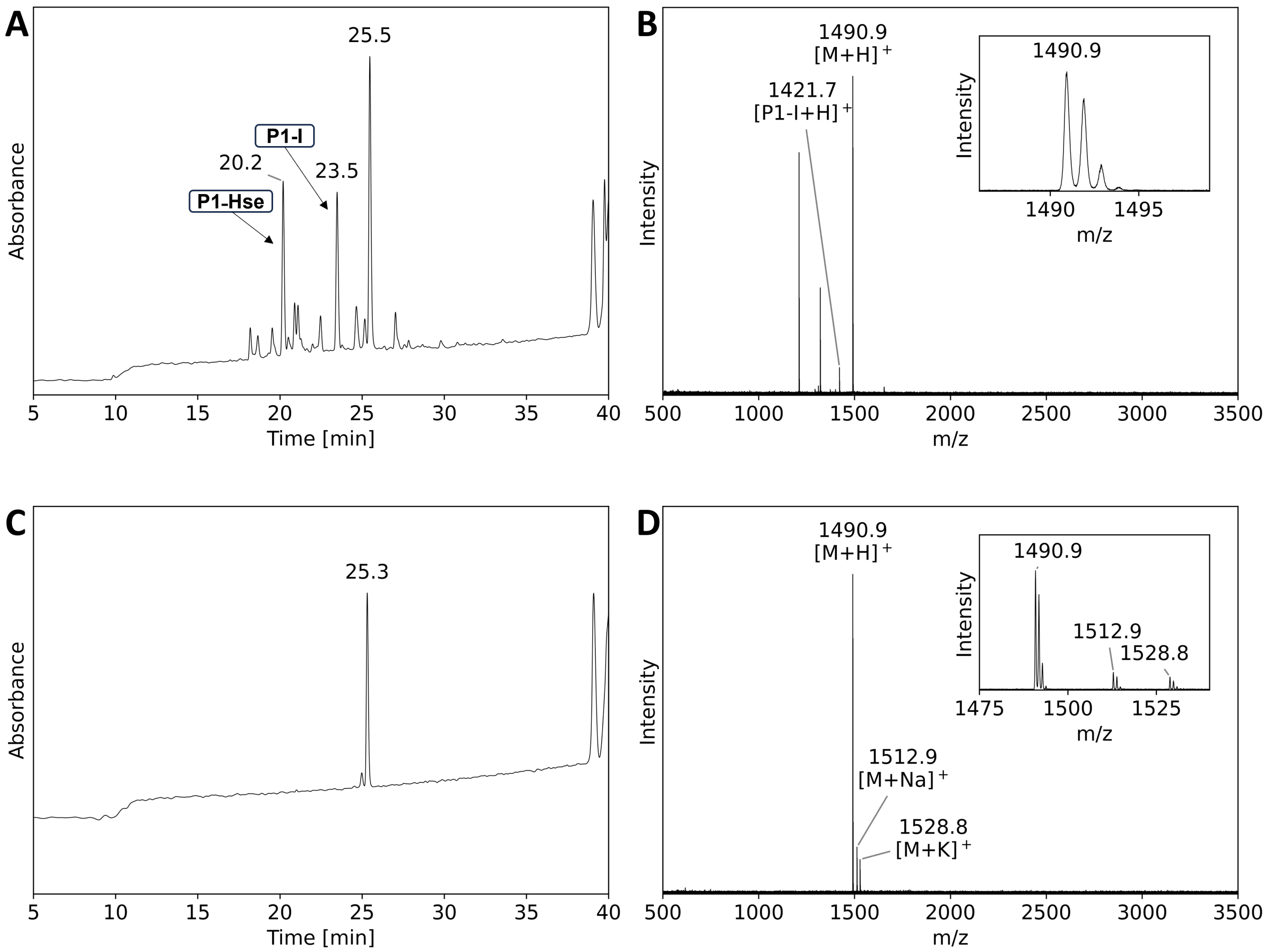

Figure S31: **P1-N27** A) Analytical HPLC (220 nm) of crude peptide (from **P1-OH** Batch 2). B) MALDI-TOF MS of crude peptide (from **P1-OH** Batch 2). C) Analytical HPLC (220 nm) of purified peptide. D) MALDI-TOF MS of purified peptide. Calculated mass: $[M+H]^+$ 1490.8, $[M+Na]^+$ 1512.8, $[M+K]^+$ 1528.8, $[P1\text{-}I+H]^+$ 1421.6.

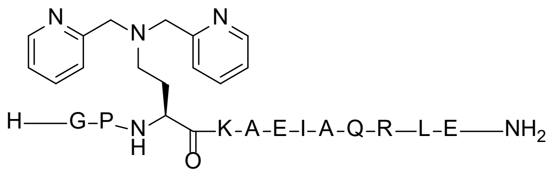

P1-N28
Chemical Formula: $C_{68}H_{109}N_{21}O_{17}$
Exact Mass: 1491.83
Molecular Weight: 1492.75

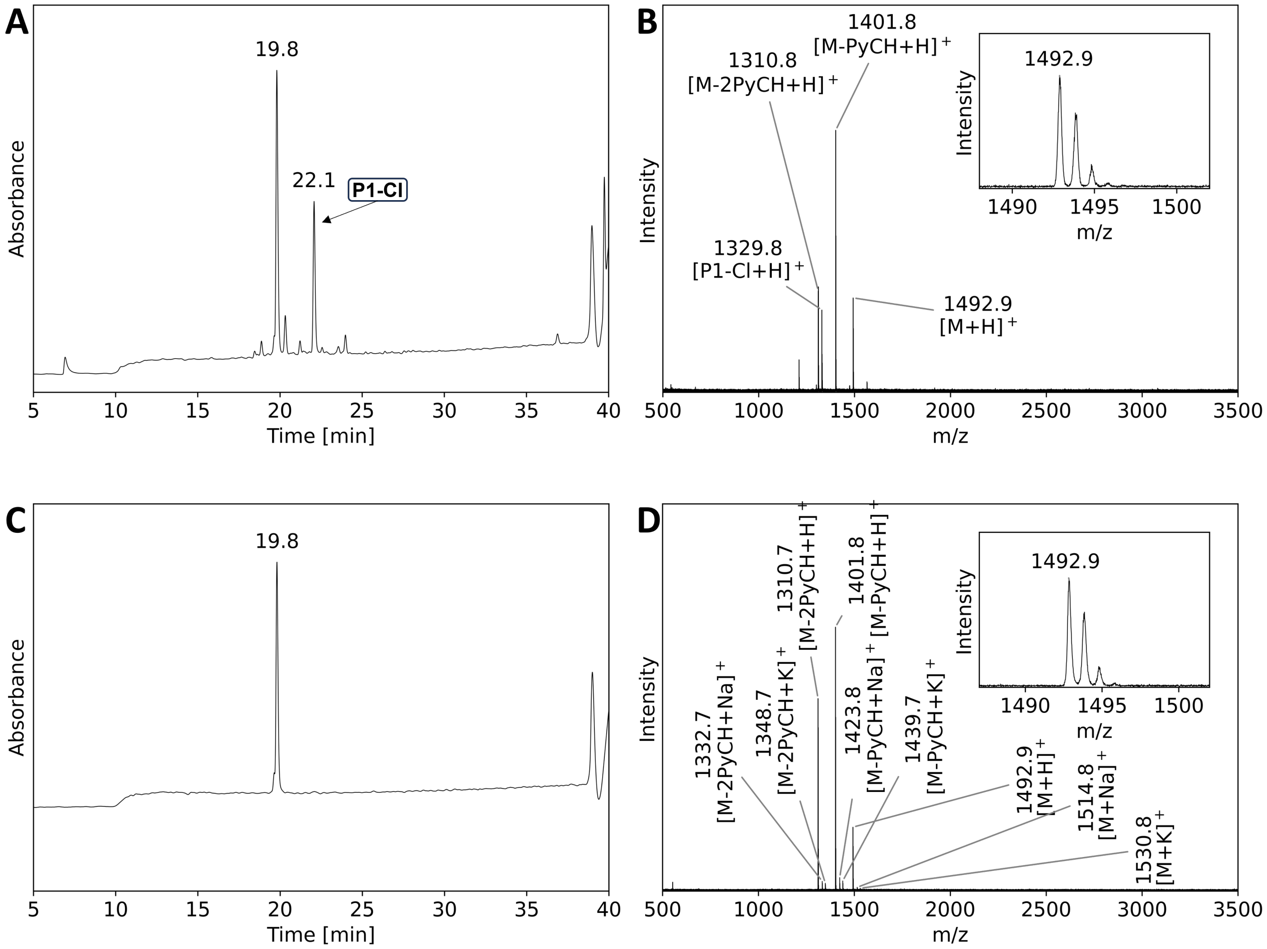

Figure S32: **P1-N28** A) Analytical HPLC (220 nm) of crude peptide (from **P1-OH** Batch 1). B) MALDI-TOF MS of crude peptide (from **P1-OH** Batch 1). C) Analytical HPLC (220 nm) of purified peptide. D) MALDI-TOF MS of purified peptide. Fragmentation of both picolyl substituents was observed in MALDI-TOF MS. Calculated mass: $[M+H]^+$ 1492.8, $[M+Na]^+$ 1514.8, $[M+K]^+$ 1530.8, $[M\text{-}PyCH+H]^+$ 1401.8, $[M\text{-}PyCH+Na]^+$ 1423.8, $[M\text{-}PyCH+K]^+$ 1439.8, $[M\text{-}2PyCH+H]^+$ 1310.8, $[M\text{-}2PyCH+Na]^+$ 1332.7, $[M\text{-}2PyCH+K]^+$ 1348.7, [P1-Cl+H] 1329.7.

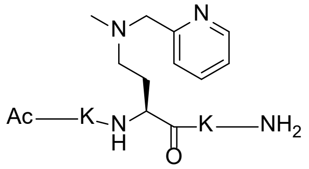

P2-N26

Chemical Formula: $C_{25}H_{44}N_8O_4$

Exact Mass: 520.35

Molecular Weight: 520.68

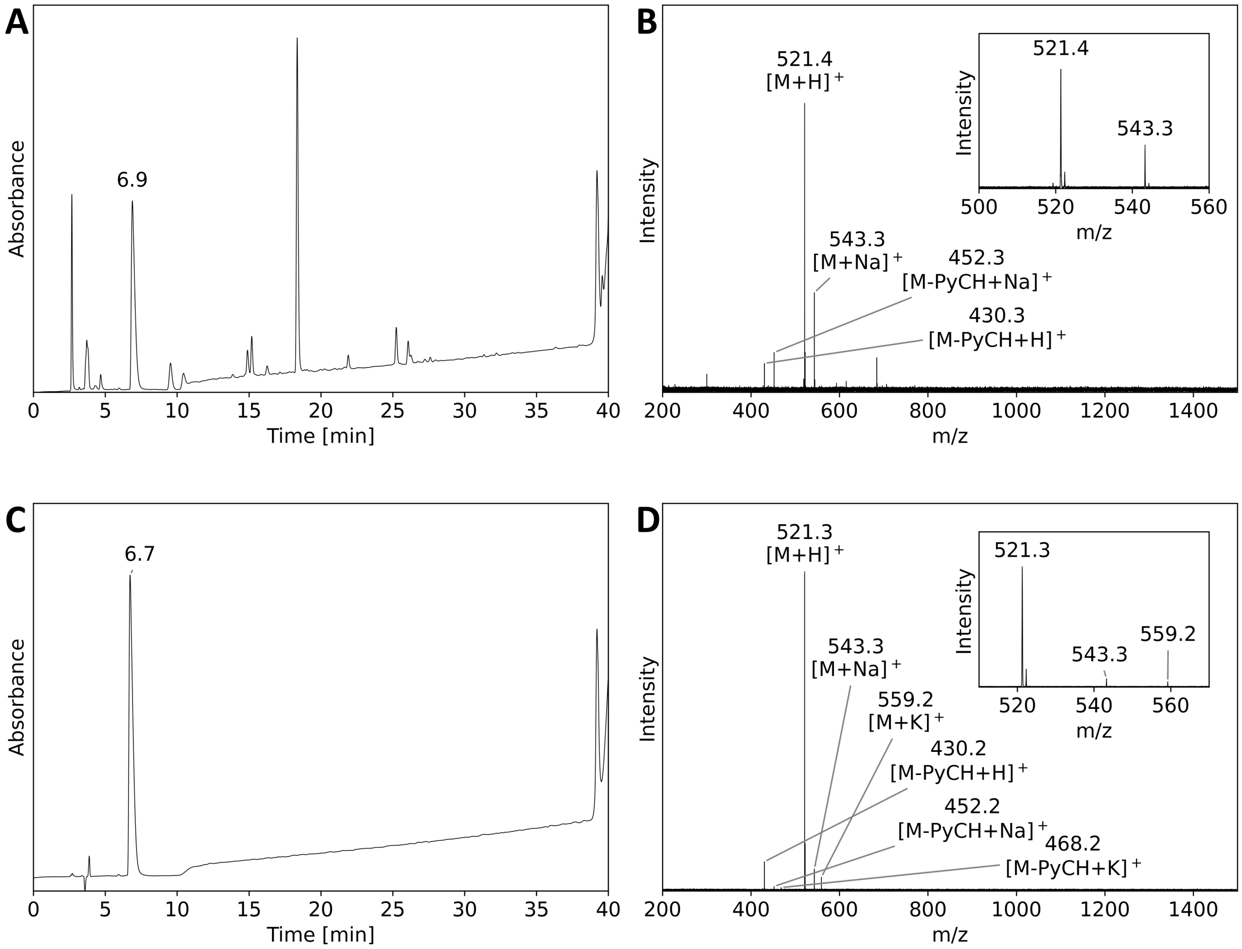

Figure S33: **P2-N26** A) Analytical HPLC (220 nm) of crude peptide. B) MALDI-TOF MS of crude peptide. C) Analytical HPLC (220 nm) of purified peptide. D) MALDI-TOF MS of purified peptide. Fragmentation of the picolyl substituent was observed in MALDI-TOF MS. Calculated mass: $[M\text{-}PyCH+H]^+$ 430.3, $[M\text{-}PyCH+Na]^+$ 452.3, $[M\text{-}PyCH+K]^+$ 468.3, $[M+H]^+$ 521.4, $[M+Na]^+$ 543.3, $[M+K]^+$ 559.3.

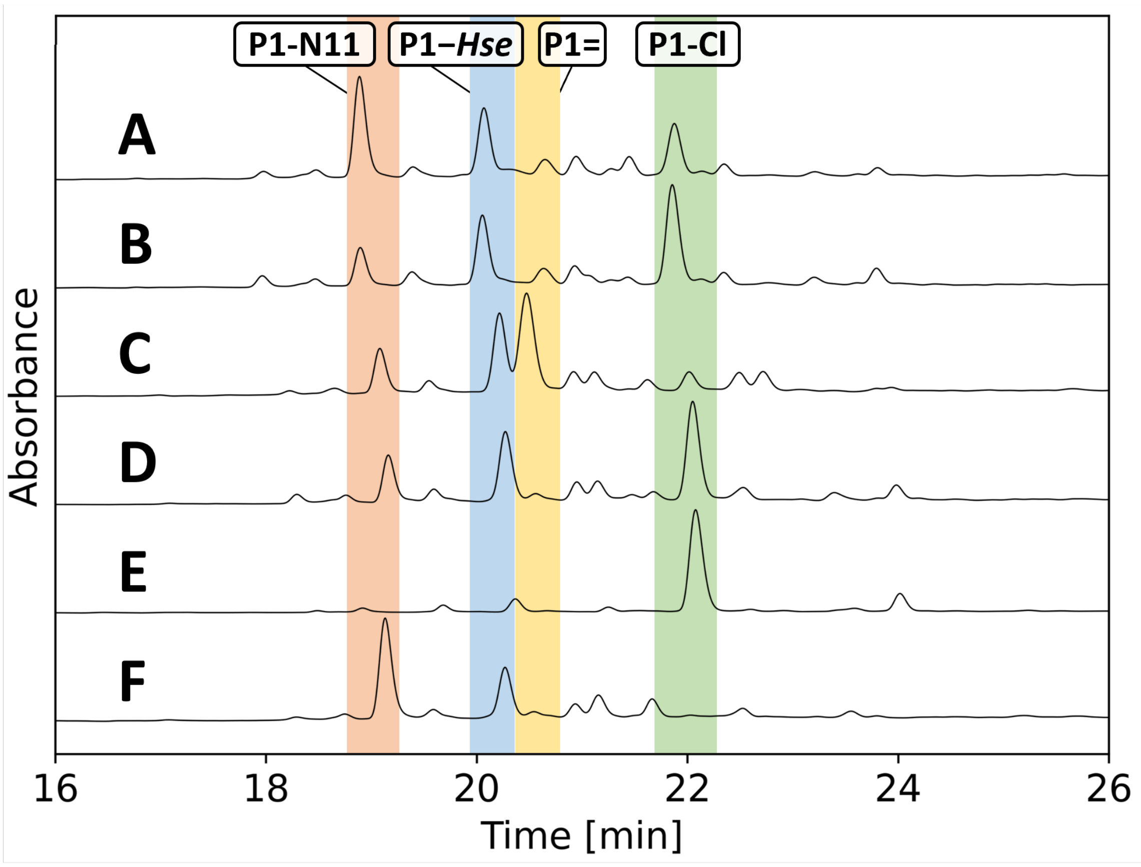

Figure S34: HPLC traces at 220 nm of the optimization of nucleophilic substitution with diethylammonium chloride **N11-HCl** compared to diethylamine **N11**. For all reactions with **N11-HCl** (A-E), 5 µmol of resin-bound peptide were reacted with 0.25 mL of a 1 M solution of the amine in DMF. In the case of **N11** (F), the reaction was carried out in MeCN. **P1−Hse** is the peptide without homoserine observed in Batch 2 of **P1-OH** due to incomplete coupling during SPPS. A) DBU (140 mM) was added. B) DIPEA (10 M) was added. C) DBU (1.0 M) was added. D) DIPEA (1.0 M) was added. E) No additive. F) **N11** was used as nucleophile.

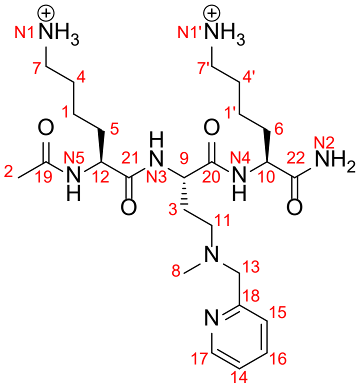

^{1}H NMR (400 MHz, DMSO-*d*6, 295 K):

δ [ppm] = 8.69-8.64 (m, 1H, H-17), 8.20 (d, *J* = 7.9 Hz, 1H, H-N3), 8.10 (d, *J* = 7.2 Hz, 1H, H-N5), 7.96-7.89 (m, 2H, H-16/N4), 7.80 (br, 6H, H-N1), 7.53 (d, *J* = 7.7 Hz, 1H, H-15), 7.49 (ddd, *J* = 7.6, 4.8, 1.0 Hz, 1H, H-14), 7.40 (s, 1H, H-N2), 7.07 (s, 1H, H-N2), 4.47 (d, *J* = 4.0 Hz, 2H, H-13), 4.34 (td, *J* = 12.1, 5.3 Hz, 1H, H-9), 4.13 (td, *J* = 12.1, 5.1 Hz, 2H, H-12/10), 3.28-3.06 (m, 2H, H-11), 2.81-2.71 (m, 7H, H-8/7), 2.24-2.13 (m, 1H, H-3), 2.06-1.95 (m, 1H, H-3), 1.85 (s, 3H, H-2), 1.73-1.42 (m, 8H, H-6/5/4), 1.41-1.20 (m, 4H, H-1).

^{13}C NMR (101 MHz, DMSO-*d*6, 295 K):

δ [ppm] = 173.3 (C-22), 172.4 (C-21), 170.0 (C-20), 169.8 (C-19), 150.5 (C-18), 149.6 (C-17), 137.8 (C-16), 124.6 (C-15), 124.2 (C-14), 58.9 (C-13), 52.9 (C-12), 52.8 (C-11), 52.4 (C-10), 50.0 (C-9), 40.3 (C-8), 38.72 (C-7), 38.67 (C-7), 31.4 (C-6), 31.0 (C-5), 26.7 (C-4), 26.3 (C-3), 22.5 (C-2), 22.3 (C-1).

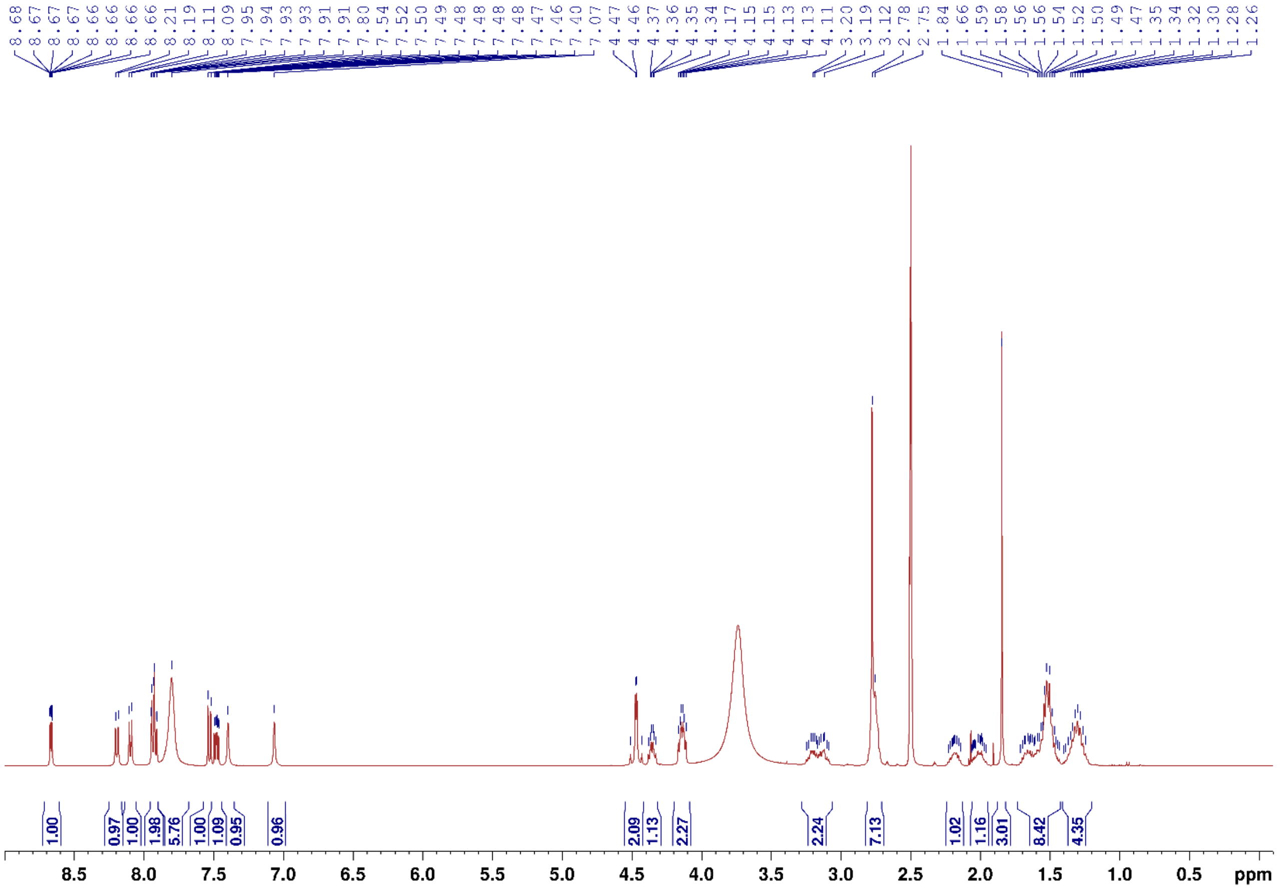

Figure S35: ^{1}H NMR spectrum (400 MHz, DMSO-*d*6, 295 K) of **P2-N26**.

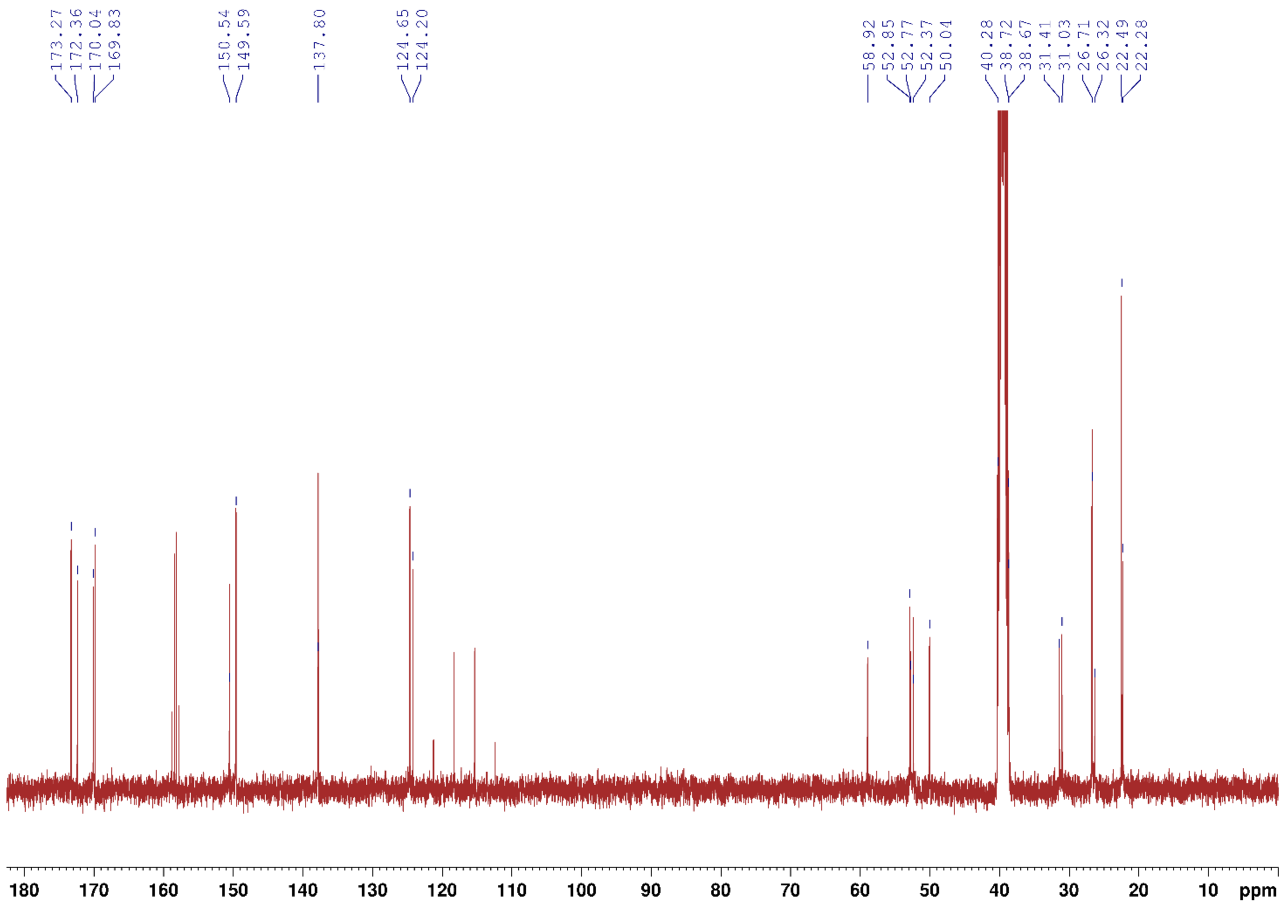

Figure S36: ^{13}C NMR spectrum (101 MHz, DMSO-d_6, 295 K) of **P2-N26**.

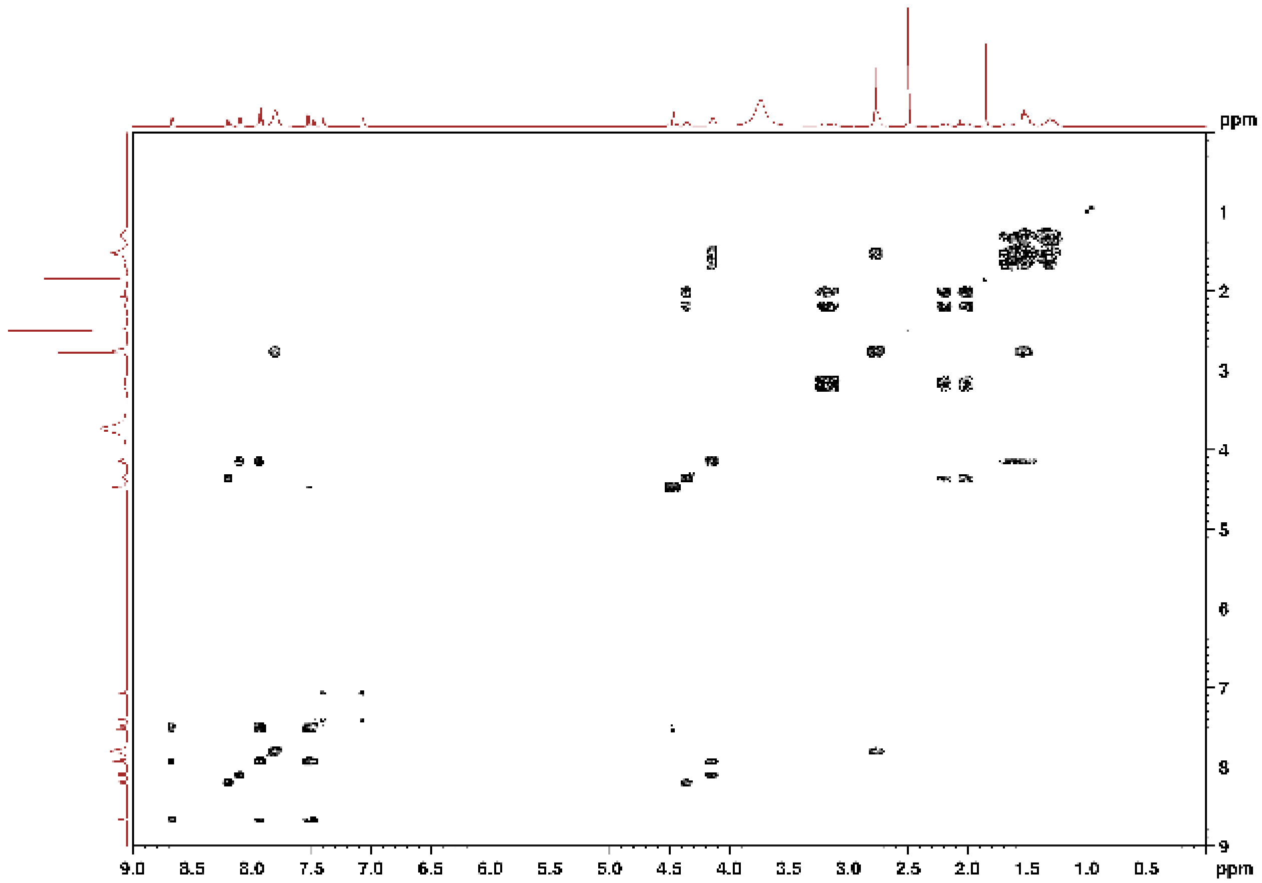

Figure S37: ^1H-^1H COSY spectrum (400 MHz, 400 MHz, DMSO-d_6, 295 K) of **P2-N26.**

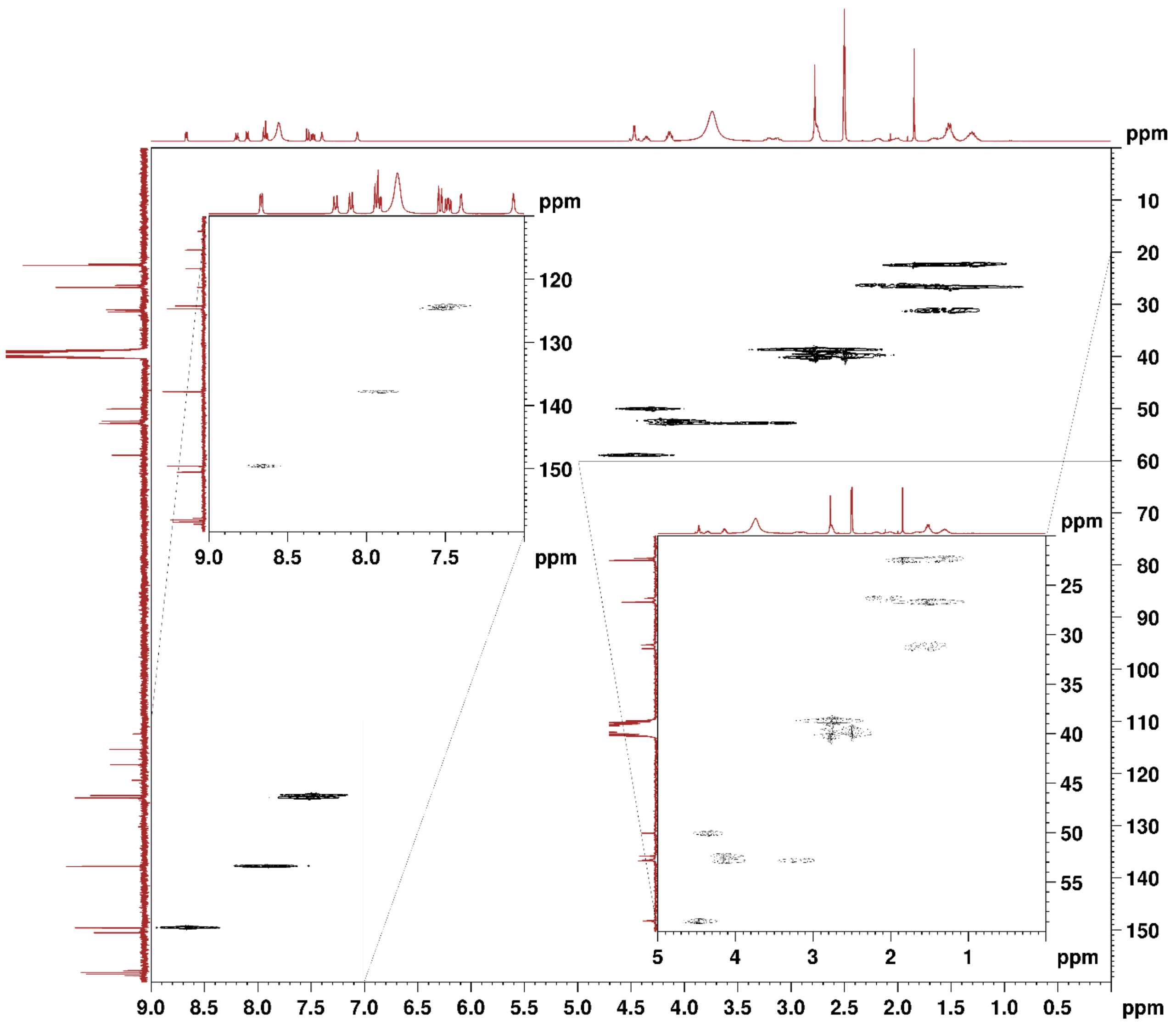

Figure S38: ^{1}H-^{13}C HSQC spectrum (400 MHz, 101 MHz, DMSO-d_6, 295 K) of **P2-N26**.

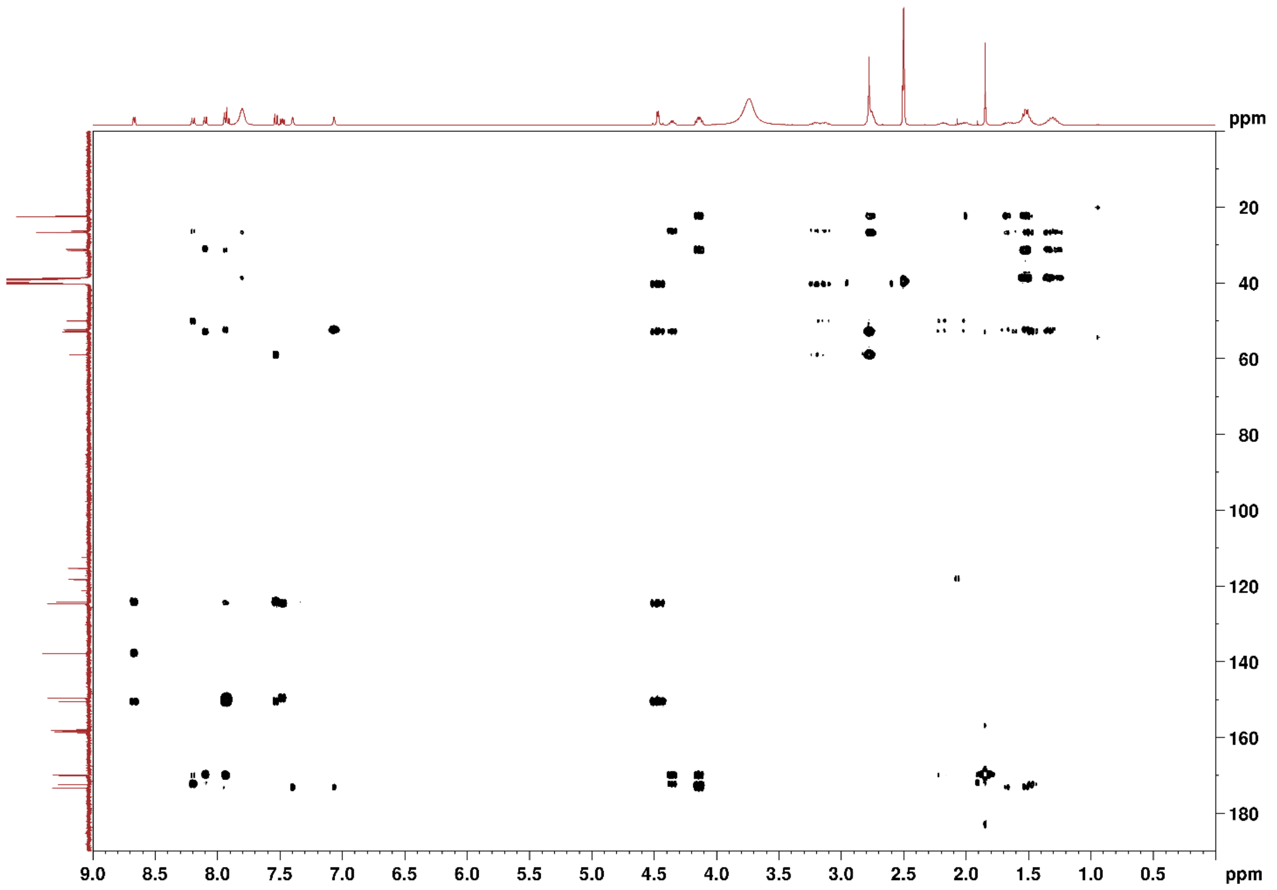

Figure S39: ^{1}H-^{13}C HMBC spectrum (400 MHz, 101 MHz, DMSO-d_6, 295 K) of **P2-N26**.

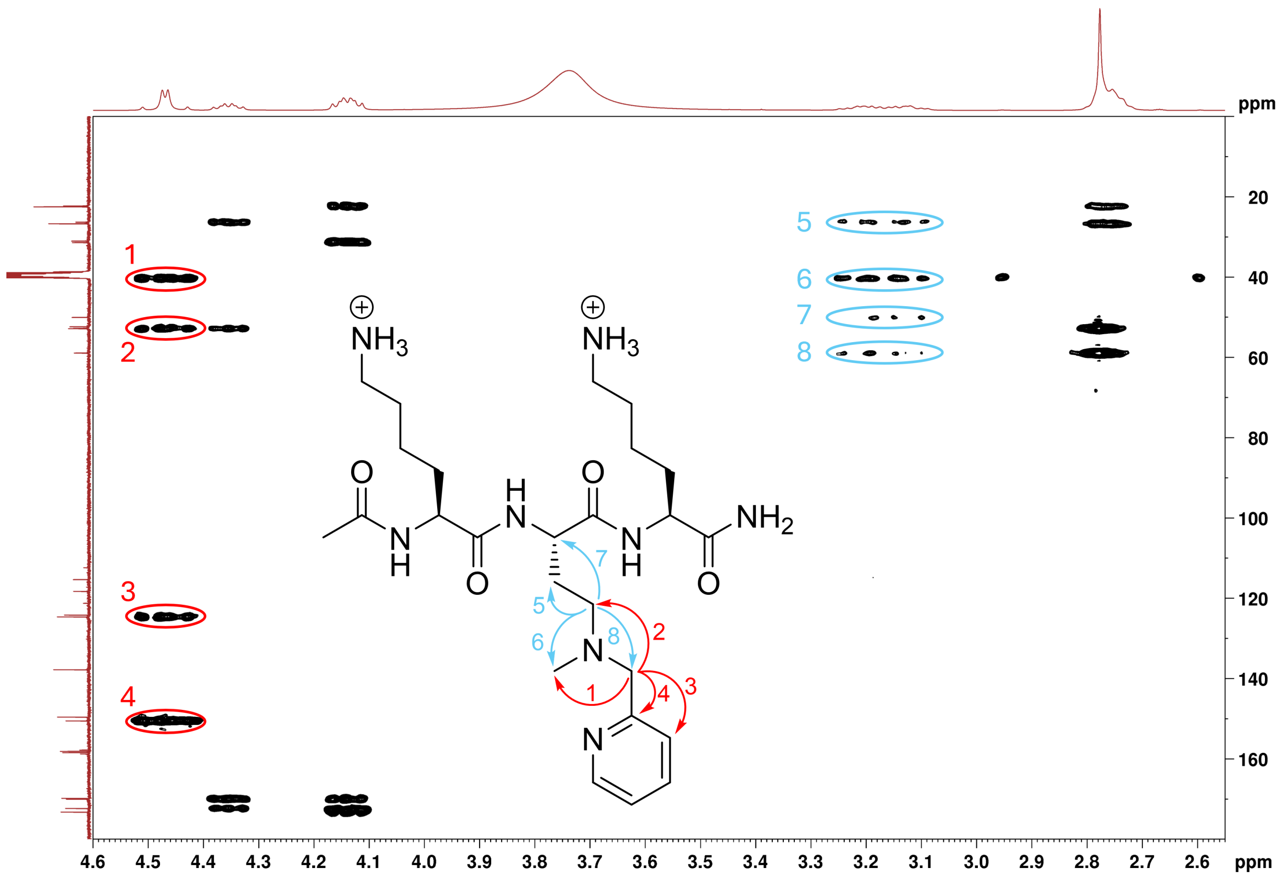

Figure S40: Extract from the ^{1}H-^{13}C HMBC spectrum (400 MHz, 101 MHz, DMSO-d_6, 295 K) of **P2-N26** showing the cross-peaks relevant for structure determination.

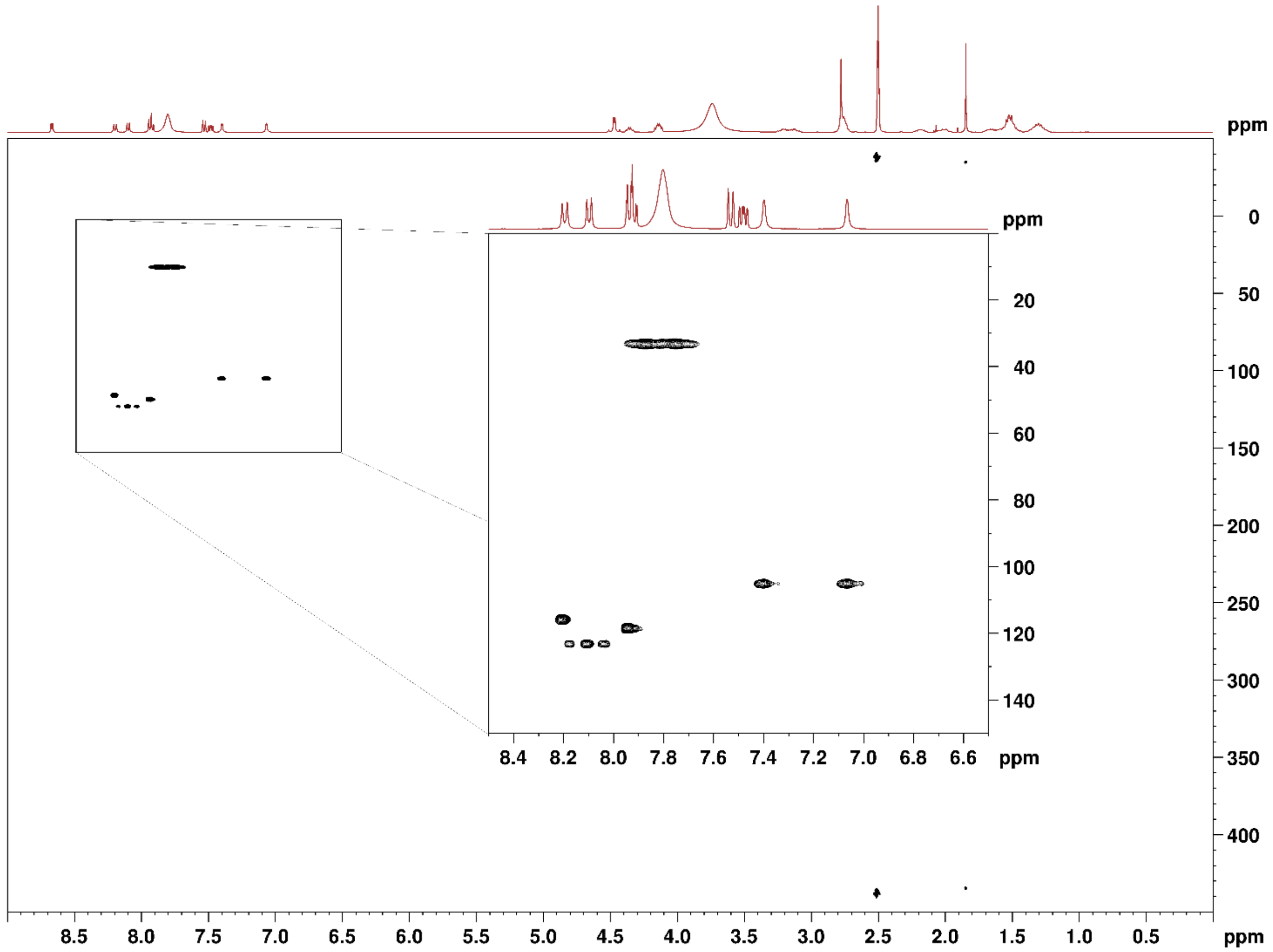

Figure S41: ^{1}H-^{15}N HSQC (400 MHz, 40.6 MHz, DMSO-d_6, 295 K) of **P2-N26**.

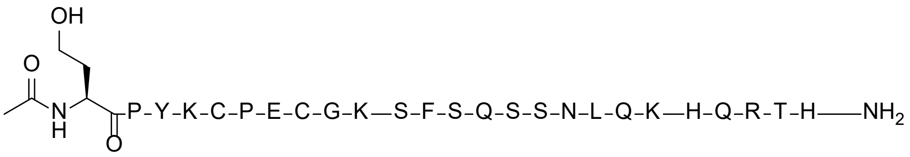

P3-OH

Chemical Formula: $C_{124}H_{194}N_{40}O_{39}S_2$
Exact Mass: 2931.39
Molecular Weight: 2933.28

Ac–P-Y-K-C-P-E-C-G-K—S-F-S-Q-S-S-N-L-Q-K—H-Q-R-T-H——NH_2

P3-Hse

Chemical Formula: $C_{120}H_{187}N_{39}O_{37}S_2$
Exact Mass: 2830.34
Molecular Weight: 2832.17

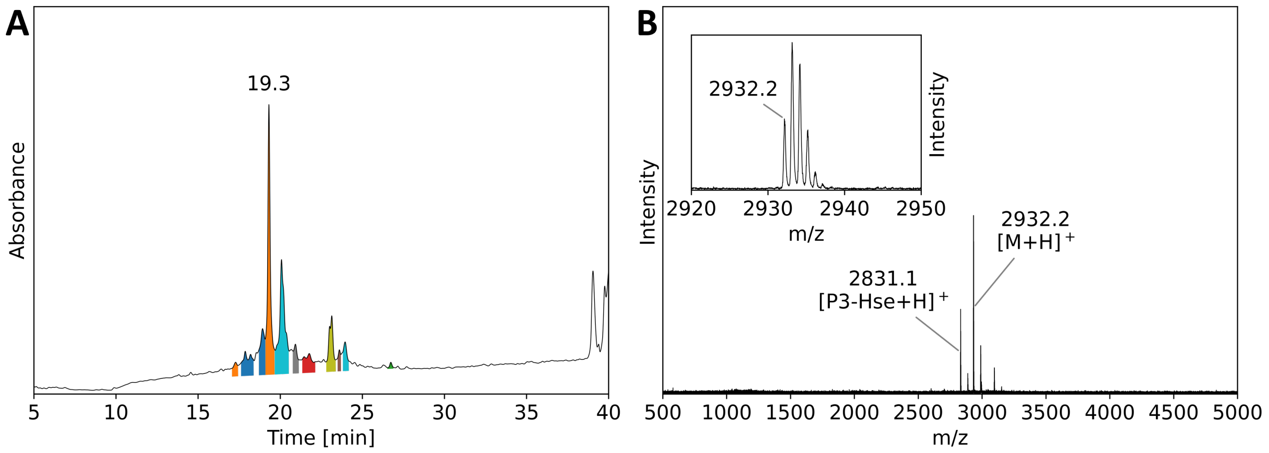

Figure S42: **P3-OH** A) Analytical HPLC (220 nm) of crude peptide. B) MALDI-TOF MS of crude peptide. Calculated mass: $[P3\text{-}Hse+H]^+$ 2831.3, $[M+H]^+$ 2932.4. Integrated areas are highlighted. Calculated Purity: 30 %.

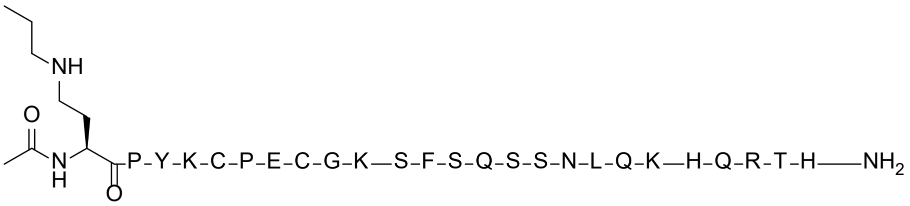

P3-N2

Chemical Formula: $C_{127}H_{201}N_{41}O_{38}S_2$

Exact Mass: 2972.45

Molecular Weight: 2974.37

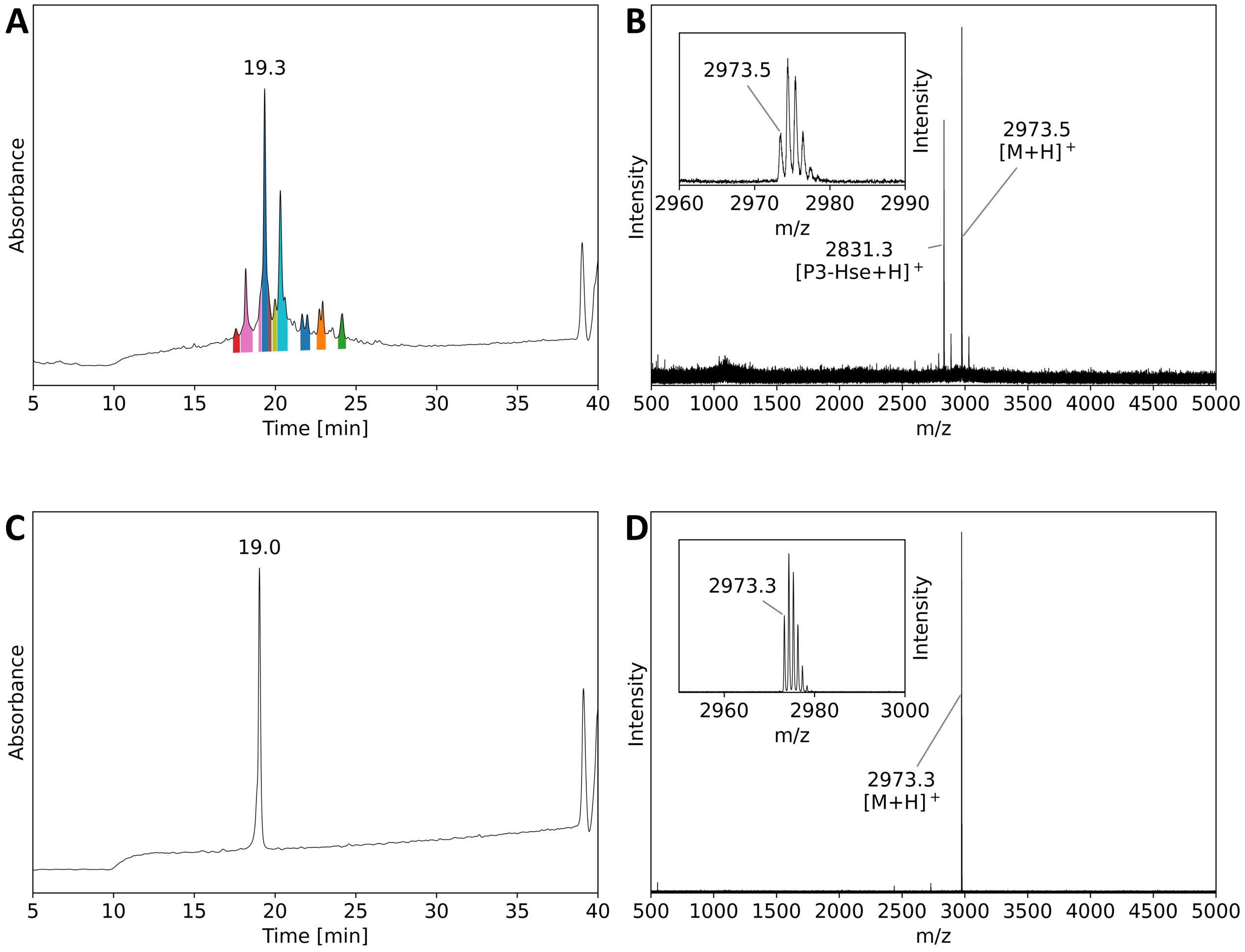

Figure S43: **P3-N2** A) Analytical HPLC (220 nm) of crude peptide. B) MALDI-TOF MS of crude peptide. C) Analytical HPLC (220 nm) of purified peptide. D) MALDI-TOF MS of purified peptide. Calculated mass: [P3-Hse+H]$^+$ 2831.3, [M+H]$^+$ 2973.5. Integrated areas are highlighted. Calculated purity: 23 %. Calculated yield: 75 %.

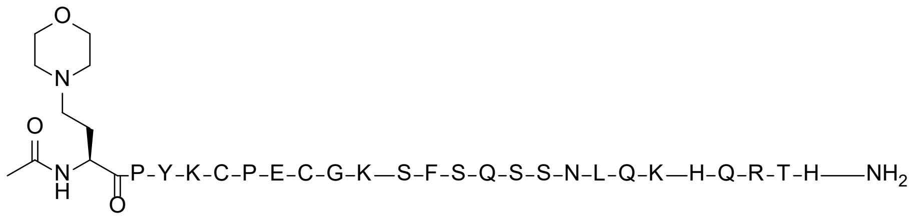

P3-N15

Chemical Formula: $C_{128}H_{201}N_{41}O_{39}S_2$

Exact Mass: 3000.44

Molecular Weight: 3002.38

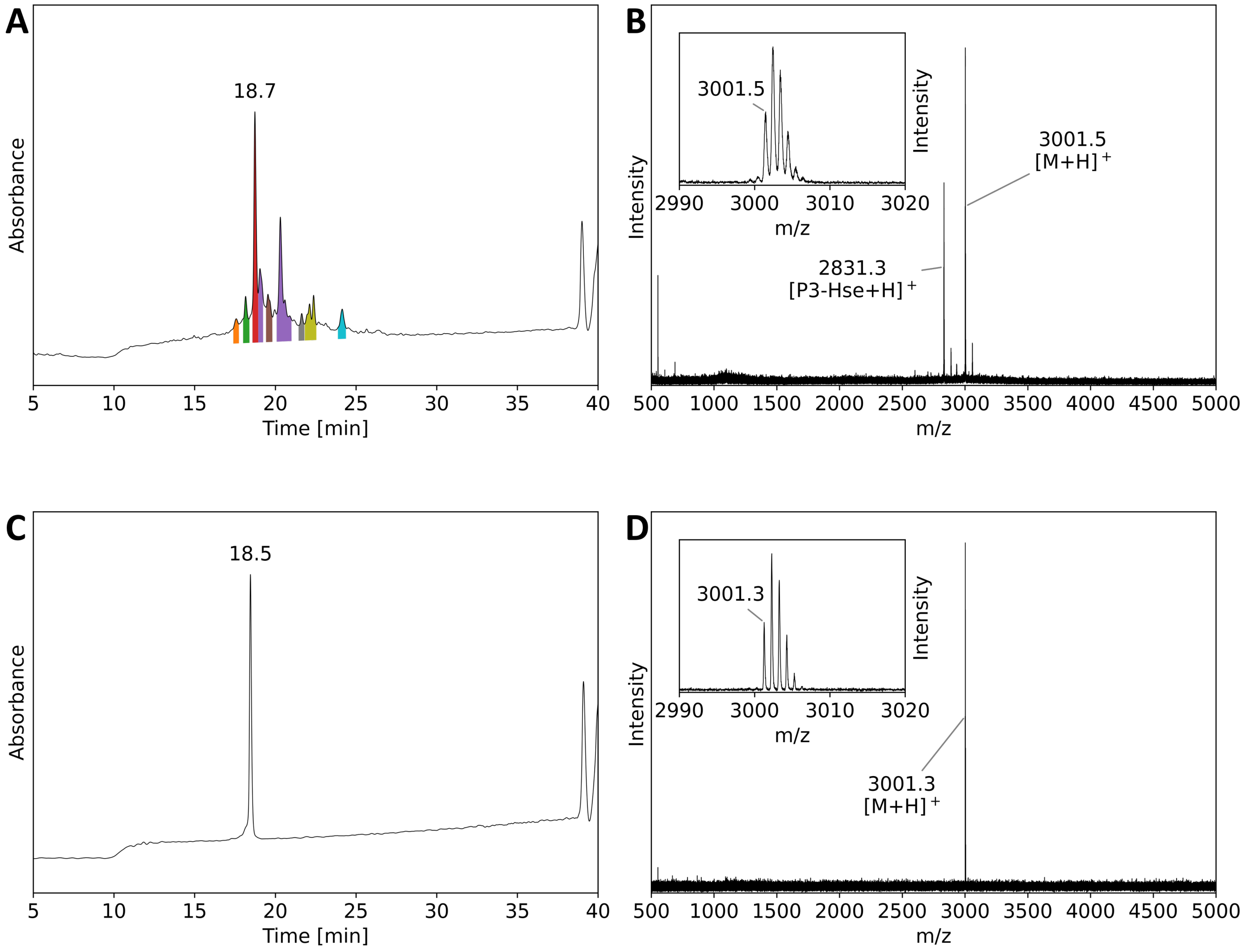

Figure S44: **P3-N15** A) Analytical HPLC (220 nm) of crude peptide. B) MALDI-TOF MS of crude peptide. C) Analytical HPLC (220 nm) of purified peptide. D) MALDI-TOF MS of purified peptide. Calculated mass: $[P3\text{-}Hse+H]^+$ 2831.3, $[M+H]^+$ 3001.5. Integrated areas are highlighted. Calculated purity: 24 %. Calculated yield: 80 %.

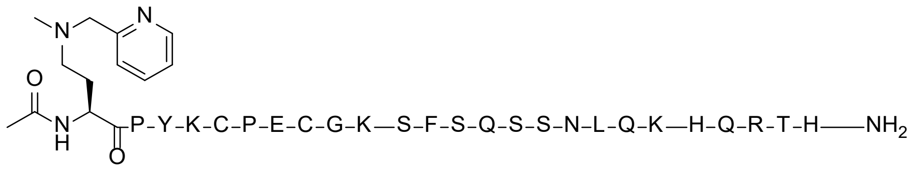

P3-N26

Chemical Formula: $C_{131}H_{202}N_{42}O_{38}S_2$

Exact Mass: 3035.46

Molecular Weight: 3037.43

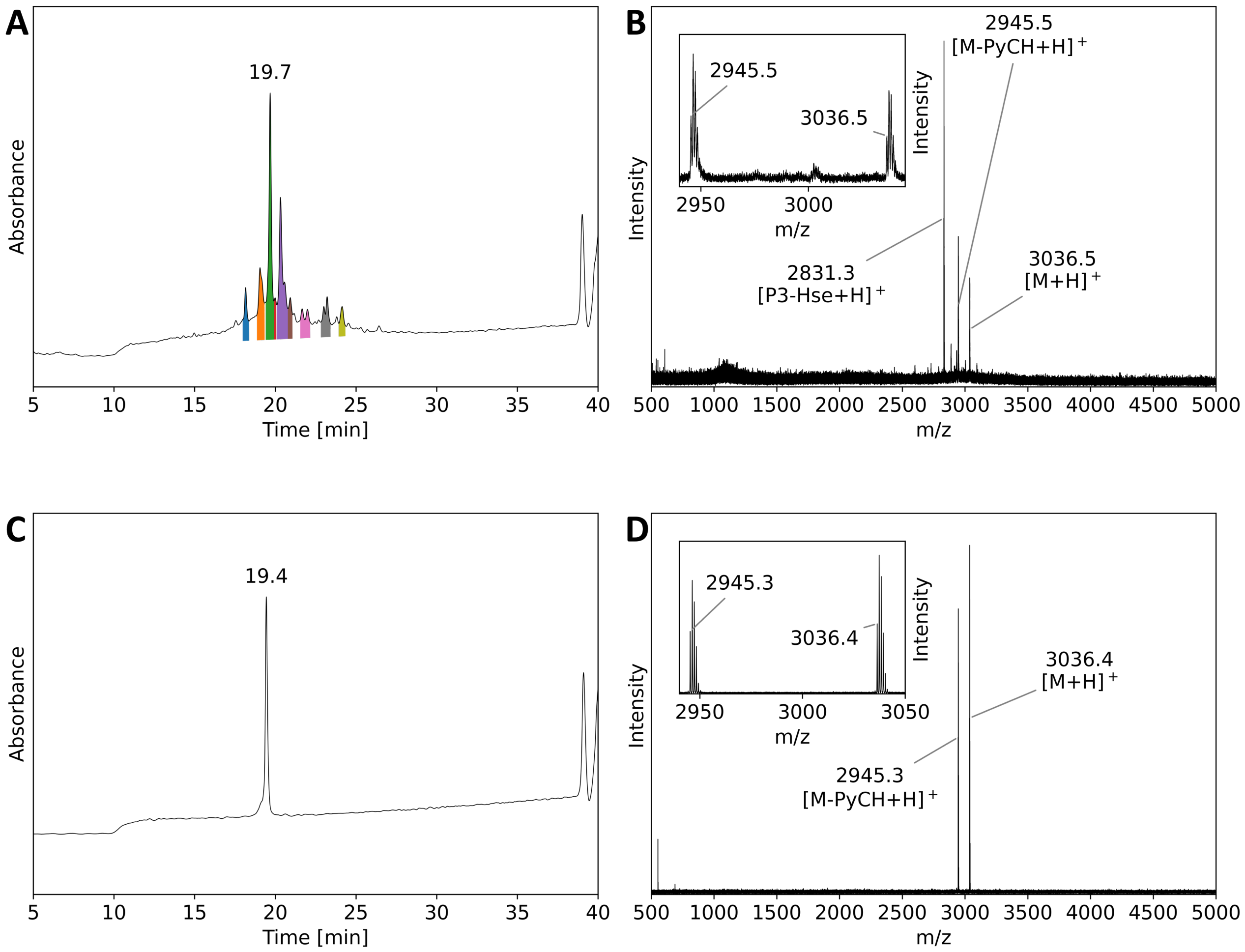

Figure S45: **P3-N26** A) Analytical HPLC (220 nm) of crude peptide. B) MALDI-TOF MS of crude peptide. C) Analytical HPLC (220 nm) of purified peptide. D) MALDI-TOF MS of purified peptide. Fragmentation of the picolyl substituent was observed in MALDI-TOF MS. Calculated mass: $[P3\text{-}Hse+H]^+$ 2831.3, $[M\text{-}PyCH+H]^+$ 2945.4, $[M+H]^+$ 3036.5. Integrated areas are highlighted. Calculated purity: 28 %. Calculated yield: 90 %.

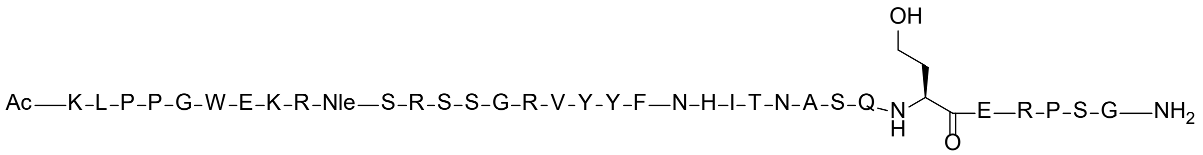

P4-OH

Chemical Formula: $C_{175}H_{271}N_{55}O_{51}$

Exact Mass: 3959.03

Molecular Weight: 3961.43

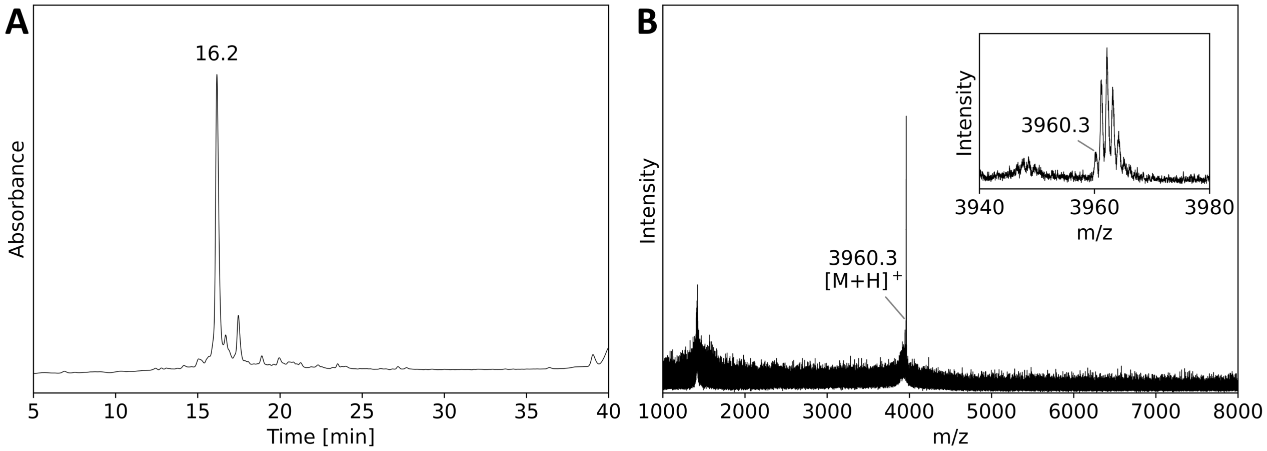

Figure S46: **P4-OH** A) Analytical HPLC (220 nm) of crude peptide. B) MALDI-TOF MS of crude peptide. Calculated mass: $[M+H]^+$ 3960.0. Calculated purity: 72 %.

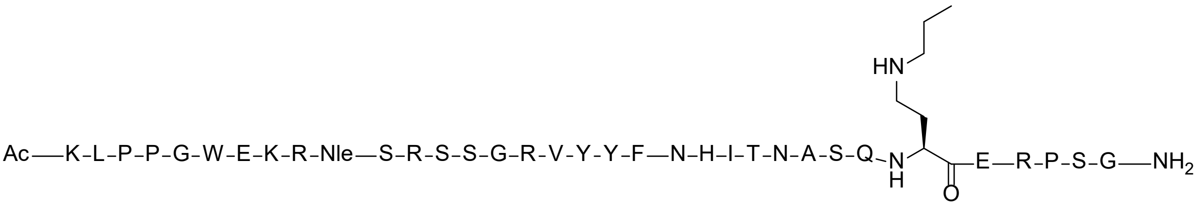

P4-N2
Chemical Formula: $C_{178}H_{278}N_{56}O_{50}$
Exact Mass: 4000.09
Molecular Weight: 4002.52

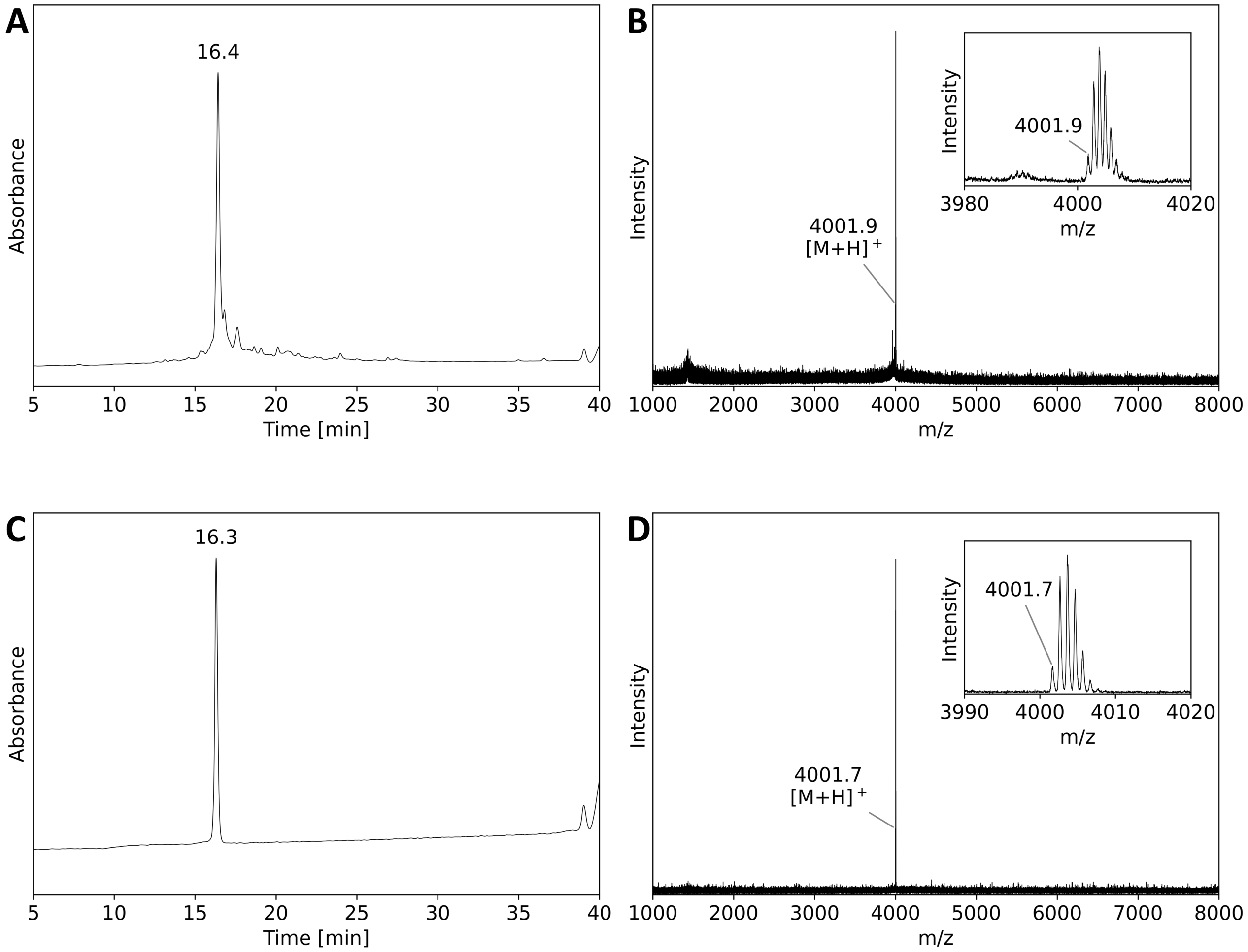

Figure S47: **P4-N2** A) Analytical HPLC (220 nm) of crude peptide. B) MALDI-TOF MS of crude peptide. C) Analytical HPLC (220 nm) of purified peptide. D) MALDI-TOF MS of purified peptide. Calculated mass: $[M+H]^+$ 4001.1. Calculated purity: 68 %. Calculated yield: 95 %.

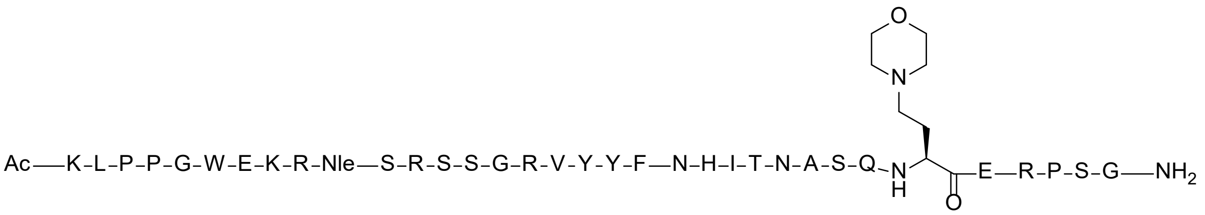

P4-N15
Chemical Formula: $C_{179}H_{278}N_{56}O_{51}$
Exact Mass: 4028.09
Molecular Weight: 4030.53

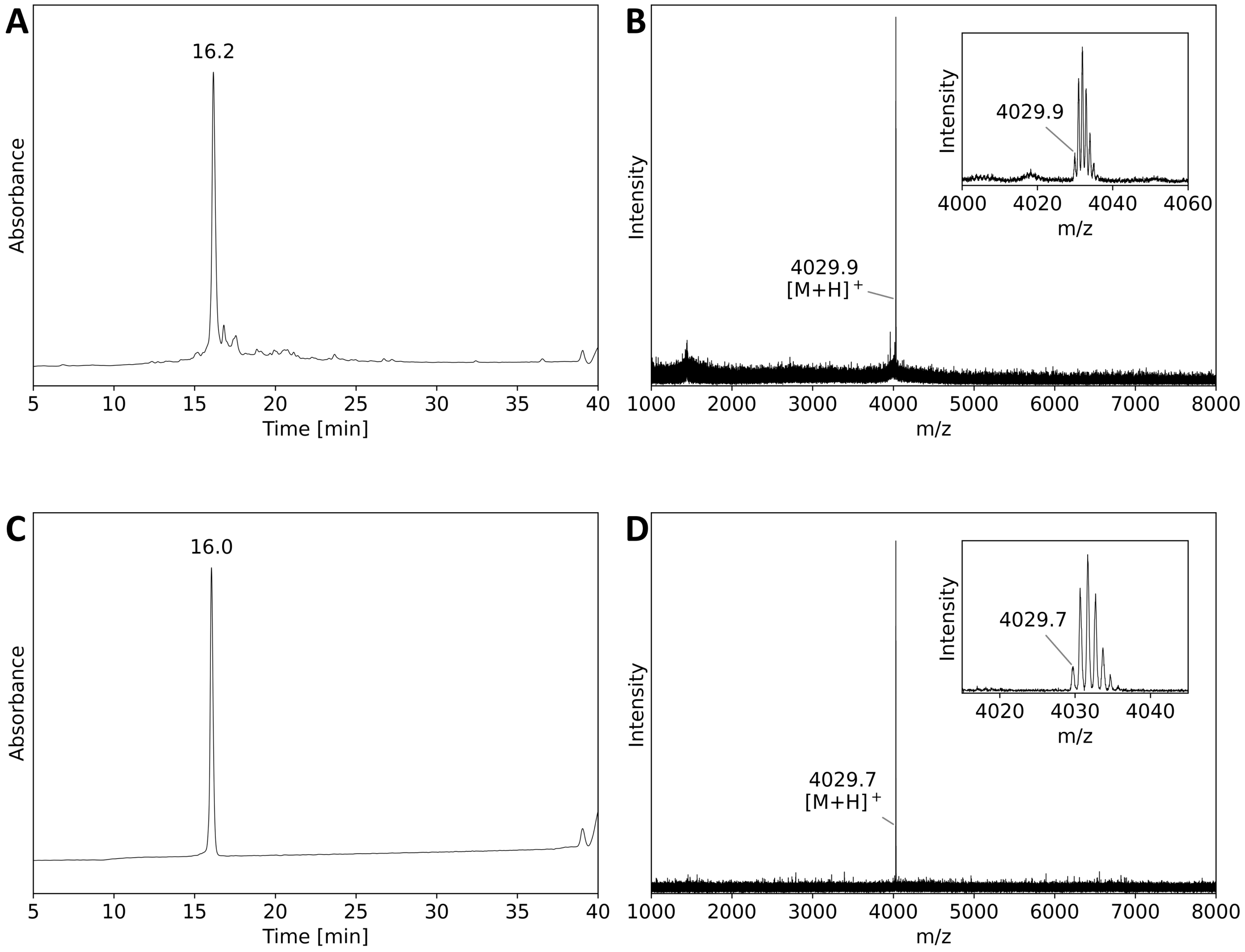

Figure S48: **P4-N15** A) Analytical HPLC (220 nm) of crude peptide. B) MALDI-TOF MS of crude peptide. C) Analytical HPLC (220 nm) of purified peptide. D) MALDI-TOF MS of purified peptide. Calculated mass: $[M+H]^+$ 4029.1. Calculated purity: 82 %. Calculated yield: >99 %.

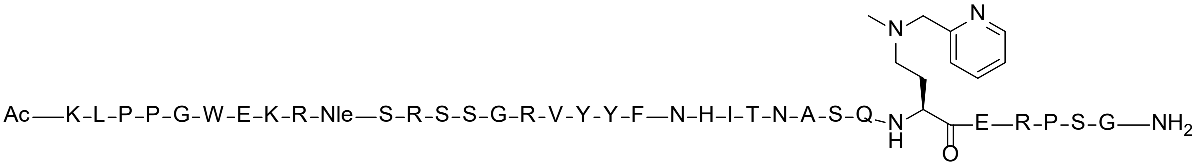

P4-N26
Chemical Formula: $C_{182}H_{279}N_{57}O_{50}$
Exact Mass: 4063.10
Molecular Weight: 4065.58

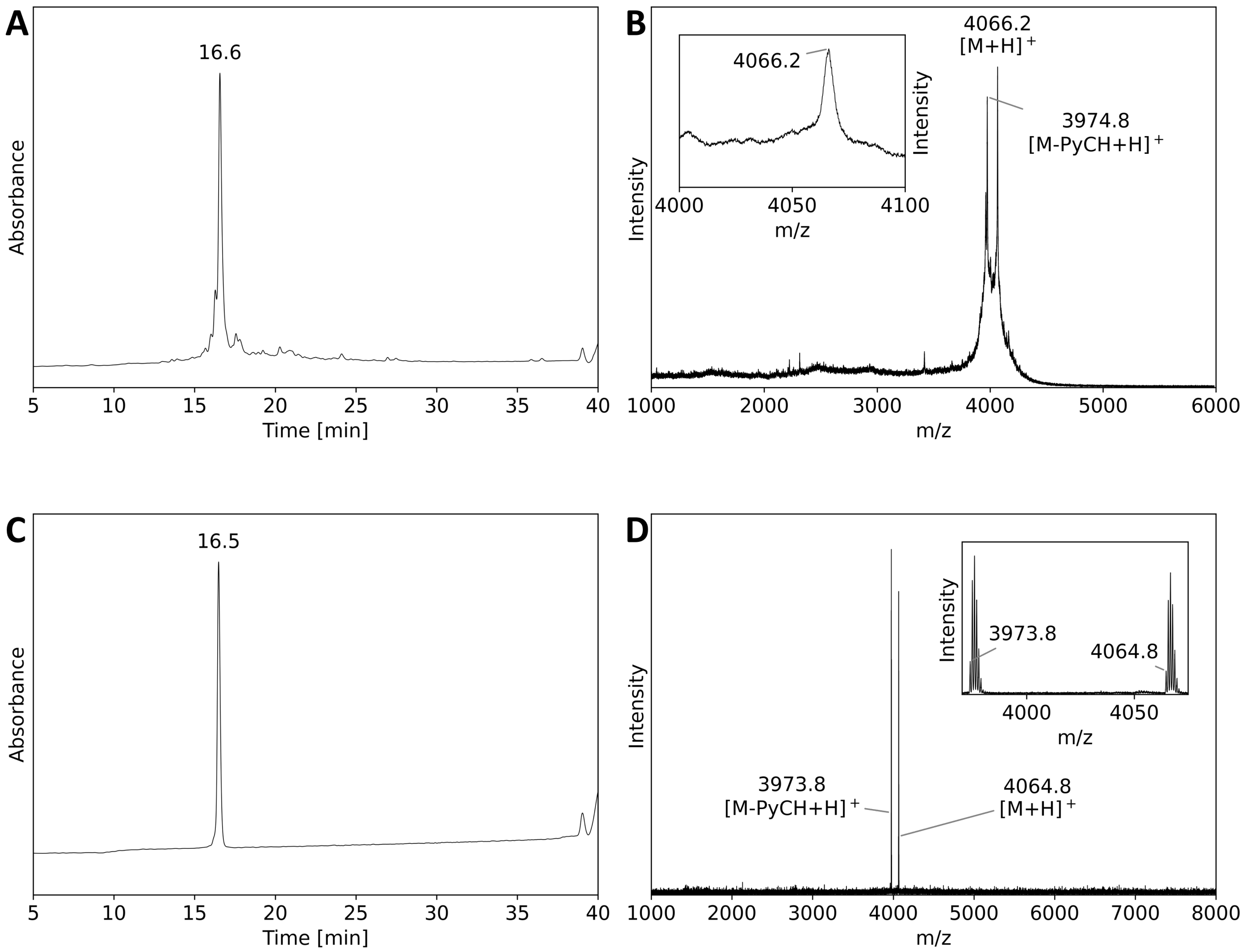

Figure S49: **P4-N26** A) Analytical HPLC (220 nm) of crude peptide. B) MALDI-TOF MS of crude peptide. C) Analytical HPLC (220 nm) of purified peptide. D) MALDI-TOF MS of purified peptide. Fragmentation of the picolyl substituent was observed in MALDI-TOF MS. Calculated mass: $[M\text{-}PyCH+H]^+$ 3974.1, $[M+H]^+$ 4064.1. Calculated purity: 73 %. Calculated yield: >99 %.

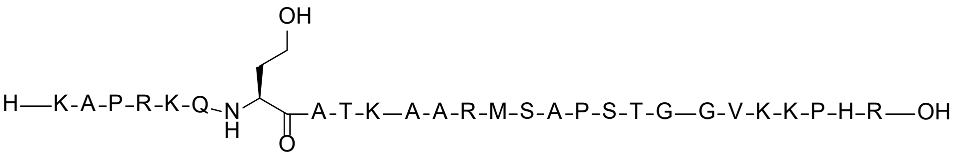

P5-OH

Chemical Formula: $C_{121}H_{214}N_{44}O_{34}S$

Exact Mass: 2859.61

Molecular Weight: 2861.38

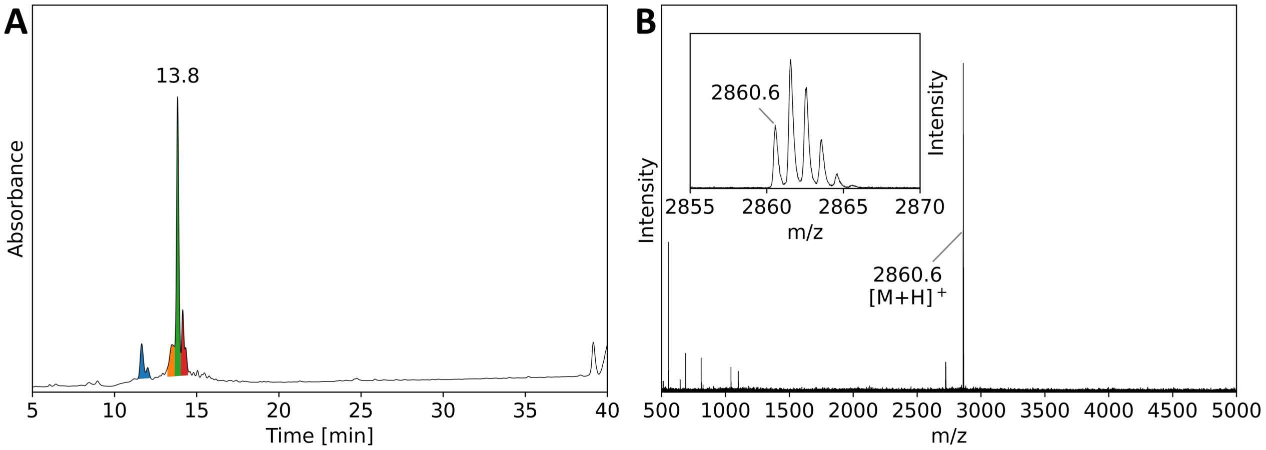

Figure S50: **P5-OH** A) Analytical HPLC (220 nm) of crude peptide. B) MALDI-TOF MS of crude peptide. Calculated mass: $[M+H]^+$ 2860.6. Integrated areas are highlighted. Calculated purity: 58 %.

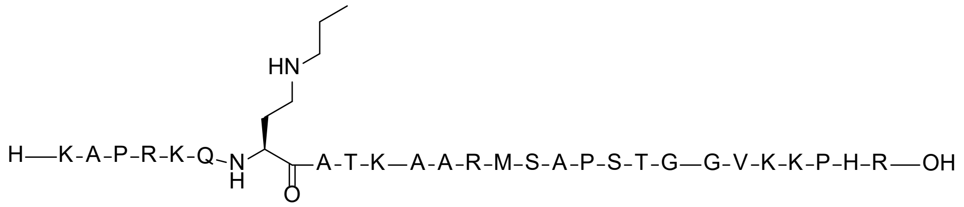

P5-N2
Chemical Formula: $C_{124}H_{221}N_{45}O_{33}S$
Exact Mass: 2900.67
Molecular Weight: 2902.47

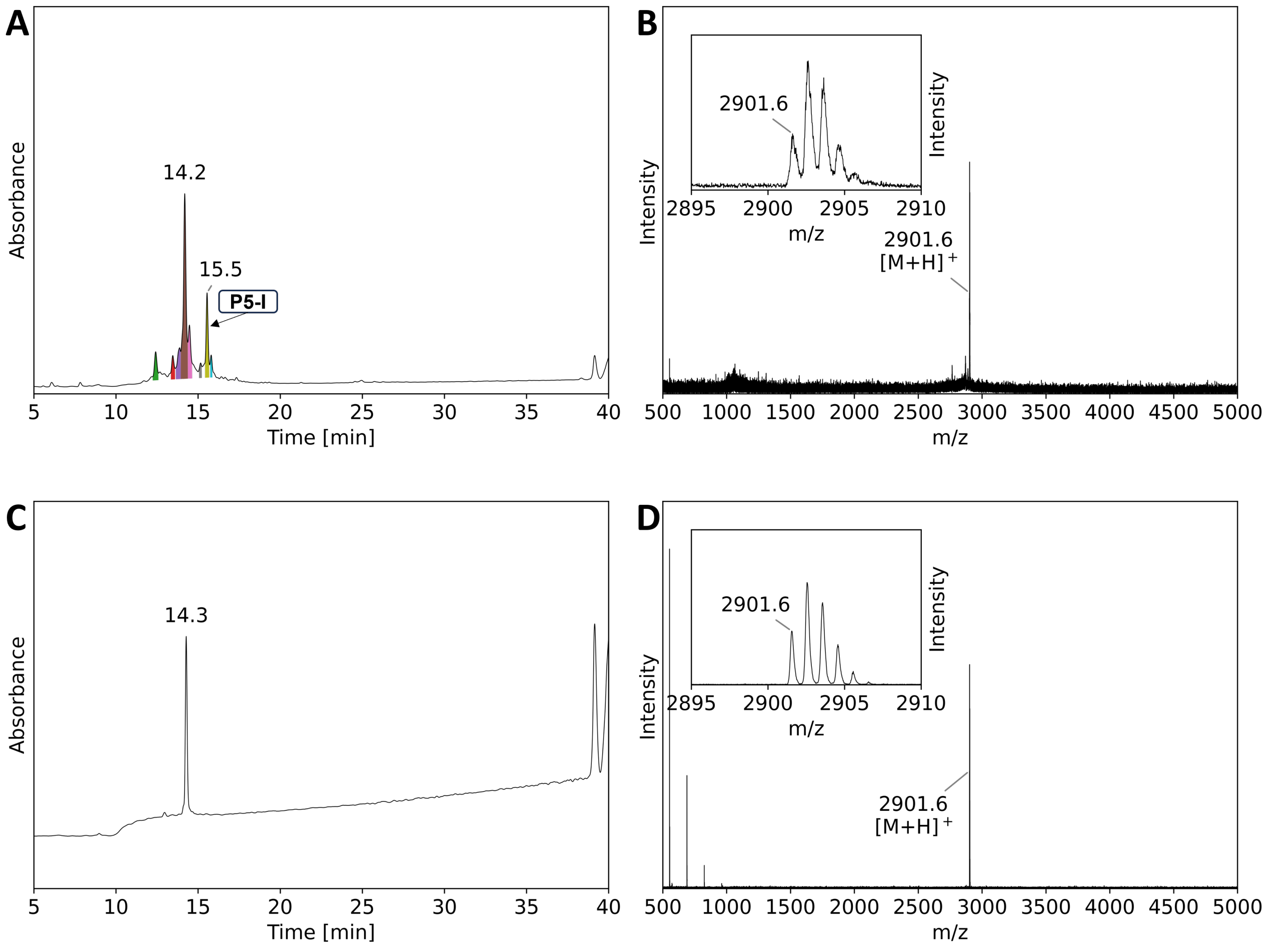

Figure S51: **P5-N2** A) Analytical HPLC (220 nm) of crude peptide. B) MALDI-TOF MS of crude peptide. C) Analytical HPLC (220 nm) of purified peptide. D) MALDI-TOF MS of purified peptide. Calculated mass: $[M+H]^+$ 2901.7. Integrated areas are highlighted. Calculated purity: 46 %. Calculated yield: 80 %.

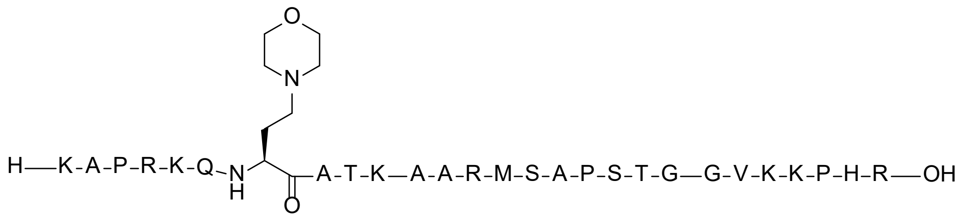

P5-N15

Chemical Formula: $C_{125}H_{221}N_{45}O_{34}S$
Exact Mass: 2928.67
Molecular Weight: 2930.48

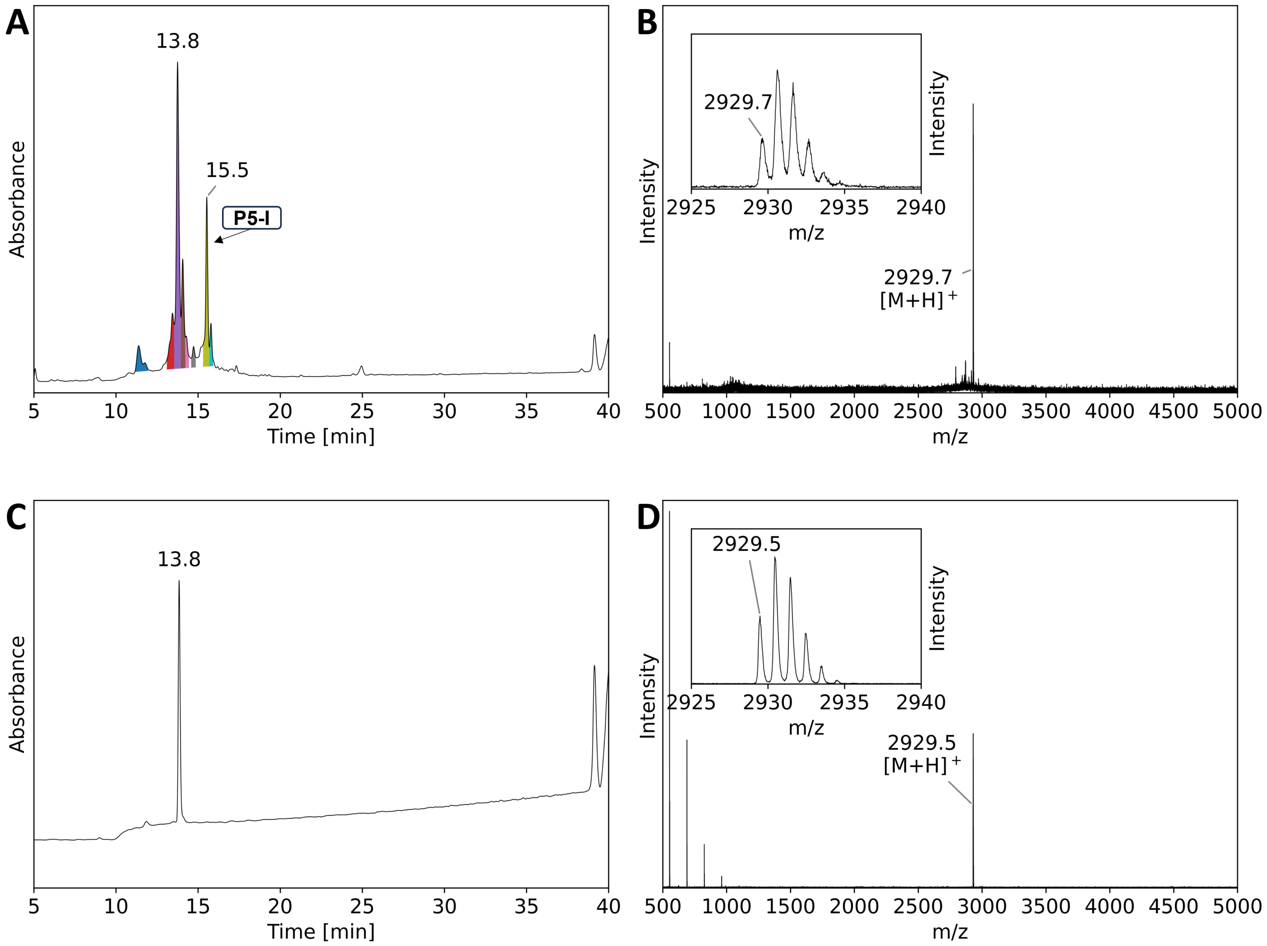

Figure S52: **P5-N15** A) Analytical HPLC (220 nm) of crude peptide. B) MALDI-TOF MS of crude peptide. C) Analytical HPLC (220 nm) of purified peptide. D) MALDI-TOF MS of purified peptide. Calculated mass: $[M+H]^+$ 2929.7. Integrated areas are highlighted. Calculated purity: 41 %. Calculated yield: 70 %.

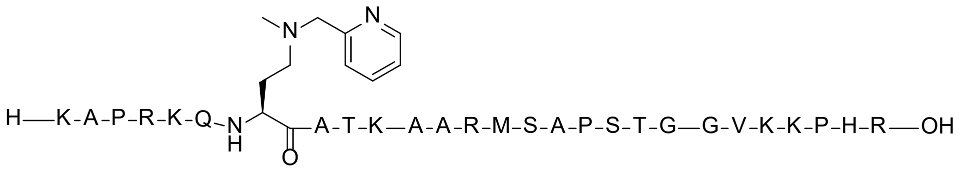

P5-N26

Chemical Formula: $C_{128}H_{222}N_{46}O_{33}S$
Exact Mass: 2963.68
Molecular Weight: 2965.53

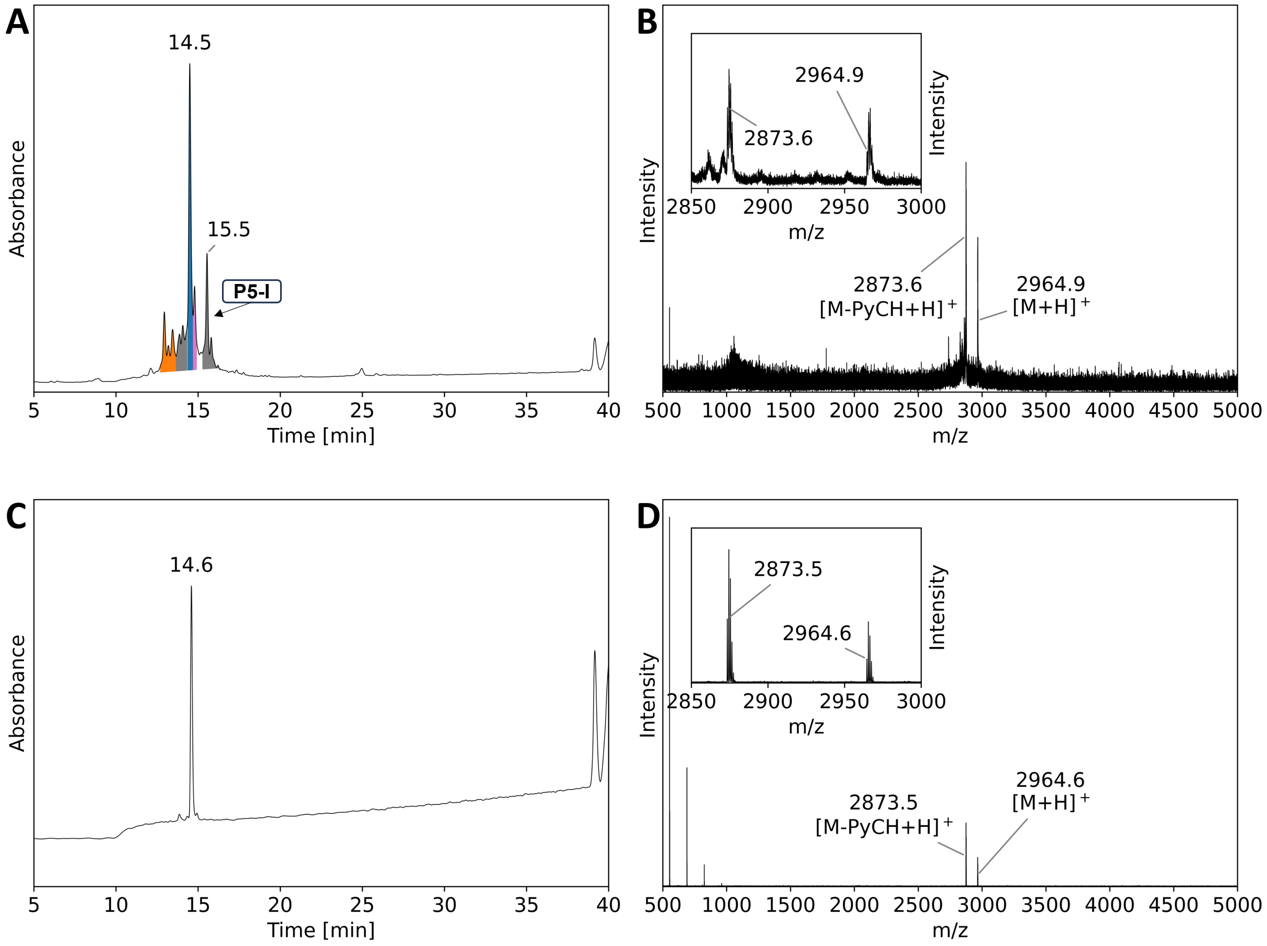

Figure S53: **P5-N26** A) Analytical HPLC (220 nm) of crude peptide. B) MALDI-TOF MS of crude peptide. C) Analytical HPLC (220 nm) of purified peptide. D) MALDI-TOF MS of purified peptide. Calculated mass: $[M\text{-}PyCH+H]^+$ 2873.7, $[M+H]^+$ 2964.7. Integrated areas are highlighted. Calculated purity: 47 %. Calculated yield: 80 %.

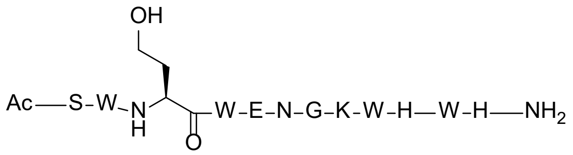

P6-OH

Chemical Formula: $C_{82}H_{99}N_{23}O_{18}$
Exact Mass: 1693.75
Molecular Weight: 1694.84

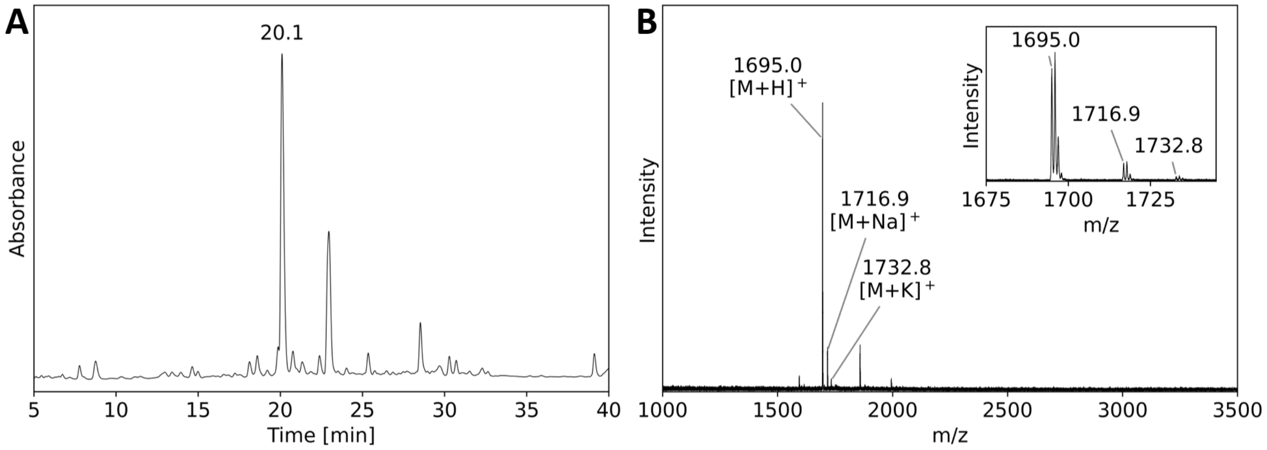

Figure S54: **P6-OH** A) Analytical HPLC (220 nm) of crude peptide. B) MALDI-TOF MS of crude peptide. Calculated mass: $[M+H]^+$ 1694.8, $[M+Na]^+$ 1716.8, $[M+K]^+$ 1732.7.

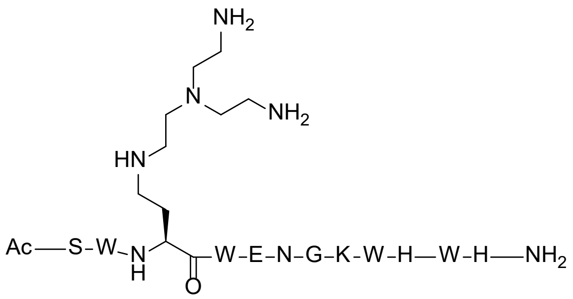

P6-N1

Chemical Formula: $C_{88}H_{115}N_{27}O_{17}$
Exact Mass: 1821.90
Molecular Weight: 1823.06

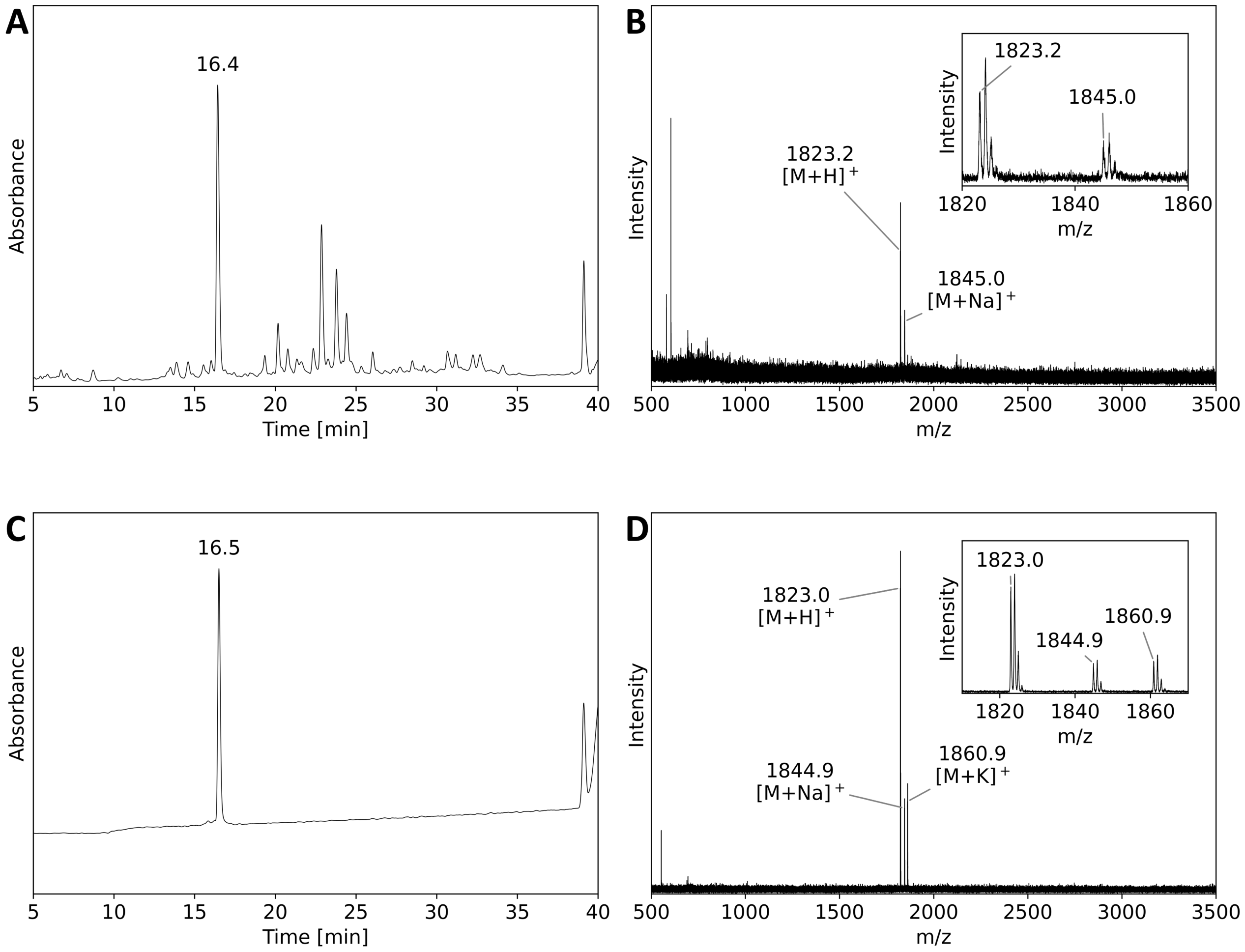

Figure S55: **P6-N1** A) Analytical HPLC (220 nm) of crude peptide. B) MALDI-TOF MS of crude peptide. C) Analytical HPLC (220 nm) of purified peptide. D) MALDI-TOF MS of purified peptide. Calculated mass: $[M+H]^+$ 1822.9, $[M+Na]^+$ 1844.9, $[M+K]^+$ 1860.9.

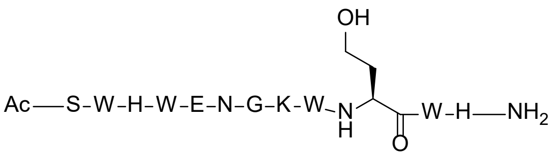

P7-OH

Chemical Formula: $C_{82}H_{99}N_{23}O_{18}$

Exact Mass: 1693.75

Molecular Weight: 1694.84

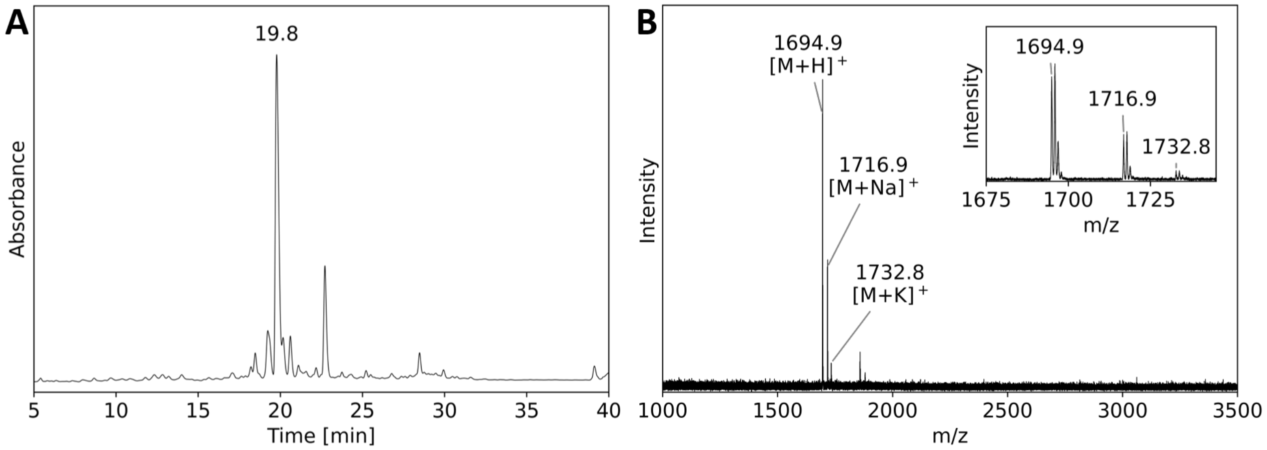

Figure S56: **P7-OH** A) Analytical HPLC (220 nm) of crude peptide. B) MALDI-TOF MS of crude peptide. Calculated mass: $[M+H]^+$ 1694.8, $[M+Na]^+$ 1716.7, $[M+K]^+$ 1732.7.

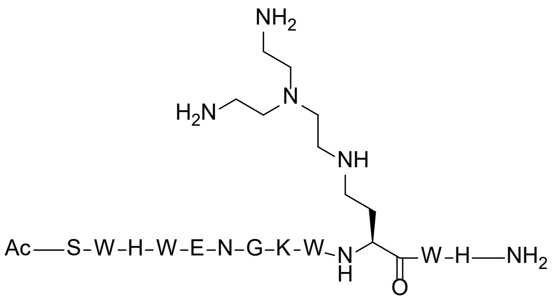

P7-N1
Chemical Formula: $C_{88}H_{115}N_{27}O_{17}$
Exact Mass: 1821.90
Molecular Weight: 1823.06

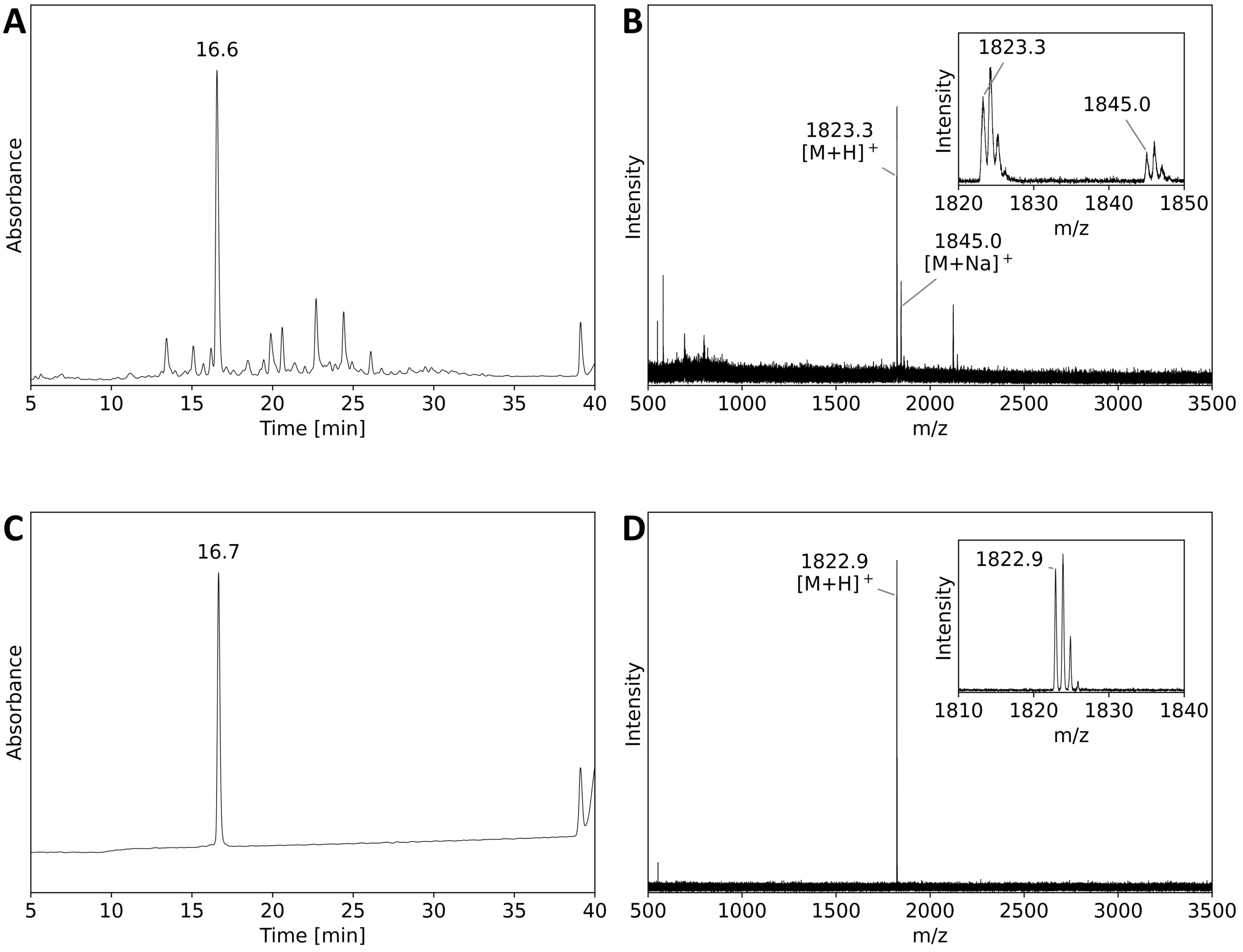

Figure S57: **P7-N1** A) Analytical HPLC (220 nm) of crude peptide. B) MALDI-TOF MS of crude peptide. C) Analytical HPLC (220 nm) of purified peptide. D) MALDI-TOF MS of purified peptide. Calculated mass: $[M+H]^+$ 1822.9, $[M+Na]^+$ 1844.9.

2 Experimental procedures

2.1 Reagents and solvents

Standard Fmoc-protected amino acids, Fmoc-Rink-Amide MBHA polystyrene resin, Oxyma® and *N,N'*-diisopropylcarbodiimide (DIC) were acquired from *Iris Biotech GmbH* (Marktredwitz, Germany). Boc-Gly-OH and Fmoc-Nle-OH were purchased from *Carbolution Chemicals GmbH* (St. Ingebert, Germany). *N,N'*-diisopropylethylamine (DIPEA), piperidine, MeCN (HPLC grade), trifluoroacetic acid (TFA, HPLC grade) and triisopropyl silane (TIPS) were acquired from *Merck KGaA* (Darmstadt, Germany). DMF (peptide synthesis grade) and formic acid (98+ %) was purchased from *Fisher Scientific* (Loughborough, United Kingdom). All other solvents and reagents were at least *reagent* grade quality and were acquired from *Carbolution Chemicals GmbH* (St. Ingebert, Germany), *BLD Pharmatech GmbH.* (Reinbek, Germany), *Carl Roth GmbH + Co. KG* (Karlsruhe, Germany), *Fisher Scientific* (Loughborough, United Kingdom), *Grüssing GmbH* (Filsum, Germany), *Honeywell* (Seelze, Germany), *Merck KGaA* (Darmstadt, Germany), *neoFroxx GmbH* (Einhausen, Germany), *Th. Geyer GmbH & Co. KG* (Renningen, Germany) and *VWR International* (Fontenay-sous-Bois, France). Reagents and solvents were used as received. Water was purified with a *Sartorius arium® mini* lab water system.

2.2 Software for data analysis and visualization

Unless stated otherwise, data were analyzed and visualized using *Python 3.9* with a *Tkinter 8.*6-based graphical user interface. Data analysis was conducted with the *NumPy 1.24.3*, and *SciPy 1.11.3* modules and data visualization was performed with the *matplotlib 3.8.0* module. Chemical structures were drawn with *ChemDraw 22.2.0*.

2.3 Peptide synthesis, functionalization and purification

2.3.1 Automated solid-phase peptide synthesis

Peptides **P1**, **P3**, **P4**, **P5**, **P6** and **P7** were synthesized at 0.1 mmol scale on a microwave-assisted peptide synthesizer (*CEM Liberty Blue*, see section 2.3.7 for detailed settings). Fmoc-Rink-Amide MBHA polystyrene resin with a loading density of 0.67 mmol/g was used as solid support for **P1**, **P3**, **P4**, **P6** and **P7**. **P5** was synthesized with preloaded Fmoc-Arg(Pbf)-Wang resin with a loading density of 0.322 mmol/g. Prior to synthesis, the resin was swollen in DMF for at least 10 min. Fmoc-protected amino acids were used as 0.2 M solution in DMF. *N,N'*-Diisopropylcarbodiimide (DIC) dissolved in DMF (0.5 M) was used as activator, and Oxyma® solution in DMF (1.0 M) plus 0.1 M *N,N*-diisopropylethylamine (DIPEA) was used as the activator base. A solution of piperidine/DMF 1:4 *v/v* was used to remove the Fmoc protecting group. Post synthesis, the peptide was transferred to a 10 mL syringe reactor with a PE frit, washed with DMF (5 x 5 mL) and DCM (10 x 5 mL) and dried under reduced pressure in a desiccator for at least 10 minutes. For *N*-terminal acetylation of peptides **P3**, **P4**, **P6**, and **P7**, the peptide resin was allowed to swell in DMF (5 mL) for at least 5 minutes and subsequently treated with acetic anhydride (0.5 mL) in pyridine (4.5 mL) for 10 min. The peptide resin was washed with DMF (3 x 5 mL) and DCM (10 x 5 mL) and dried under reduced pressure in a desiccator. The mass of the resin was determined and it was assumed that this corresponds to 0.1 mmol peptide.

2.3.2 Manual solid-phase peptide synthesis

Peptide **P2** was synthesized at 0.1 mmol scale by manual solid-phase peptide synthesis in a 20 mL syringe reactor equipped with a PE frit. Fmoc-Rink-Amide MBHA polystyrene resin with a loading density of 0.67 mmol/g was used as solid support. Prior to synthesis, the resin was swollen in DMF for at least 10 min. The standard cycle for peptide synthesis consists of 1) deprotection, 2) wash, 3) coupling and 4) wash.

1) **Deprotection**: To remove the Fmoc protecting group, the resin was treated with a solution of 20 % piperidine in DMF (5 mL) and the syringe reactor was shaken for 20 min.
2) **Wash:** The solution was discarded and the resin was washed with DMF (3 × 5 mL), DCM (3 × 5 mL) and DMF (3 × 5 mL).
3) **Coupling:** The protected amino acid (300 µmol, 3.00 eq.) was dissolved in DMF (5 mL), before DIPEA (104 µL, 600 µmol, 6.00 eq.) and PyBOP (156 mg, 300 µmol, 3.00 eq.) were added. After one minute of incubation, the resin was treated with the mixture and the syringe reactor was shaken for one hour.

Acetylation was performed as described in the previous chapter. The peptide resin was washed with DMF (3 x 5 mL) and DCM (10 x 5 mL) and dried under reduced pressure in a desiccator. The mass of the resin was determined and it was assumed that this corresponds to 0.1 mmol peptide.

2.3.3 Cleavage and isolation of peptides

Cleavage of the peptide from the resin and final deprotection were carried out in trifluoroacetic acid (TFA) containing triisopropyl silane (TIPS) and water. Dry resin with 5 µmol peptide was placed into a 2 mL syringe reactor with a PE frit and incubated with a cleavage cocktail consisting of 0.48 mL TFA, 25 µL TIPS and 25 µL water for 3 h. The solution was collected and the resin was washed with TFA (2 x 0.5 mL). The TFA was removed in a stream of nitrogen and the peptide was precipitated from ice-cold Et_2O (1 mL). The peptide was isolated by centrifugation. The pellet was washed with Et_2O (2 x 1 mL) and dissolved in 1 mL 20 % Buffer B (MeCN + 0.1 %) in Buffer A (water + 0.1 %).

2.3.4 Functionalization on solid phase

Resin loaded with 50 µmol peptide was placed in a 20 mL syringe reactor with a PE frit. To remove the Dmt protecting group, the resin was washed with 1 % TFA in DCM (5 x 10 mL) and DCM (10 x 10 mL). The peptide was directly iodinated afterwards to prevent depsipeptide formation.[1] The resin was not dried and used directly in the next step.

For iodination, methyltriphenoxyphosphonium iodide (1.15 g, 2.5 mmol, 50 eq.) was dissolved in dry DMF (5 mL) in a nitrogen-flushed desiccator. The resin was incubated with the iodination reagent for 20 min under gentle shaking. The solution was removed and the resin was washed with DMF (5 x 10 mL) and DCM (10 x 10 mL) and dried under reduced pressure. The resin with iodinated peptide was further divided into 10 parts referring to 5 µmol peptide.

For nucleophilic substitution, the nucleophile (250 µmol, 50 eq.) was dissolved in dry MeCN (0.25 mL) in a nitrogen-flushed desiccator. Resin loaded with 5 µmol iodinated peptide was placed in a 2 mL syringe reactor with a PE frit. The nucleophile was added to the resin and the syringe was shaken for 16 h at room temperature. After reaction, the solution was removed and the resin was washed with MeCN (5 x 1 mL) and DCM (10 x 1 mL) and dried under reduced pressure. **P2** was functionalized at 0.1 mmol scale with linearly upscaled reagents. For the reaction with **N11-HCl** and **N22**, the solvent was exchanged with dry DMF (0.25 mL) and after reaction, the solution was removed and the resin was washed with DMF (5 x 1 mL) and DCM (10 x 1 mL) and dried under reduced pressure. Reactions with 2.5 M amine solution were performed by adding a solution of amine (2.5 mmol, 500 eq.) in 1 mL solvent to the resin, except for (diphenylphosphaneyl)ethan-1-amine (**N10**), which was prepared by dissolving **N10** (1.25 mmol, 250 eq.) in 0.5 mL solvent.

Cleavage and final deprotection was performed as described above.

Reactions listed in Table 2 were prepared similarly, whereby for entry 6, tren (250 µmol, 50 eq.) was dissolved in 1 mL dry MeCN resulting in a concentration of 0.25 M. For entry 7, tren (2.5 mmol, 500 eq.) was dissolved in 1 mL dry MeCN resulting in a concentration of 2.5 M. For entry 8, the reaction was carried out in a CEM Discovery SP microwave oven at 95 °C and 300 W with stirring for 90 s. For entry 9 and 10, 2.6 µL formic acid (37.5 µmol, 7.5 eq.) was added to the reaction mixture, whereby in the case of entry 10 the solvent was changed to dry DMF.

2.3.5 High-performance liquid chromatography (HPLC)

The peptides were purified by reversed-phase HPLC on a *VDS optilab VDSpher® PUR 100 C18-SE* (250 mm x 10 mm, 100 Å, 5 µm) column at a flow rate of 3 mL/min at 50 °C. A *Jasco* chromatography system with a *PU-4180* pump, a *CO-4060* column thermostat and a *UV-4070* detector was used. Analytical HPLC was performed using a *Hitachi Primaide* chromatography system containing a *1110 Pump*, a *1210 auto sampler*, *1310 column oven* and a *1430 diode array detector*. A *VDS optilab VDSpher® PUR 100 C18-SE* (250 mm x 4.6 mm, 100 Å, 5 µm) column and a flow rate of 1 mL/min at 50 °C were used. Preparative chromatograms were monitored at 220 nm and 280 nm. Analytical chromatograms were monitored from 190 to 400 nm. Fractions containing pure peptide were identified by analytical HPLC and MALDI-TOF MS, pooled and lyophilized. As solvent system, water + 0.1 % TFA (Buffer A) and MeCN + 0.1 % TFA (Buffer B) was used. Only for preparative HPLC of **P5**, the solvent system was changed to water + 0.1 % HCl (Buffer A) and

MeCN + 0.1 % HCl (Buffer B). The gradients are displayed in Table S1. Prior to injection, peptide solutions were filtered with H-PTFE syringe filters (0.22 µm pore size) from *VWR*.

Table S1: Gradients used for the purification and characterization of peptides **P1** to **P7**. The percentages of Buffer B in Buffer A (*v*/*v*) are given.

preparative	Isocratic 1	linear gradient	Isocratic 2	Isocratic 3
**time / min**	0 → 8	8 → 38	39 → 44	45 → 50
**P1 and P3**	10 %	10 – 40 %	100 %	10 %
**P2**	5 %	5 – 35 %	100 %	5 %
**P4**	20 %	20 – 45 %	100 %	20 %
**P5**	5 %	5 – 25 %	100 %	5 %
**P6 and P7**	20 %	20 – 40 %	100 %	20 %
**analytical**				
**time / min**	0 → 5	5 → 35	36 → 41	42 → 50
**P1, P3 and P5**	10 %	10 – 40 %	100 %	10 %
**P2**	5 %	5 – 35 %	100 %	5 %
**P4**	20 %	20 – 45 %	100 %	20 %
**P6 and P7**	20 %	20 – 40 %	100 %	20 %

2.3.6 Mass spectrometry

Electrospray ionization (ESI) was performed on an ion cyclotron resonance *Bruker microTOF-Q II*.
Matrix-assisted laser desorption ionization time-of-flight mass spectrometry (MALDI-TOF MS) was performed on a *Bruker Autoflex Speed*. 1 µl of the matrix 2,5-dihydroxybenzoic acid (20 mg/mL in 3:7 MeCN/water + 0.1 % TFA) was mixed with 1 µl of the sample, pipetted onto the target plate and air dried.

2.3.7 Settings of the peptide synthesizer

The following Tables (S2-S14) contain the detailed settings for the microwave-assisted peptide syntheses performed in this study using the *CEM LibertyBlue* peptide synthesizer.

Table S2: Settings for **Microwave Methods**. Mixing of the reaction mixture was performed by bubbling nitrogen through the reaction vessel frit (bubble for 2 s, off for 3 s).

Microwave Method	Temperature / °C	Power / W	Hold Time / s	DeltaT / °C
Standard deprotection	75	155	15	2
	90	30	50	1
Coupling	75	170	15	2
	90	30	110	1
50 °C 10 min Coupling	25	0	120	2
	50	35	480	1

Table S3: Settings for **Resin Swelling**.

	Cycle Steps	Parameter values
1	Swell Resin	Main solvent volume: 15 mL, Time: 300 s

Table S4: Settings for **Single Coupling**.

	Cycle Steps	Parameter values
1	Deprotection	Reaction Method: Standard Deprotection, Deprotection Volume: 4 mL
2	Wash	Volume: 4 mL, Drain Time: 10 s
3	Wash	Volume: 4 mL, Drain Time: 5 s
4	Wash	Volume: 4 mL, Drain Time: 5 s
5	Wash	Volume: 4 mL, Drain Time: 5 s
6	Coupling	Reaction Method: Standard coupling, Amino Acid: from method, Amino Acid Volume: 2.5 mL, Activator Bottle Position: PositionACT, Activator Volume: 2 mL, Activator Base Position: PositionACTB, Activator Base Volume: 1 mL, Delayed Reagent Time: 0 s, Delayed Reagent Bottle Position: PositionACTB, Delayed Reagent Volume: 0 mL, Manifold Wash Volume: 2 mL
7	Wash	Volume: 4 mL, Drain Time: 10 s
8	Wash	Volume: 4 mL, Drain Time: 5 s

Table S5. Settings for **Single 50 °C Coupling**.

	Cycle Steps	Parameter values
1	Deprotection	Reaction Method: Standard Deprotection, Deprotection Volume: 4 mL
2	Wash	Volume: 4 mL, Drain Time: 10 s
3	Wash	Volume: 4 mL, Drain Time: 5 s
4	Wash	Volume: 4 mL, Drain Time: 5 s
5	Wash	Volume: 4 mL, Drain Time: 5 s
6	Coupling	Reaction Method: 50 °C 10 min coupling, Amino Acid: from method, Amino Acid Volume: 2.5 mL, Activator Bottle Position: PositionACT, Activator Volume: 2 mL, Activator Base Position: PositionACTB, Activator Base Volume: 1 mL, Delayed Reagent Time: 0 s, Delayed Reagent Bottle Position: PositionACTB, Delayed Reagent Volume: 0 mL, Manifold Wash Volume: 2 mL
7	Wash	Volume: 4 mL, Drain Time: 10 s
8	Wash	Volume: 4 mL, Drain Time: 5 s

Table S6. Settings for **First Single 50 °C Coupling**.

	Cycle Steps	Parameter values
1	Deprotection	Reaction Method: Standard Deprotection, Deprotection Volume: 4 mL
2	Deprotection	Reaction Method: Standard Deprotection, Deprotection Volume: 4 mL
3	Wash	Volume: 4 mL, Drain Time: 10 s
4	Wash	Volume: 4 mL, Drain Time: 5 s
5	Wash	Volume: 4 mL, Drain Time: 5 s
6	Wash	Volume: 4 mL, Drain Time: 5 s
7	Coupling	Reaction Method: 50 °C 10 min coupling, Amino Acid: from method, Amino Acid Volume: 2.5 mL, Activator Bottle Position: PositionACT, Activator Volume: 2 mL, Activator Base Position: PositionACTB, Activator Base Volume: 1 mL, Delayed Reagent Time: 0 s, Delayed Reagent Bottle Position: PositionACTB, Delayed Reagent Volume: 0 mL, Manifold Wash Volume: 2 mL
8	Wash	Volume: 4 mL, Drain Time: 10 s
9	Wash	Volume: 4 mL, Drain Time: 5 s

Table S7: Settings for **Double Coupling**.

	Cycle Steps	Parameter values
1	Deprotection	Reaction Method: Standard Deprotection, Deprotection Volume: 4 mL
2	Wash	Volume: 4 mL, Drain Time: 10 s
3	Wash	Volume: 4 mL, Drain Time: 5 s
4	Wash	Volume: 4 mL, Drain Time: 5 s
5	Wash	Volume: 4 mL, Drain Time: 5 s
6	Coupling	Reaction Method: Standard coupling, Amino Acid: from method, Amino Acid Volume: 2.5 mL, Activator Bottle Position: Position ACT, Activator Volume: 2 mL, Activator Base Position: Position ACTB, Activator Base Volume: 1 mL, Delayed Reagent Time: 0 s, Delayed Reagent Bottle Position: PositionACTB, Delayed Reagent Volume: 0 mL, Manifold Wash Volume: 2 mL
7	Wash	Volume: 4 mL, Drain Time: 5 s
8	Coupling	see step 6
9	Wash	Volume: 4 mL, Drain Time: 10 s
10	Wash	Volume: 4 mL, Drain Time: 5 s

Table S8: Settings for **Double Coupling wash**.

	Cycle Steps	Parameter values
1	Deprotection	Reaction Method: Standard Deprotection, Deprotection Volume: 4 mL
2	Wash	Volume: 4 mL, Drain Time: 10 s
3	Wash	Volume: 4 mL, Drain Time: 5 s
4	Wash	Volume: 4 mL, Drain Time: 5 s
5	Wash	Volume: 4 mL, Drain Time: 5 s
6	Wash	Volume: 4 mL, Drain Time: 5 s
7	Wash	Volume: 4 mL, Drain Time: 10 s
8	Coupling	Reaction Method: Standard coupling, Amino Acid: from method, Amino Acid Volume: 2.5 mL, Activator Bottle Position: Position ACT, Activator Volume: 2 mL, Activator Base Position: Position ACTB, Activator Base Volume: 1 mL, Delayed Reagent Time: 0 s, Delayed Reagent Bottle Position: PositionACTB, Delayed Reagent Volume: 0 mL, Manifold Wash Volume: 2 mL
9	Wash	Volume: 4 mL, Drain Time: 10 s
10	Wash	Volume: 4 mL, Drain Time: 10 s
11	Coupling	see step 8
12	Wash	Volume: 4 mL, Drain Time: 10 s
13	Wash	Volume: 4 mL, Drain Time: 5 s
14	Wash	Volume: 4 mL, Drain Time: 5 s
15	Wash	Volume: 4 mL, Drain Time: 5 s

Table S9: Settings for **Final Deprotection**.

	Cycle Steps	Parameter values
1	Deprotection	Reaction Method: Standard Deprotection, Deprotection Volume: 4 mL
2	Wash	Volume: 4 mL, Drain Time: 5 s
3	Wash	Volume: 4 mL, Drain Time: 5 s
4	Wash	Volume: 4 mL, Drain Time: 5 s
5	Wash	Volume: 4 mL, Drain Time: 5 s

Table S10: Settings for the synthesis of **P1**.

	Step	Used Cycles (optimized conditions)
**1**	Resin swelling	Resin swelling
**2**	E	Single Coupling
**3**	L	Single Coupling
**4**	R	Double Coupling wash
**5**	Q	Single Coupling
**6**	A	Single Coupling
**7**	I	Single Coupling
**8**	E	Single Coupling
**9**	A	Single Coupling
**10**	K	Single Coupling
**11**	Hse	Single Coupling
**12**	P	Single Coupling
**13**	Boc-Gly	Double Coupling wash

Table S11: Settings for the synthesis of **P3**.

	Step	Used Cycles (optimized conditions)
**1**	Resin swelling	Resin swelling
**2**	H	Single 50 °C Coupling
**3**	T	Single Coupling
**4**	R	Double Coupling
**5**	Q	Single Coupling
**6**	H	Single 50 °C Coupling
**7**	K	Single Coupling
**8**	Q	Single Coupling
**9**	L	Single Coupling
**10**	N	Single Coupling
**11**	S	Single Coupling
**12**	S	Single Coupling
**13**	Q	Single Coupling
**14**	S	Single Coupling
**15**	F	Single Coupling
**16**	S	Single Coupling
**17**	K	Single Coupling
**18**	G	Double Coupling
**19**	C	Single Coupling
**20**	E	Single Coupling
**21**	P	Single Coupling
**22**	C	Single Coupling
**23**	K	Single Coupling
**24**	Y	Single Coupling
**25**	P	Single Coupling
**26**	Hse	Single Coupling
**27**	Final Deprotection	Final Deprotection

Table S12: Settings for the synthesis of **P4**.

	Step	Used Cycles (optimized conditions)
**1**	Resin swelling	Resin swelling
**2**	G	Double Coupling wash
**3**	S	Single Coupling
**4**	P	Single Coupling
**5**	R	Double Coupling
**6**	E	Single Coupling
**7**	Hse	Single Coupling
**8**	Q	Single Coupling
**9**	S	Single Coupling
**10**	A	Single Coupling
**11**	N	Single Coupling
**12**	T	Single Coupling
**13**	I	Single Coupling
**14**	H	Single 50 °C Coupling
**15**	N	Single Coupling
**16**	F	Single Coupling
**17**	Y	Single Coupling
**18**	Y	Single Coupling
**19**	V	Single Coupling
**20**	R	Double Coupling
**21**	G	Double Coupling wash
**22**	S	Single Coupling
**23**	S	Single Coupling
**24**	R	Double Coupling
**25**	S	Single Coupling
**26**	Nle	Single Coupling
**27**	R	Double Coupling
**28**	K	Single Coupling
**29**	E	Single Coupling
**30**	W	Single Coupling
**31**	G	Double Coupling wash
**32**	P	Single Coupling
**33**	P	Single Coupling
**34**	L	Single Coupling
**35**	K	Single Coupling
**36**	Final deprotection	Final Deprotection

Table S13: Settings for the synthesis of **P5**.

	Step	Used Cycles (optimized conditions)
**1**	Resin swelling	Resin swelling
**2**	H	Single 50 °C Coupling
**3**	P	Single Coupling
**4**	K	Single Coupling
**5**	K	Single Coupling
**6**	V	Single Coupling
**7**	G	Double Coupling wash
**8**	G	Double Coupling wash
**9**	T	Single Coupling
**10**	S	Single Coupling
**11**	P	Single Coupling
**12**	A	Single Coupling
**13**	S	Single Coupling
**14**	M	Single Coupling
**15**	R	Double Coupling
**16**	A	Single Coupling
**17**	A	Single Coupling
**18**	K	Single Coupling
**19**	T	Single Coupling
**20**	A	Single Coupling
**21**	Hse	Single Coupling
**22**	Q	Single Coupling
**23**	K	Single Coupling
**24**	R	Double Coupling
**25**	P	Single Coupling
**26**	A	Single Coupling
**27**	Boc-Lys(Boc)	Single Coupling

Table S14: Settings for the synthesis of **P6**.

	Step	Used Cycles (optimized conditions)
**1**	Resin swelling	Resin swelling
**2**	H	First Single 50 °C Coupling
**3**	W	Single Coupling
**4**	H	Single 50 °C Coupling
**5**	W	Single Coupling
**6**	K	Single Coupling
**7**	G	Double Coupling wash
**8**	N	Single Coupling
**9**	E	Single Coupling
**10**	W	Single Coupling
**11**	Hse	Single Coupling
**12**	W	Single Coupling
**13**	S	Single Coupling
**21**	Final deprotection	Final Deprotection

Table S15: Settings for the synthesis of **P7**.

	Step	Used Cycles (optimized conditions)
**1**	Resin swelling	Resin swelling
**2**	H	First Single 50 °C Coupling
**3**	W	Single Coupling
**4**	Hse	Single Coupling
**5**	W	Single Coupling
**6**	K	Single Coupling
**7**	G	Double Coupling wash
**8**	N	Single Coupling
**9**	E	Single Coupling
**10**	W	Single Coupling
**11**	H	Single 50 °C Coupling
**12**	W	Single Coupling
**13**	S	Single Coupling
**14**	Final deprotection	Final Deprotection

2.4 CD spectroscopy

CD spectroscopy was carried out using a *JASCO J-1700* spectrometer with a *JASCO PTC-510* Peltier element and a 1 mm quartz cuvette by *STARNA*. The concentrations of **P6** and **P7** were determined spectroscopically by measurement of the tryptophan absorbance (ε_{280} = 22,000 $\mathrm{M}^{-1}\mathrm{cm}^{-1}$ = 4 x 5,500 $\mathrm{M}^{-1}\mathrm{cm}^{-1}$)[3] of 1 µL of dissolved sample at 280 nm with an *IMPLEN NP80* nanophotometer. Samples were prepared in MOPS buffer (10 mM, pH 7.4, 150 mM NaCl) with a 50 µM peptide concentration and measured after at least one hour of incubation. Metals were always added last. CD spectra were measured with 200 µL of sample at 20 °C from 260 to 190 nm. The settings used are: data pitch: 0.1 nm; scanning speed: 100 nm/min; scanning mode: continuous; D.I.T.: 2 s; bandwidth: 1.0 nm; accumulations: 10. The measured ellipticity Θ_{meas} [mdeg] was converted to the mean residue ellipticity $\Theta \left[\frac{\mathrm{deg \cdot cm^2}}{\mathrm{dmol \cdot res}}\right]$ using **eq. 1**:[4]

$$\Theta = \frac{\Theta_{meas} - \Theta_{meas,blank}}{n \cdot c \cdot l}$$ **(eq. 1)**[4]

n: Number of peptide bonds; c: Peptide concentration in mol/l; l: Pathlength in mm.

Thermal denaturation profiles were measured at the maximum of the exciton signal in the CD spectrum (228 nm) at a temperature range of 0 °C to 98 °C with a gradient of 1 °C/min. The baselines were assumed to be constant. Data were fitted using **eq. 2**:[5]

$$\Theta(T) = A + \frac{B - A}{1 + \exp\left(-\Delta H \cdot \left(1 - \frac{T + 273.15\ \mathrm{K}}{T_\mathrm{m} + 273.15\ \mathrm{K}}\right) \Big/ R \cdot (T + 273.15\ \mathrm{K}) \right)}$$ **(eq. 2)**[5]

A,B: Fitting parameters; ΔH: Molar enthalpy; T: Temperature; T_m: Melting Temperature, R: Universal gas constant.

2.5 NMR spectroscopy

Nuclear magnetic resonance (NMR) spectroscopy was performed on a *Bruker Avance III 400* spectrometer at 400 MHz. The measured ^{1}H-NMR spectra were referenced to the residual protons of the deuterated solvent (δ = 2.50 ppm for DMSO-d_6).
Spectra processing and analysis was performed using *TopSpin 4.1.4*. Signal multiplicities were abbreviated with s (singlet), d (doublet), t (triplet), q (quartet), dt (doublet of triplets), br (broad signal), m (multiplet).

2.6 Thin layer and column chromatography

Thin layer chromatography (TLC) was performed to monitor the reaction. *Merck TLC Silica gel 60 F_{254}* on aluminium foil was used. The spots were visualized with UV-light (254 nm) and, in case of Fmoc-Hse(Dmt)-OH, by staining with diluted sulfuric acid.
Column chromatography was performed with *Merck Silica gel 60* (0.040 to 0.063 mm). The collected fractions were analysed with TLC.

3 Organic Synthesis

3.1 Fmoc-Hse-OH

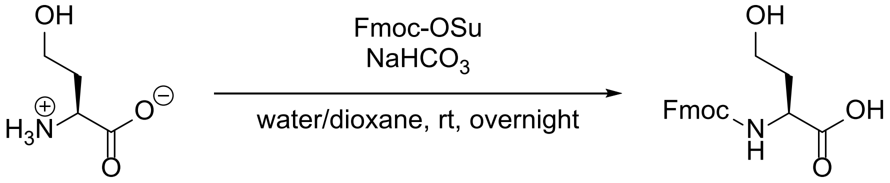

H-Hse-OH **Fmoc-Hse-OH**

Homoserine (2.00 g, 16.8 mmol, 1.00 eq.), Fmoc-OSu (5.66 g, 16.8 mmol, 1.00 eq.) and $NaHCO_3$ (2.75 g, 32.7 mmol, 1.95 eq.) were dissolved in water (55 mL) and dioxane (65 mL). The solution was stirred overnight at rt. Dioxane was removed under reduced pressure. The mixture was transferred into a separating funnel with water (200 mL), 5 % $NaHCO_3$ in water (150 mL) and DCM (150 mL). After removing of the organic phase, the aqueous phase was washed with diethyl ether (3 x 200 mL). The organic phases were discarded. Citric acid (50 g) was added to the aqueous phase. The aqueous phase was extracted with EtOAc (3 x 150 mL). The combined EtOAc phases were washed with brine (100 mL), dried over Na_2SO_4 and filtered. The solvent was removed under reduced pressure to give an oil, which was dissolved in EtOAc (25 mL). Crystallisation occurred overnight at −21 °C. The colourless solid was filtered and washed with ice-cold EtOAc. The product was dried in a desiccator under reduced pressure. The mother liquor was evaporated under reduced pressure and recrystallization was performed as stated.

Yield: 4.83 g, 14.2 mmol, 84 %.

TLC: R_f (DCM/MeOH/HCOOH 10:1:0.1) = 0.44.

^{1}H NMR (400 MHz, DMSO-d$_6$) δ = 12.53 (s, 1H, COOH), 7.89 (d, 2H, J = 7.5 Hz, H_{ar}), 7.73 (dd, 2H, J = 7.4, 1.1 Hz, H_{ar}), 7.59 (d, 1H, J = 8.0 Hz, NH), 7.42 (td, J = 7.5, 1.2 Hz, 2H, H_{ar}), 7.33 (t, 2H, J = 7.5 Hz, H_{ar}), 4.58 (s, 1H, OH), 4.30 - 4.19 (m, 3H, CH-CH_2 Fmoc), 4.13 - 4.06 (m, 1H, α-CH), 3.53 - 3.39 (m, 2H, γ-CH_2), 1.93 - 1.68 (m, 2H, β-CH_2) ppm.

The analytical data are identical to the literature.[1, 6]

3.2 Fmoc-Hse(Dmt)-OH

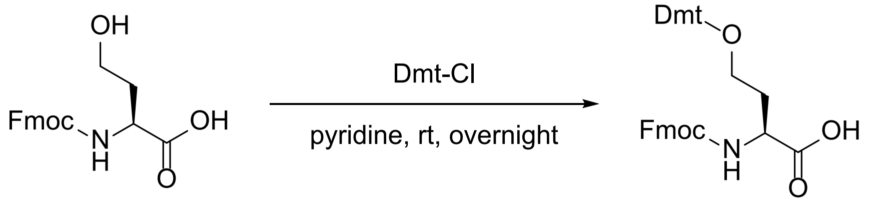

Fmoc-Hse-OH **Fmoc-Hse(Dmt)-OH**

Fmoc-Hse-OH (2.00 g, 5.96 mmol, 1.00 eq.) was dissolved in anhydrous pyridine (30 mL) in an N_2 atmosphere. Dmt-Cl (4.04 g, 11.9 mmol, 2.00 eq.) was added in four portions in 30 min intervals and the mixture was stirred overnight at rt. The solvent was evaporated under reduced pressure. The residue was dissolved in EtOAc (250 mL), washed with water (3 x 150 mL) and brine (150 mL) and dried over Na_2SO_4. After filtration, the solvent was evaporated under reduced pressure. The viscous yellow oil was kept at −21 °C. The crude product was purified by column chromatography (silica gel, DCM:MeOH:DIPEA (95:4:1)). Product fractions were identified by TLC and the solvent was evaporated under reduced pressure. The remaining oil was dried in vacuum to yield a colourless foam.

Yield: 4.42 g, 5.19 mmol, 89 % (as DIPEA salt).

TLC: R_f (DCM/MeOH/DIPEA 95:4:1) = 0.21.

^{1}H NMR (400 MHz, DMSO-d$_6$) δ = 7.89 (d, 2H, J = 7.5 Hz, H_{ar}), 7.67 (t, 2H, J = 6.5 Hz, H_{ar}), 7.45-7.09 (m, 14H, H_{ar}), 6.84 (dd, 4H, J = 8.9, 2.9 Hz, H_{ar}), 4.31-4.13 (m, 3H, CH-CH_2 Fmoc), 4.05 (td, 1H, J = 8.4, 4.0 Hz, α-CH), 3.68 (s, 1H, O-CH_3), 3.67 (s, 1H, O-CH_3), 3.20-3.02 (m, 4H, CH-$(CH_3)_2$ DIPEA, γ-CH_2), 3.02-2.92 (m, 1H, γ-CH_2), 2.60 (q, 3H, J = 7.2 Hz, CH_2-CH_3 DIPEA), 2.16-1.94 (m, 1H, β-CH_2), 1.92-1.73 (m, 1H, β-CH_2), 1.09-0.94 (m, 24H, CH_3 DIPEA) ppm.

The analytical data are identical to the literature.[1]

Abbreviation

Ac	Acetyl
Boc	*tert*-Butoxycarbonyl
Bzl	Benzyl
CD	Circular dichroism
COSY	Correlation spectroscopy
DBU	1,8-Diazabicyclo[5.4.0]undec-7-ene
DCM	Dichloromethane
DHB	2,5-Dihydroxybenzoic acid
DIC	*N*,*N'*-Diisopropylcarbodiimide
DIPEA	*N*,*N*-Diisopropylethylamine
DMF	*N*,*N*-Dimethylformamide
DMSO	Dimethyl sulfoxide
Dmt	4,4'-Dimethoxytrityl
Et_2O	Diethyl ether
EtOAc	Ethyl acetate
Fmoc	9*H*-Fluoren-9-ylmethoxycarbonyl
Fmoc-OSu	*N*-(9*H*-Fluoren-9-ylmethoxycarbonyloxy)-succinimide
HPLC	High performance liquid chromatography
Hse	Homoserine
HSQC	Heteronuclear single quantum coherence
LSF	Late-stage-functionalization
MALDI	Matrix assisted laser desorption ionisation
MBHA	4-Methylbenzhydrylamine
MeCN	Acetonitrile
MeOH	Methanol
MHC	Major histocompatibility complex
MS	Mass spectrometry
MTPI	Methyltriphenoxyphosphonium iodide
Nle	Norleucine
Oxyma	Ethyl cyano(hydroxyimino)acetate
PyBOP	Benzotriazol-1-yloxytripyrrolidinophosphonium hexafluorophosphate
RP	Reversed phase
rt	Room temperature
SPPS	Solid-phase peptide synthesis
TFA	Trifluoroacetic acid
TIPS	Triisopropyl silane
TLC	Thin-layer chromatography
TOF	Time-of-flight
Tren	Tris(2-aminoethyl)amine

4 References

[1] M. Werner, J. Pampel, T. L. Pham, F. Thomas, *Chem. Eur. J.* **2022**, *28*, e202201339.

[2] J. B. Crumpton, W. Zhang, W. L. Santos, *Anal. Chem.* **2011**, *83*, 3548-3554.

[3] a) T. L. Pham, S. Fazliev, P. Baur, P. Comba, F. Thomas, *ChemBioChem* **2023**, *24*, e202200588; b) C. N. Pace, F. Vajdos, L. Fee, G. Grimsley, T. Gray, *Protein Sci.* **1995**, *4*, 2411-2423.

[4] N. J. Greenfield, *Nat. Protoc.* **2006**, *1*, 2876-2890.

[5] N. J. Greenfield, *Nat. Protoc.* **2006**, *1*, 2527-2535.

[6] F. Filira, B. Biondi, L. Biondi, E. Giannini, M. Gobbo, L. Negri, R. Rocchi, *Org. Biomol. Chem.* **2003**, *1*, 3059-3063.
